# Asymmetric synthesis of β-amino cyanoesters with contiguous tetrasubstituted carbon centers by halogen-bonding catalysis with chiral halonium salt

**DOI:** 10.3762/bjoc.21.43

**Published:** 2025-03-12

**Authors:** Yasushi Yoshida, Maho Aono, Takashi Mino, Masami Sakamoto

**Affiliations:** 1 Institute for Advanced Academic Research (IAAR), Chiba University, 1-33, Yayoi-cho, Inage-ku, Chiba 263-8522, Japan; 2 Molecular Chirality Research Center, Graduate School of Engineering, Chiba University, 1-33, Yayoi-cho, Inage-ku, Chiba-shi, Chiba 263-8522, Japan

**Keywords:** asymmetric catalysis, chiral halonium salt, contiguous stereocenters, halogen bonding, Mannich reaction

## Abstract

β-Amino cyanoesters are important scaffolds because they can be transformed into useful chiral amines, amino acids, and amino alcohols. Halogen bonding, which can be formed between halogen atoms and electron-rich chemical species, is attractive because of its unique interaction in organic synthesis. Chiral halonium salts have been found to have strong halogen-bonding-donor abilities and work as powerful asymmetric catalysts. Recently, we have developed binaphthyl-based chiral halonium salts and applied them in several enantioselective reactions, which formed the corresponding products in high to excellent enantioselectivities. In this paper, the asymmetric synthesis of β-amino cyanoesters with contiguous tetrasubstituted carbon stereogenic centers by the Mannich reaction through chiral halonium salt catalysis is presented, which provided the corresponding products in excellent yields with up to 86% ee. To the best of our knowledge, the present paper is the first to report the asymmetric construction of β-amino cyanoesters with contiguous tetrasubstituted carbon stereogenic centers by the catalytic Mannich reaction.

## Introduction

**Figure 1:**
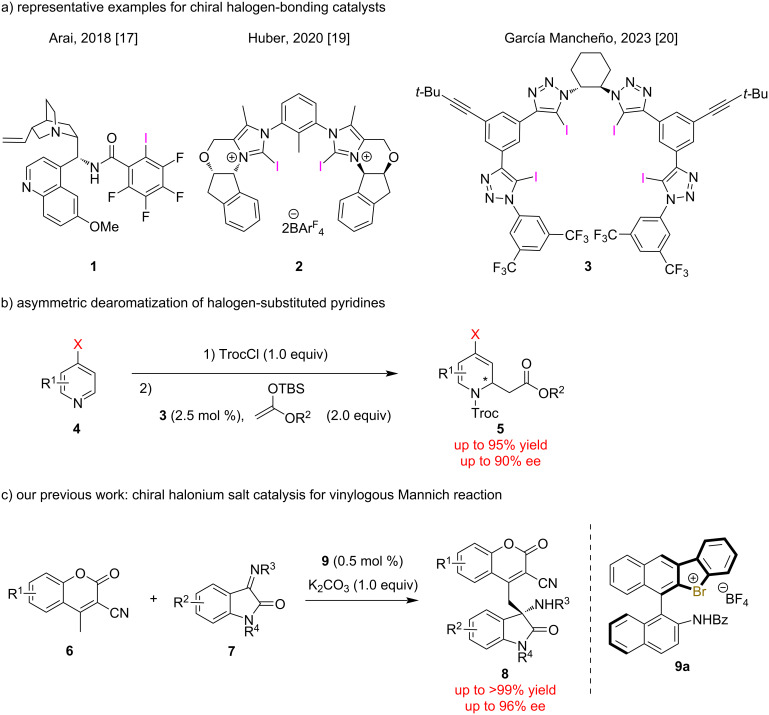
Selected examples and applications of chiral halogen-bonding catalysts.

Halogen bonding (XB) has attracted intense research attention for its unique interaction between halogen atoms and electron-rich substituents [1]. XB has been applied to various fields of chemistry, such as organic chemistry [2–5], organocatalysis [6–7], metal catalysis [8–9], biochemistry [10–11], materials science [12–13], and supramolecular chemistry [14–15], although its successful application to asymmetric catalysis has been limited (Figure 1) [16–20]. In 2018, Arai and co-workers developed chiral amine **1** with an electron-deficient iodine atom, which catalyzed the Mannich reaction in excellent yields and enantioselectivities [17]. In 2020, Huber and co-workers reported the bis(iodoimidazolium) **2**-catalyzed Mukaiyama–aldol reaction of carbonyl compounds with enol silyl ethers, which provided the products in high yields with up to 33% ee [19]. In 2023, García Mancheño and co-workers reported the tetrakis(iodotriazole) **3**-catalyzed dearomatization of halogen-substituted pyridines **4**, which formed the corresponding products **5** in high yields with up to 90% ee (Figure 1b) [20]. Hypervalent halogen compounds have been utilized as highly reactive substrates [21–27] and have recently been reported to work as halogen-bonding catalysts [28–31]. Previously, chiral halonium salts have been utilized in asymmetric catalysis [32–35], and we have developed chiral halonium salts and applied them to asymmetric reactions such as vinylogous Mannich reactions of cyanomethylcoumarins **6** with isatin-derived ketimines **7** [33,35] and 1,2-addition reaction of thiols to ketimine [34], which formed the corresponding products **8** in high yields with high to excellent enantioselectivities (Figure 1c). Despite these successful examples, the construction of only one stereocenter has been reported to date.

**Figure 2:**
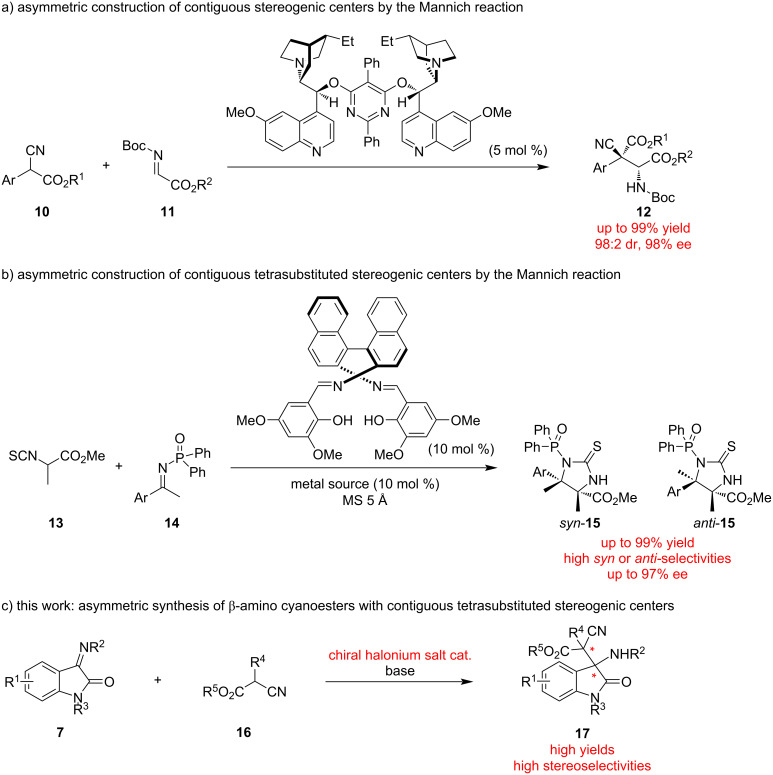
Selected examples for the construction of contiguous tetrasubstituted carbon centers via the Mannich reaction and this work.

The Mannich reaction has great importance because of its utility in the preparation of useful chiral molecules such as amines [36], amino acids [37], and amino alcohols [38]. In this context, their asymmetric syntheses are important and have also been researched mainly using chiral catalysts [39–40]. Previously, the Mannich reaction has been applied in the construction of contiguous stereogenic centers (Figure 2). In 2005, Jørgensen and co-workers reported the enantio- and diastereoselective Mannich reaction of α-cyanoesters with aldimines catalyzed by chiral amines, which provided β-amino cyanoesters in excellent yield and diastereoselectivities with up to 98% ee (Figure 2a) [41]. The Mannich reaction has been also applied in the construction of contiguous tetrasubstituted carbon stereogenic centers [42–46]. In 2011, Shibasaki, Matsunaga and co-workers reported strontium or magnesium-catalyzed stereodivergent asymmetric Mannich reactions of an α-isothiocyanato ester with ketimines, which provided the products in excellent yields and diastereoselectivities with up to 97% ee (Figure 2b) [42]. To the best of our knowledge, the present paper is the first to report the asymmetric construction of β-amino cyanoesters with contiguous tetrasubstituted carbon stereogenic centers by the Mannich reaction, using our originally developed chiral halonium salt catalysis (Figure 2c).

## Results and Discussion

**Scheme 1:**
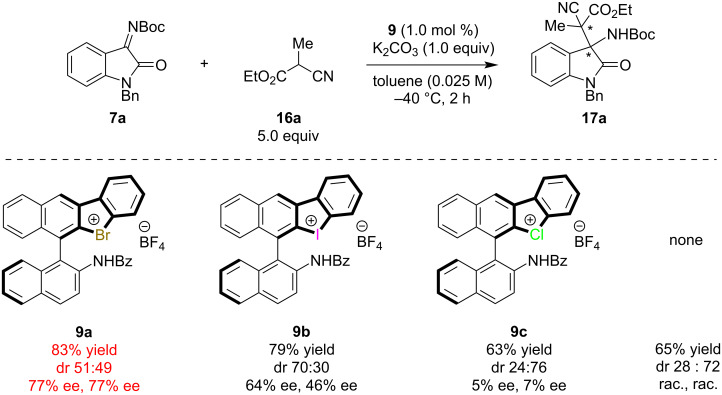
Catalyst screening for the asymmetric Mannich reaction. All yields were determined by ^1^H NMR spectroscopy using 1,3,5-trimethoxybenzene as an internal standard.

Chiral halonium salts **9a**–**c** were prepared according to our previously reported methods [33]. The Mannich reaction of ketimine **7a** and cyanoester **16a** was selected as a benchmark, and catalyst screening was conducted (Scheme 1). The reaction was carried out with 1.0 equivalent of **7a** and 5.0 equivalents of **16a** in the presence of stoichiometric potassium carbonate and 1.0 mol % of **9**. When bromonium salt **9a** was applied to the reaction, the desired product was obtained in 83% yield with 77% ee but almost no diastereoselectivity. The iodonium salt **9b** also worked well and the product was obtained in moderate diastereo- and enantioselectivity, however, chloronium salt **9c** did not show significant catalytic activity, and the product was formed in nearly the same yield as that obtained without a catalyst with low stereoselectivity. From these observations, bromonium salt **9a** was found to be optimal in enantioselectivity, and iodonium salt **9b** was superior in terms of diastereoselectivity. These results can be explained by the strength of halogen bonding: generally, iodo-substituted compounds form stronger halogen bonding with Lewis bases than chloro-substituted ones [1]. Notably, the reaction catalyzed by only 1 mol % of iodonium salt **9b** provided the opposite diastereomer of **17a** as the major product compared with that without a catalyst, which revealed the high catalytic activity of our catalyst. Further reaction conditions optimization was conducted using **9a** as a catalyst (Table 1). Solvent screening was carried out, and it was found to strongly affect the product’s stereoselectivity. Non-polar solvents yielded better results, and toluene was found to be optimal (Table 1, entries 1–6). Polar solvents such as acetonitrile prohibited halogen bonding between **9a** and the chiral halonium salt. Next, the reaction temperature was optimized, and −40 °C was found to be optimal (Table 1, entries 7–9). Further optimization of the reaction conditions (amounts of potassium carbonate and pre-nucleophile, catalyst loading, and concentration) were conducted, and the reaction with 5.0 equivalents of pre-nucleophile and 1.0 equivalent of potassium carbonate in the presence of 1.0 mol % of **9** at 0.025 M of toluene and −40 °C was found to be optimal (Table 1, entries 10–13). Five equivalents of pre-nucleophile are required to obtain higher yields and enantioselectivities.

**Table 1:** Optimization of reaction conditions.^a^

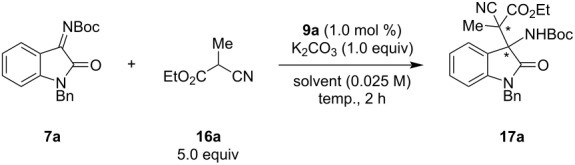				

Entry	Solvent	Temp. (°C)	Yield (%)^b^	dr (ee (%))

1	toluene	−40	83	51 (77% ee):49 (77% ee)
2	Et_2_O	−40	76	57 (70% ee):43 (65% ee)
3	CH_2_Cl_2_	−40	76	58 (51% ee):42 (53% ee)
4	THF	−40	77	58 (32% ee):42 (40% ee)
5	CHCl_3_	−40	68	60 (33% ee):40 (35% ee)
6	CH_3_CN	−40	90	70 (6% ee):30 (14% ee)
7	toluene	0	84	25 (rac.):75 (rac.)
8	toluene	−20	90	52 (70% ee):48 (63% ee)
9^c^	toluene	−80	57	40 (70% ee):60 (75% ee)
10^d^	toluene	−40	87	51 (70% ee):49 (75% ee)
11^e^	toluene	−40	82	51 (73% ee):49 (74% ee)
12^f^	toluene	−40	68	54 (74% ee):46 (73% ee)
13^g^	toluene	−40	74	50 (63% ee):50 (72% ee)

^a^Reactions were conducted using **7a** (1.0 equiv), **16a** (5.0 equiv) and K_2_CO_3_ (1.0 equiv) at the appropriate solvent and temperature for 2 h. ^b^Determined by ^1^H NMR spectroscopy using 1,3,5-trimethoxybenzene as an internal standard. ^c^Reaction conducted for 96 h. ^d^With 10 mol % of K_2_CO_3_. ^e^With 5 mol % of **9a**. ^f^With 1.5 equivalents of **16a**. ^g^Toluene (0.1 M).

**Scheme 2:**
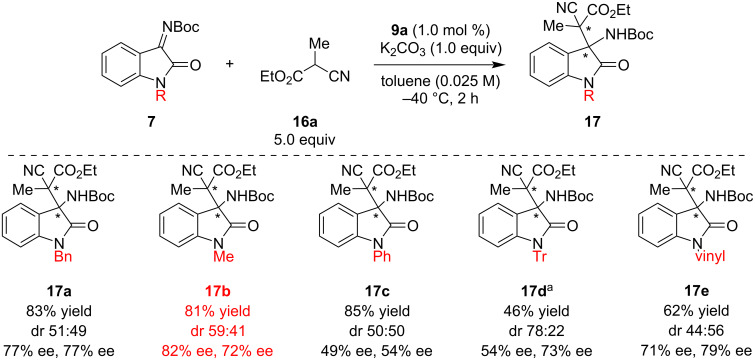
*N*-Protecting group optimization for the asymmetric Mannich reaction. All yields were determined by ^1^H NMR spectroscopy using 1,3,5-trimethoxybenzene as an internal standard. ^a^Reaction conducted for 24 h.

**Scheme 3:**
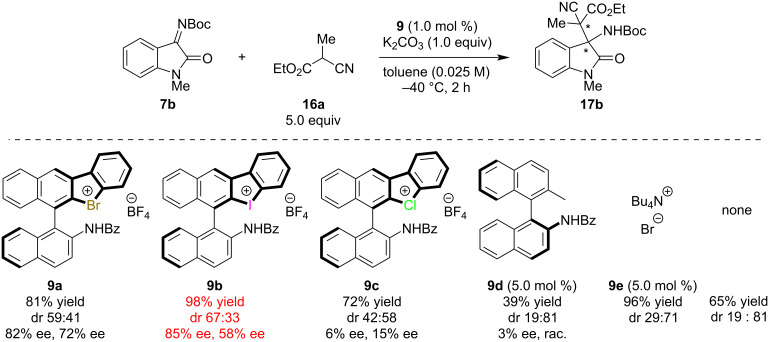
Catalyst screening using **7b** as a substrate. All yields were determined by ^1^H NMR spectroscopy using 1,3,5-trimethoxybenzene as an internal standard.

Next, the optimization of the substituent on the 1-position of imines was conducted (Scheme 2). In most cases, the products were obtained in high yields with moderate to high enantioselectivities; the sterically less-hindered methyl-substituted substrate **7b** was found to be better than the other substrates. The bulky phenyl- or trityl-substituted **7c** and **7d** yielded products with decreased enantioselectivities, likely due to the inhibition of the interaction between the imines and the chiral catalyst by hydrogen and/or halogen bonding. From these observations, the substituent on the 1-position strongly affected the product’s enantioselectivities. Therefore, catalyst screening was conducted again with **7b** as a substrate (Scheme 3). In this case, iodonium salt **9b** showed the best performance, and the product **17b** was formed in 98% yield with a 67 (85% ee):33 (58% ee) diastereomeric ratio. In order to demonstrate the importance of halogen bonding in the catalyst for the present reaction, chiral amide **9d** and tetrabutylammonium bromide (**9e**) were applied as catalysts. The results indicate that **9d** with only hydrogen bonding provided **17b** in a lower yield than without catalyst maybe due to the deactivation of base by acidic amide moiety and with almost no enantioselectivity. Although the addition of a catalytic amount of **9e** accelerated the reaction, the same diastereomer of **17b** as the major product was obtained as for the reaction without a catalyst, which shows the importance of halonium salt moieties in our catalysts. From these results, the substrate scope was conducted using **9b** as a catalyst.

**Scheme 4:**
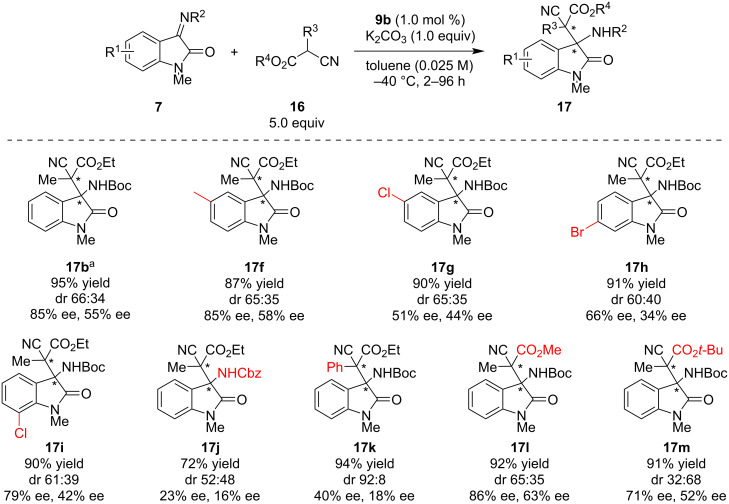
Substrate scope for the asymmetric Mannich reaction using 0.06 mmol of **7**. Isolated product yields are shown. ^a^The result of the reaction using 100.0 mg (0.34 mmol) of **7b**.

First, the scope for the imines was carried out (Scheme 4). 5-Methyl-substituted **7f** provided the corresponding product **17f** in 87% yield and 65:35 diastereomeric ratio with 85% ee and 58% ee for each. 5-Chloro-substituted **7g** formed **17g** in good yield and diastereoselectivity with decreased enantioselectivity, likely due to electronic effects. 6-Bromo- and 7-chloro-substituted substrates also provided **17h** and **17i** in good yields with moderate to good stereoselectivities. Next, Cbz-protected imine **7j** was employed in the present reaction; the stereoselectivity of product **17j** drastically dropped. The scope for the pre-nucleophile showed that phenyl-substituted **16b** provided **17k** in 94% yield with high diastereoselectivity, albeit with decreased enantioselectivities. Methyl ester **16c** and *tert*-butyl ester **16d** were also applied to the present reaction, and products **17l** and **17m** were isolated in high yields with moderate to high stereoselectivities.

**Figure 3:**
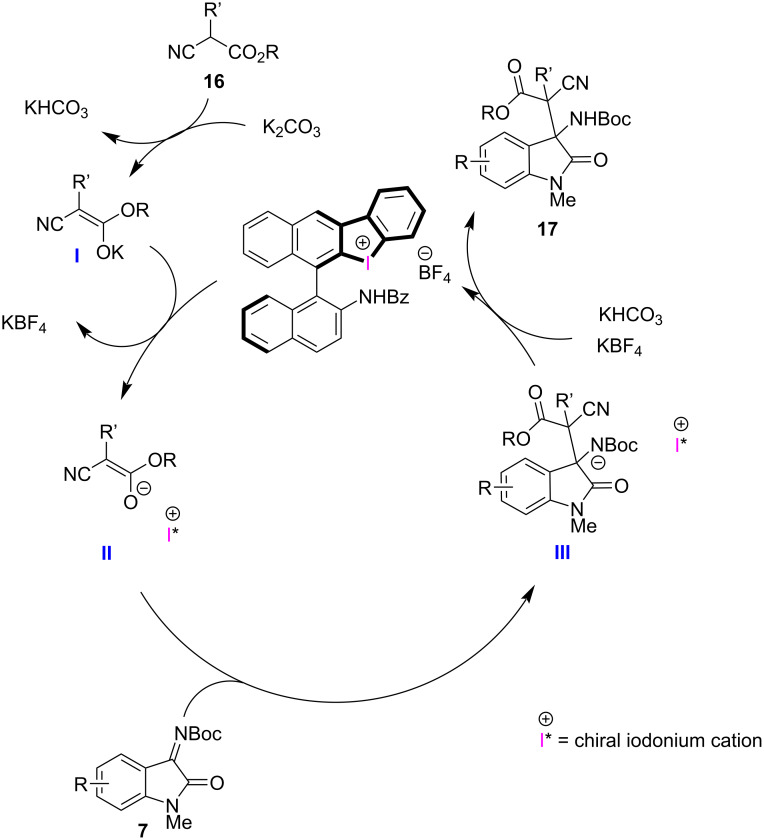
Plausible reaction mechanism.

The plausible reaction mechanism is shown in Figure 3. First, the removal of the acidic proton of the pre-nucleophile by potassium carbonate to form intermediate **I**, which undergoes cation exchange from tetrafluoroborate to the halonium moiety to form chiral ion pair **II**. Attack of the chiral nucleophilic intermediate **II** to imine **7** leads to intermediate **III**. The latter is protonated by in the situ-formed potassium bicarbonate to form the desired product **17**, together with the regenerated chiral halonium salt.

## Conclusion

In conclusion, the enantio- and diastereoselective Mannich reaction was developed by chiral halonium salt catalysis, which provided the corresponding products with contiguous chiral tetrasubstituted carbon centers in excellent yields with up to 86% ee using only 1 mol % catalyst loading. Although the diastereoselectivity of the products were moderate in most cases, the opposite diastereomer was obtained as the major product compared with reactions without a catalyst. To the best of our knowledge, the present paper is the first to report the asymmetric construction of β-amino cyanoesters with contiguous tetrasubstituted carbon stereogenic centers by the catalytic Mannich reaction. Further investigations into the reaction mechanism and product applications are ongoing in our group.

## Supporting Information

File 1 Experimental procedures, characterization data, NMR spectra, and HPLC chromatograms.

## Data Availability

All data that supports the findings of this study is available in the published article and/or the supporting information of this article.
