# Supplementary material for: Asymmetric synthesis of β-amino cyanoesters with contiguous tetrasubstituted carbon centers by halogen-bonding catalysis with chiral halonium salt
Source: Beilstein J Org Chem. 2025 Mar 12;21:547–55. doi: 10.3762/bjoc.21.43 (PMC11912644; doi:10.3762/bjoc.21.43)
Supplement: File 1 — Experimental procedures, characterization data, NMR spectra, and HPLC chromatograms. [file Beilstein_J_Org_Chem-21-547-s001.pdf]

BEILSTEIN JOURNAL OF ORGANIC CHEMISTRY

Supporting Information

for

Asymmetric synthesis of β-amino cyanoesters with contiguous tetrasubstituted carbon centers by halogen-bonding catalysis with chiral halonium salt

Yasushi Yoshida, Maho Aono, Takashi Mino and Masami Sakamoto

Beilstein J. Org. Chem. **2025,** *21,* 547–555. doi:10.3762/bjoc.21.43

Experimental procedures, characterization data, NMR spectra, and HPLC chromatograms

License and Terms: This is a supporting information file under the terms of the Creative Commons Attribution License (https://creativecommons.org/licenses/by/4.0). Please note that the reuse, redistribution and reproduction in particular requires that the author(s) and source are credited and that individual graphics may be subject to special legal provisions.
The license is subject to the *Beilstein Journal of Organic Chemistry* terms and conditions: (https://www.beilstein-journals.org/bjoc/terms)

Table of contents

1. General information S1
2. Synthesis of catalysts S2
3. Synthesis of substrates S2
4. General procedure S4
5. References S16
6. ^{1}H, ^{13}C NMR spectra and HPLC charts S17

1. General information

^{1}H and ^{13}C NMR spectra were recorded with a Bruker AVANCE III- 400M or AVANCE NEO 500 (^{1}H NMR 400, 500 MHz, ^{13}C NMR 100 or 125 MHz). ^{1}H NMR spectra are reported as follows: chemical shift in ppm (δ) relative to the chemical shift of $CHCl_3$ at 7.26 ppm or tetramethylsilane at 0 ppm, integration, multiplicities (s = singlet, d = doublet, t = triplet, q = quartet, m = multiplet), and coupling constants (Hz). ^{13}C NMR spectra reported in ppm (δ) relative to the central line of triplet for $CDCl_3$ at 77 ppm. NMR yields were determined by ^{1}H NMR by comparing the integration values of the internal standard with product values on the condition with D1 of 1.0 s. The short D1 value was employed because comparable product yields with that of D1 with 60.0 s in the reaction of Scheme 3 with **9b** as a catalyst. The diastereoselectivities of products were determined by comparison of ^{1}H NMR integration of each isomer on crude materials. ESI–MS spectra were obtained with a Thermo Fisher, Exactive. FTIR spectra were recorded on a JASCO FT-IR system (FT/IR-460 Plus). Mp were measured with an AS ONE ATM-02. Column chromatography on SiO_2 and neutral SiO_2 was performed with Kanto Silica Gel 60 (40–50 μm). All reactions were carried out under Ar atmosphere unless otherwise noted. Commercially available compounds were used without further purification. All dehydrated solvents were purchased from Wako Pure Chemical Industries, Ltd. or Nacalai Tesque, Inc., and were used without purification.

2. Synthesis of catalysts

Catalysts **9a–d** were synthesized according to the reported procedure.[1,2]

3. Synthesis of substrates

Compounds 7a–d and **7f–j** were prepared according to the reported procedure.[3]

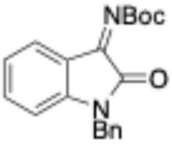

7a

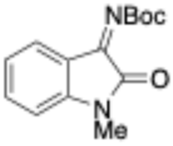

7b

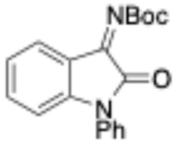

7c

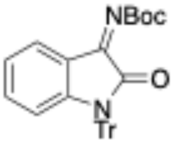

7d

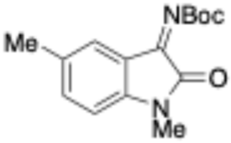

7f

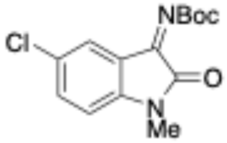

7g

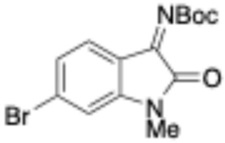

7h

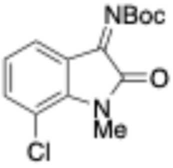

7i

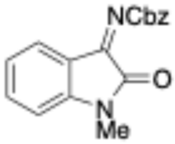

7j

Synthesis of **7e**

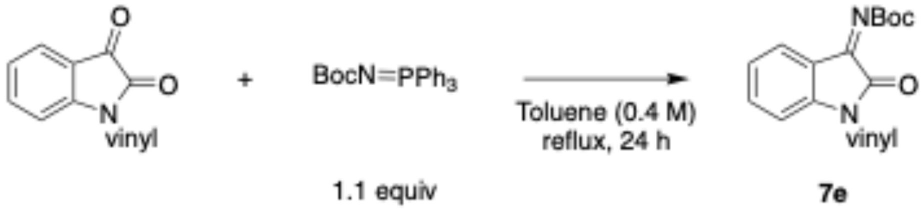

1-Vinylindoline-2,3-dione (1.0 mmol, 1.0 equiv) and *tert*-butyl (triphenyl-λ^5-phosphaneylidene)carbamate (1.1 mmol, 1.1 equiv) were dissolved to toluene (0.4 M), which was refluxed for 24 hours. The solvent was removed by evaporation to give the crude product, which was purified by column chromatography (silica gel, hexane/ethyl acetate) to afford **7e**.

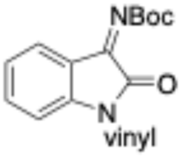

7e

tert-Butyl (*Z*)-(2-oxo-1-vinylindolin-3-ylidene)carbamate (**7e**)

Orange solid, 163.8 mg, 0.67 mmol, 67%

m.p. =139-141 °C; ^{1}H-NMR (500 MHz, CHLOROFORM-D) δ 7.71 (d, *J* = 6.4 Hz, 1H), 7.54-7.50 (m, 1H), 7.18-7.14 (m, 2H), 6.68 (dd, *J* = 15.9, 9.5 Hz, 1H), 5.82 (d, *J* = 15.9 Hz, 1H), 5.15 (d, *J* = 9.5 Hz, 1H), 1.63 (s, 9H);^{13}C-NMR (126 MHz, CHLOROFORM-D) δ 160.3, 156.1, 152.1, 146.3, 135.4, 125.2, 124.6, 124.2, 119.4, 110.4, 105.6, 83.6, 28.0; HRMS (ESI^{+} in MeCN) calcd for $C_{15}H_{17}O_3N_2$ [M+H^{+}] 273.1234, found 273.1230; IR (KBr) ν 3005, 2977, 2937, 1743, 1685, 1614, 1469, 1368, 1275, 1152, 1100, 794, 752, 456 cm^{-1}

Compounds **16a** and **16b** were purchased from TCI. **16d** was synthesized according to the reported procedure.[4] **16c** was synthesized according to the reported procedure[5] and the ^{1}H and ^{13}C NMR data were matched with the reported values.[6]

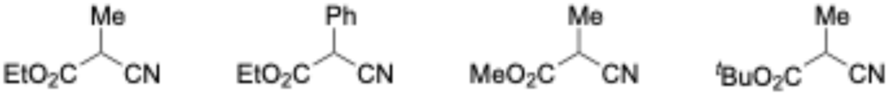

16a **16b** **16c** **16d**

Methyl 2-cyanopropanoate (**16c**)[6]

^{1}H-NMR (500 MHz, CHLOROFORM-D) δ 3.84 (s, 3H), 3.57 (q, *J* = 9.3 Hz, 1H), 1.61 (d, *J* = 9.0 Hz, 3H); ^{13}C-NMR (126 MHz, CHLOROFORM-D) δ 166.9, 117.2, 53.5, 31.2, 15.2.

4. General procedure for the Mannich reaction

・Catalyst screening (Scheme 1).

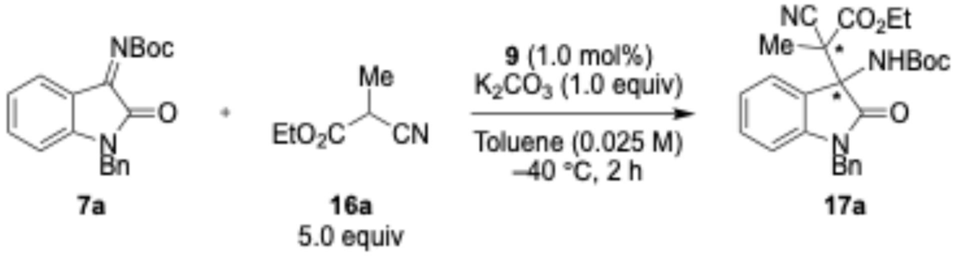

Compound **7a** (0.02 mmol, 1.0 equiv), **9** (1.0 mol %) and K_2CO_3 (0.02 mmol, 1.0 equiv) were added to a reaction vessel and the mixture cooled to −50 °C. After 5 minutes, toluene (0.025 M) was added slowly and the mixture stirred for 5 minutes. Then, **16a** (0.10 mmol, 5.0 equiv) was added and the mixture was warmed to −40 °C and stirred for 2 hours. The reaction was quenched by saturated aqueous solution of ammonium chloride and extracted with CH_2Cl_2, dried over N a_2SO_4, and filtered. The solvent was removed by evaporation to give the crude product. The yields and diastereoselectivities were determined by ^{1}H NMR using 1,3,5-trimethoxybenzene as an internal standard.

Optimization of the reaction conditions (Table 1).

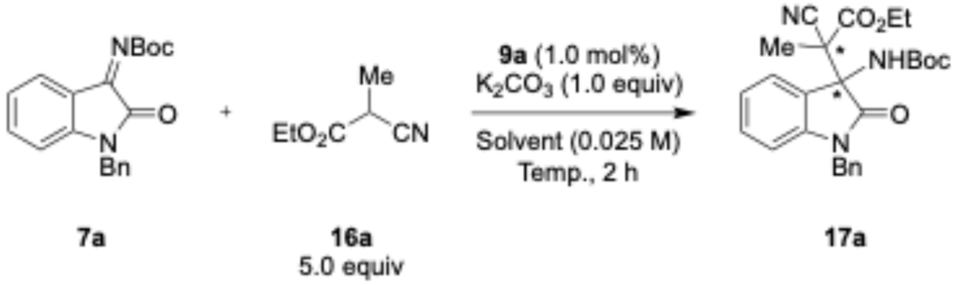

Compound **7a** (0.02 mmol, 1.0 equiv), **9a** (1.0 mol %) and K_2CO_3 (0.02 mmol, 1.0 equiv) were added to a reaction vessel and the mixture was cooled to a temperature 10 °C lower than the test reaction temperature. After 5 minutes, solvent (0.025 M) was added slowly and the mixture stirred for 5 minutes. Then, **16a** (0.10 mmol, 5.0 equiv) was added, the temperature increased to the test reaction temperature and stirred for 2 hours. The reaction was quenched by saturated aqueous solution of ammonium chloride and extracted with CH_2Cl_2, dried over Na_2SO_4, and filtered. The solvent was removed by evaporation to give the crude product. The yields and diastereoselectivities were determined by ^{1}H NMR using 1,3,5-trimethoxybenzene as an internal standard.

・*N*-Protecting group optimization (Scheme 2).

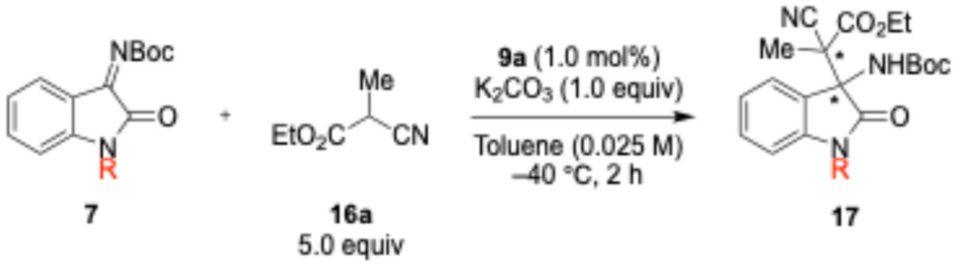

Compound **7** (1.0 equiv), **9a** (1.0 mol %) and K_2CO_3 (1.0 equiv) were added to a reaction vessel and the mixture cooled to −50 °C. After 5 minutes, toluene (0.025 M) was added slowly and the mixture stirred for 5 minutes. Then, **16a** (5.0 equiv) was added, the mixture warmed to −40 °C and stirred for 2 hours. The reaction was quenched with saturated aqueous solution of ammonium chloride, extracted with CH_2Cl_2, dried over Na_2SO_4 and filtered. The solvent was removed by evaporation to give the crude product. The crude product was purified by column chromatography (silica gel, hexane/ethyl acetate) to give **17.**

Ethyl 2-(1-benzyl-3-((*tert*-butoxycarbonyl)amino)-2-oxoindolin-3-yl)-2-cyanopropanoate (**17a**)

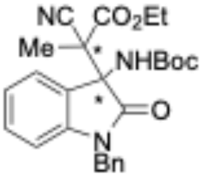

17a

White solid, 38.5 mg, 0.083 mmol, 83% yield, dr 51 : 49, 77% ee, 77% ee

m.p.= 118-120 °C ; ^{1}H-NMR (500 MHz, CHLOROFORM-D) δ (diastereomixture) 7.45-7.41 (m, 2.5H), 7.34-7.31 (m, 2.5H), 7.28-7.23 (m, 2H), 7.09-7.03 (m, 1H), 6.78-6.73 (m, 1H), 6.67 (s, 0.5H), 6.30 (s, 0.5H), 5.04-4.86 (m, 2H), 4.51-4.35 (m, 1H), 4.32-4.21 (m, 1H), 1.76 (s, 1.5H), 1.41 (m, 3H), 1.29-1.23 (m, 10.5H);^{13}C-NMR (126 MHz, CHLOROFORM-D) δ (diastereomixture) 173.1, 172.4, 166.7, 166.2, 153.6, 153.5, 143.6, 143.4, 135.2, 135.2, 130.34, 130.28, 128.71, 128.65, 127.70, 127.65, 127.623(2C), 126.3, 125.5, 123.9, 123.3, 123.0, 122.7, 116.9, 116.4, 109.7, 109.6, 81.0, 80.8, 64.1, 63.9, 63.3, 63.2, 48.9, 48.3, 44.7(2C), 28.04, 27.97, 18.3, 17.2, 13.9, 13.7; HRMS (ESI^{+} in MeCN) calcd for $C_{26}H_{30}O_5N_3$ [M+H^{+}] 464.2180, found 464.2179; IR (KBr) ν 3394, 2964, 2928, 1723, 1613, 1489, 1469, 1456, 1368, 1260, 1163, 1011, 802, 697 cm^{-1}; $[\alpha]^{20}_{D}$ = +46.8 (*c* = 0.300, $CHCl_3$); HPLC (CHIRALPAK ID column, hexane/2-propanol = 80/20, flow rate 1.0 mL/min, 25 °C, 220 nm) Major- (t_{major} = 19.0 min, t_{minor} = 28.6 min), Minor- (t_{major} = 11.5 min, t_{minor} = 13.6 min)

Ethyl 2-(3-((*tert*-butoxycarbonyl)amino)-1-methyl-2-oxoindolin-3-yl)-2-cyanopropanoate (**17b**)

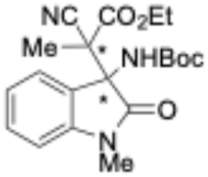

17b

White solid, (cat **9a**) 18.8 mg, 0.049 mmol, 81% yield, dr 59 : 41, 82% ee, 72% ee, (cat **9b**) 125.7 mg, 0.324 mmol (from 0.34 mmol of **7b**), 95% yield, dr 66 : 34, 85% ee, 55% ee

m.p.= 105-107 °C ; ^{1}H-NMR (500 MHz, CHLOROFORM-D) δ (major) 7.41-7.36 (m, 1H), 7.33 (dd, *J* = 7.5, 0.8 Hz, 1H), 7.12-7.07 (m, 1H), 6.88 (t, *J* = 7.0 Hz, 1H), 6.22 (s, 1H), 4.30-4.20 (m, 2H), 3.259 (s, 3H), 1.74 (s, 3H), 1.26-1.23 (m, 12 H); δ (minor) 7.41-7.36 (m, 2H), 7.12-7.07 (m, 1H), 6.89-6.86 (m, 1H), 6.58 (s, 1H), 4.49-4.32 (m, 2H), 3.253 (s, 3H), 1.53 (s, 3H), 1.39 (t, *J* = 7.2 Hz, 3H), 1.25 (s, 9H); ^{13}C-NMR (126 MHz, CHLOROFORM-D) δ (major) 172.6, 166.6, 153.47, 144.3, 130.49, 125.5, 123.9,

122.7, 116.5, 108.59, 81.0, 63.9, 63.2, 49.1, 27.96, 26.58, 18.3, 13.7; δ (minor) 172.1, 166.5, 153.44, 144.1, 130.47, 126.3, 123.2 , 122.9, 116.8, 108.57, 80.9, 64.1, 63.3, 48.4, 28.00, 26.63, 17.2, 13.9; HRMS (ESI^+ in MeCN) calcd for $C_{20}H_{26}O_5N_3$ [$M+H^+$] 388.1867, found 388.1867; IR (KBr) ν 3386, 3338, 2982, 1725, 1611, 1494, 1368, 1255, 1173, 1089, 1017, 983, 762, 689, 540 cm^{-1};(cat **1a**) $[\alpha]^{20}_D$ = +49.3 (*c* = 0.308, $CHCl_3$), (cat **1b**) $[\alpha]^{20}_D$ = +42.0 (*c* = 0.197, $CHCl_3$); HPLC (CHIRALPAK IA column, hexane/ethanol = 95/5, flow rate 1.0 mL/min, 25 °C, 220 nm) Major- (t_{major} = 14.0 min, t_{minor} = 16.1 min), Minor- (t_{major} = 8.4 min, t_{minor} = 9.6 min)

Ethyl 2-(3-((*tert*-butoxycarbonyl)amino)-2-oxo-1-phenylindolin-3-yl)-2-cyanopropanoate (**17c**)

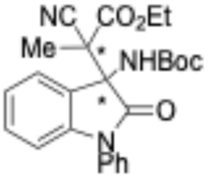

17c

White solid, 22.8 mg, 0.051 mmol, 85% yield, dr 50 : 50, 49% ee, 54% ee

m.p.= 129-131 °C ; ^{1}H-NMR (500 MHz, CHLOROFORM-D) δ (diastereomixture) 7.55-7.51 (m, 2H), 7.49-7.46 (m, 2H), 7.44-7.41 (m, 2H), 7.31-7.28 (m, 1H), 7.14-7.10 (m, 1H), 6.84-6.81 (m, 1H), 6.69 (s, 0.5H), 6.31 (s, 0.5H), 4.49-4.33 (m, 1H),4.25 (q, *J* = 7.2 Hz, 1H), 1.86 (s, 1.5H), 1.65 (s, 1.5H), 1.38 (t, *J* = 7.2 Hz, 1.5H), 1.31-1.30 (m, 9H), 1.22 (t, *J* = 7.2 Hz, 1.5H) ^{13}C-NMR (126 MHz, CHLOROFORM-D) δ (diastereomixture) 172.2, 171.6, 166.8, 166.5, 153.73, 153.69, 144.5, 144.4, 134.03, 134.01, 130.331(2C), 129.657(2C), 128.39, 128.36, 126.63, 126.58, 126.1, 125.3, 124.2, 123.6, 123.2, 123.1, 116.8, 116.5, 109.88, 109.82, 81.2, 81.0, 64.2, 64.0, 63.4, 63.3, 49.2, 48.6, 28.12, 28.05, 18.4, 17.3, 13.9, 13.7; HRMS (ESI^+ in MeCN) calcd for $C_{21}H_{27}O_5N_3Na$ [$M+Na^+$] 472.1843, found 472.1837; IR (KBr) ν 3255, 3140, 2976, 2934, 1735, 1708, 1612, 1502, 1369, 1254, 1163 cm^{-1}; $[\alpha]^{20}_D$ = +30.8 (*c* = 0.530, $CHCl_3$); HPLC (CHIRALPAK IA column, hexane/ethanol = 95/5, flow rate 1.0 mL/min, 25 °C, 220 nm) Major- (t_{major} = 6.8 min, t_{minor} = 7.9 min), Minor- (t_{major} = 9.9 min, t_{minor} =12.7 min)

Ethyl-2-(3-((*tert*-butoxycarbonyl)amino)-2-oxo-1-tritylindolin-3-yl)-2-cyanopropanoate (**17d**)

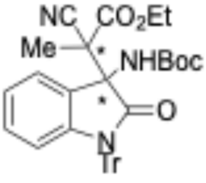

17d

White solid, 17.0 mg, 0.028 mmol, 46% yield, dr 78 : 22, 54% ee, 73% ee

m.p.= 136-138 °C ; ^{1}H-NMR (500 MHz, CHLOROFORM-D) δ (major) 7.62-7.59 (m, 6H), 7.21-7.25 (m, 7H), 7.19-7.15 (m, 3H), 7.03-7.00 (m, 1H), 6.96 (td, *J* = 7.5, 1.0 Hz, 1H), 6.51 (d, *J* = 8.3 Hz, 1H), 6.37 (s, 1H), 4.40-4.32 (m, 1H), 4.237-4.17 (m, 1H), 1.76 (s, 3H), 1.33 (t, *J* = 7.2 Hz, 3H), 1.21 (s, 9H); δ (minor); 7.56-7.51 (m, 6H), 7.25-7.21 (m, 7H), 7.19-7.15 (m, 3H), 7.03-7.00 (m, 2H), 6.83 (s, 1H), 6.37-6.35 (m, 1H), 4.40 -4.32 (m, 1H), 4.30-4.244 (m, 1H), 1.39 (s, 9H), 1.32 (t, *J* = 7.2, 3H), 0.77 (s, 3H); ^{13}C-NMR (126 MHz, CHLOROFORM-D) δ (major) 175.4, 167.4, 153.7, 144.0, 141.76, 129.4, 128.8, 127.5, 126.70, 125.9, 123.8, 122.3, 117.4, 116.4, 80.466, 75.6, 64.0, 63.93, 63.886, 48.4, 28.0, 19.0, 13.9; δ (minor) 172.6, 165.9, 153.9, 143.5, 141.80, 129.1, 128.6, 127.9, 127.6, 126.76, 126.66, 122.8, 122.6, 117.6, 115.7, 80.466, 75.3, 63.886, 63.3, 48.5, 28.2, 16.5, 13.8; HRMS (ESI^{+} in MeCN) calcd for $C_{38}H_{37}O_5N_3Na$ [M+Na^{+}] 638.2625, found 638.2627; IR (KBr) ν 3382, 3061, 2977, 2932, 1734, 1604, 1491, 1368, 1254, 1160, 747, 704, 472 cm^{-1}; $[\alpha]^{20}_{D}$ = −14.4 (*c* = 0.531, $CHCl_3$); HPLC (CHIRALPAK ID column, hexane/ethanol = 99/1, flow rate 1.0 mL/min, 25 °C, 220 nm) Major- (t_{major} = 15.3 min, t_{minor} = 21.7 min), Minor- (t_{major} = 9.7 min, t_{minor} = 10.8 min)

Ethyl-2-(3-((*tert*-butoxycarbonyl)amino)-2-oxo-1-vinylindolin-3-yl)-2-cyanopropanoate (**17e**)

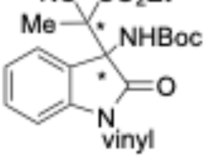

17e

White solid, 14.8 mg, 0.037 mmol, 62% yield, dr 44 : 56, 71% ee, 79% ee

m.p. = 103-105 °C ; ^{1}H-NMR (500 MHz, CHLOROFORM-D) δ (major) 7.46 (d, *J* = 7.6 Hz, 1H), 7.43-7.37 (m, 1H), 7.20-7.13 (m, 2H), 6.78-6.72 (m, 1H), 6.59 (s, 1H), 5.83-5.78 (m, 1H), 5.15-5.13 (m, 1H), 4.49-4.34 (m, 2H), 1.49 (s, 3H), 1.39 (t, *J* = 7.2 Hz, 3H), 1.22 (s, 9H); δ (minor) 7.43-7.37 (m, 2H), 7.20-

7.13 (m, 2H), 6.78-6.72 (m, 1H), 6.15 (s, 1H), 5.83-5.78 (m, 1H), 5.15-5.13 (m, 1H), 4.29-4.17 (m, 2H), 1.74 (s, 3H), 1.25-1.21 (m, 12H); ^{13}C-NMR (126 MHz, CHLOROFORM-D) δ (major) 171.2, 166.1, 153.32, 142.2, 130.50, 125.982, 125.3, 124.0, 123.4, 116.6, 109.79, 105.2, 81.2, 64.2, 63.2, 48.5, 27.90, 17.3, 13.9; δ (minor) 171.6, 166.2, 153.37, 142.3, 130.55, 126.2, 125.982, 125.3, 124.2, 123.5, 116.3, 109.76, 105.1, 81.4, 64.0, 62.6, 49.6, 27.93, 18.1, 13.7; HRMS (ESI$^+$ in MeCN) calcd for $C_{21}H_{25}O_5N_3Na$ [M+Na$^+$] 422.1686, found 422.1678; IR (KBr) ν 3385, 3256, 3139, 2979, 1741, 1706, 1642, 1608, 1487, 1468, 1367, 1253, 1162, 757 cm^{-1}; $[\alpha]^{20}_D$ = +37.3 (*c* = 0.204, CHCl$_3$); HPLC (CHIRALPAK IA column, hexane/ethanol = 95/5, flow rate 1.0 mL/min, 25 °C, 220 nm) Major- (t_{major} = 8.2 min, t_{minor} = 9.3 min), Minor- (t_{major} = 15.8 min, t_{minor} = 16.9 min)

・Catalyst screening with **7b** (Scheme 3).

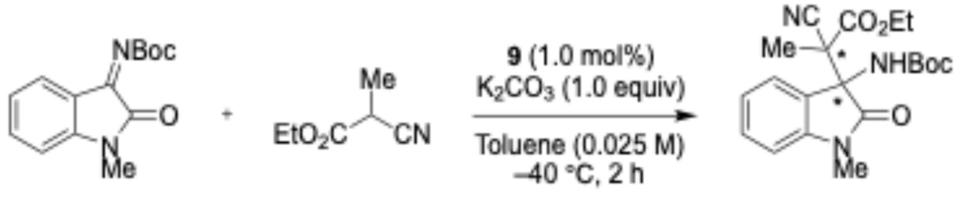

7b **16a** 5.0 equiv **17b**

Compound **7b** (0.02 mmol, 1.0 equiv), **9** (1.0 mol %) and K_2CO_3 (0.02 mmol, 1.0 equiv) were added to a reaction vessel and the mixture cooled to −50 °C. After 5 minutes, toluene (0.025 M) was added slowly and stirred for 5 minutes. Then, **16a** (0.10 mmol, 5.0 equiv) was added and the mixture warmed to −40 °C and stirred for 2 hours. The reaction was quenched with saturated aqueous solution of ammonium chloride, extracted with CH_2Cl_2, dried over Na_2SO_4, and filtered. The solvent was removed by evaporation to give the crude product. The yields and diastereoselectivities were determined by ^{1}H NMR using 1,3,5-trimethoxybenzene as an internal standard.

・Substrate scope (Scheme 4).

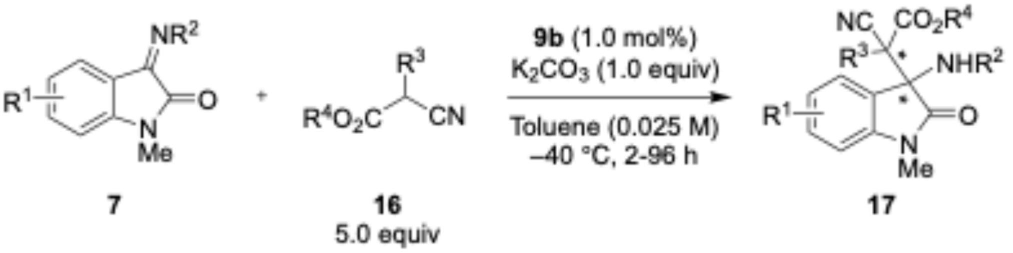

Compound **7** (0.06 mmol, 1.0 equiv), **9b** (1.0 mol %) and K_2CO_3 (0.06 mmol, 1.0 equiv) were added to a reaction vessel and the mixture cooled to −50 °C. After 5 minutes, toluene (0.025 M) was added slowly and the mixture stirred for 5 minutes. Then, **16** (0.3 mmol, 5.0 equiv) was added, the mixture warmed to −40 °C and stirred for the appropriate time. The reaction was quenched with saturated aqueous solution of ammonium chloride, extracted with CH_2Cl_2, dried over Na_2SO_4, and filtered. The solvent was removed by evaporation to give the crude product. The crude product was purified by column chromatography (silica gel, hexane/ethyl acetate) to give **17.**

Ethyl 2-(3-((*tert*-butoxycarbonyl)amino)-1,5-dimethyl-2-oxoindolin-3-yl)-2-cyanopropanoate (**17f**)

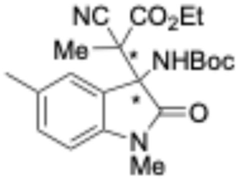

17f

Reaction time: 2 hours

White solid, 21.0 mg, 0.052 mmol, 87% yield, dr 65 : 35, 85% ee, 58% ee

m.p.= 118-120 °C ; ^{1}H-NMR (500 MHz, CHLOROFORM-D) δ (major) 7.19-7.16 (m, 1H),7.123-7.118 (m, 1H), 6.78-6.75 (m, 1H), 6.22 (s, 1H), 4.30-4.21 (m, 2H), 3.23 (s, 3H), 2.34 (s, 3H), 1.74 (s, 3H), 1.33-1.11 (m, 12H); δ (minor); 7.19-7.16 (m, 2H), 6.78-6.75 (m, 1H), 6.56 (s, 1H), 4.50-4.44 (m, 1H), 4.37-4.31 (m, 1H), 2.34 (s, 3H), 1.51 (s, 3H), 1.39 (t, J = 7.2 Hz, 3H), 1.25 (s, 9H); ^{13}C-NMR (126 MHz, CHLOROFORM-D) δ (major) 172.5, 166.7, 153.51, 141.9, 132.2, 130.75, 125.4, 124.6, 116.5, 108.33, 81.0, 63.8, 63.3, 49.1, 27.98, 26.57, 21.17, 18.3, 13.7; δ (minor) 171.9, 166.5, 153.46, 141.7, 132.8, 130.72, 126.3, 123.6, 116.8, 108.30, 80.8, 64.0, 63.4, 48.4, 28.02, 26.63, 21.16, 17.2, 13.9; HRMS (ESI$^+$ in MeCN) calcd for $C_{21}H_{27}O_5N_3Na$ [M+Na$^+$] 424.1843, found 424.1837; IR (KBr) ν 3310, 2982, 2197,

1707, 1618, 1502, 1369, 1255, 1172, 985, 818, 553 cm^{-1}; $[\alpha]^{20}_D$ = +39.5 (*c* = 0.656, $CHCl_3$); HPLC (CHIRALPAK IA-3 column, hexane/ethanol = 95/5, flow rate 1.0 mL/min, 25 °C, 220 nm) Major- (t_{major} = 12.2 min, t_{minor} = 16.1 min), Minor- (t_{major} = 8.4 min, t_{minor} = 9.6 min)

Ethyl 2-(3-((*tert*-butoxycarbonyl)amino)-5-chloro-1-methyl-2-oxoindolin-3-yl)-2-cyanopropanoate (**17g**)

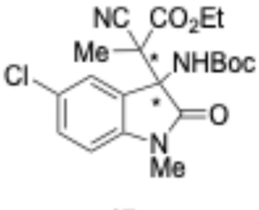

17g

Reaction time: 2 hours

White solid, 22.8 mg, 0.054 mmol, 90% yield, dr 65 : 35, 51% ee, 44% ee

m.p.= 130-132 °C; ^{1}H-NMR (500 MHz, CHLOROFORM-D) δ (major) 7.38-7.35 (m, 1H), 7.30 (d, *J* = 1.9 Hz, 1H), 6.81 (d, *J* = 8.6 Hz, 1H), 6.28 (s, 1H), 4.30-4.26 (m, 2H), 3.25 (s, 3H), 1.74 (s, 3H), 1.36-1.26 (m, 12H) δ (minor); 7.38-7.35 (m, 1H), 7.29 (d, *J* = 1.9 Hz, 1H), 6.82 (d, *J* = 8.3 Hz, 1H), 6.56 (s, 1H), 4.51-4.45 (m, 1H), 4.38-4.33 (m, 1H), 3.25 (s, 3H), 1.61 (s, 3H), 1.39 (t, *J* = 7.2 Hz, 3H), 1.29 (s, 9H); ^{13}C-NMR (126 MHz, CHLOROFORM-D) δ (major) 172.3, 166.5, 153.5, 142.9, 130.39, 128.1, 127.1, 124.3, 116.1, 109.582, 81.4, 64.1, 63.21, 48.8, 28.00, 26.70, 18.3, 13.7; δ (minor) 171.6, 166.6, 153.4, 142.7, 130.41, 128.5, 128.0, 123.4, 116.3, 109.582, 81.2, 64.3, 63.16, 48.3, 28.04, 26.77, 17.2, 13.9; HRMS (ESI^+ in MeCN) calcd for $C_{20}H_{24}O_5N_3ClNa$ [$M+Na^+$] 444.1297, found 444.1286; IR (KBr) ν 3316, 2984, 2940, 1709, 1609, 1489, 1369, 1260, 1171, 820, 546 cm^{-1}; $[\alpha]^{20}_D$ = +14.4 (*c* = 0.400, $CHCl_3$); HPLC (CHIRALPAK IA-3 column, hexane/2-propanol = 97/3, flow rate 1.0 mL/min, 25 °C, 220 nm) Major- (t_{major} = 24.8 min, t_{minor} = 30.5 min), Minor- (t_{major} = 20.4 min, t_{minor} = 14.8 min)

Ethyl 2-(6-bromo-3-((*tert*-butoxycarbonyl)amino)-1-methyl-2-oxoindolin-3-yl)-2-cyanopropanoate (**17h**)

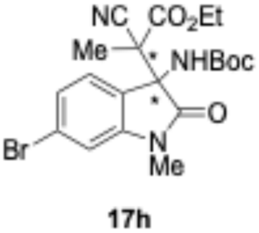

17h

Reaction time: 2 hours

White solid, 25.4 mg, 0.055 mmol, 91% yield, dr 60 : 40, 66% ee, 34% ee

m.p.= 117-119 °C; ^{1}H-NMR (500 MHz, CHLOROFORM-D) δ (major) 7.25-7.19 (m, 2H), 7.02 (d, J = 1.3 Hz, 1H), 6.20 (s, 1H), 4.31-4.21 (m, 2H), 3.239 (s, 3H), 1.74 (s, 3H), 1.28-1.25 (m, 12H) δ (minor) 7.25-7.19 (m, 2H), 7.04 (d, J = 1.3 Hz, 1H), 6.59 (s, 1H), 4.48- 4.41 (m, 1H), 4.39 - 4.33 (m, 1H) 3.235 (s, 3H), 1.54 (s, 3H), 1.39 (t, J = 7.2 Hz, 3H), 1.28 (s, 9H); ^{13}C-NMR (126 MHz, CHLOROFORM-D) δ (major) 171.9, 166.4, 153.48, 145.6, 125.546, 125.1, 124.4, 124.2, 116.2, 112.230, 81.4, 64.0, 62.9, 48.9, 28.01, 26.72, 18.3, 13.7; δ (minor) 172.5, 166.3, 153.46, 145.5, 126.0, 125.546, 124.5, 124.3, 116.5, 112.230, 81.2, 64.3, 63.1, 48.2, 28.05, 26.78, 17.2, 13.9; HRMS (ESI$^+$ in MeCN) calcd for $C_{20}H_{25}O_5N_3Br$ [M+H$^+$] 466.0972, found 466.0971; IR (KBr) ν 3374, 2996, 2980, 2941, 1734, 1608, 1495, 1367, 1281, 1164, 1016 cm^{-1}; $[\alpha]^{20}_D$ = +10.3 (c = 0.320, $CHCl_3$); HPLC (CHIRALPAK IA-3 column, hexane/2-propanol = 97/3, flow rate 0.9 mL/min, 25 °C, 220 nm) Major- (t_{major} = 23.1 min, t_{minor} = 32.7 min), Minor- (t_{major} = 15.8 min, t_{minor} = 14.7 min)

Ethyl 2-(3-((*tert*-butoxycarbonyl)amino)-7-chloro-1-methyl-2-oxoindolin-3-yl)-2-cyanopropanoate (**17i**)

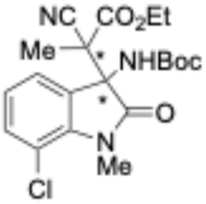

17i

Reaction time: 2 hours

White solid, 22.66 mg, 0.054 mmol, 90% yield, dr 61 : 39, 79% ee, 42% ee

m.p.= 115-117 °C; ^{1}H-NMR (500 MHz, CHLOROFORM-D) δ (major); 7.32-7.29 (m, 1H), 7.24 (dd, J = 7.6, 1.0 Hz, 1H), 7.04-6.98 (m, 1H), 6.19 (s, 1H), 4.30-4.20 (m, 2H), 3.619 (s, 3H), 1.73 (s, 3H), 1.27-1.23 (m, 12H); δ (minor) 7.32-7.29 (m, 2H), 7.04-6.98 (m, 1H), 6.60 (s, 1H), 4.48 -4.32 (m, 2H), 3.609 (s, 3H), 1.49 (s, 3H), 1.39 (t, J = 7.2 Hz, 3H), 1.27 (s, 9H); ^{13}C-NMR (126 MHz, CHLOROFORM-D) δ (major) 173.0, 166.2, 153.371, 140.2, 132.77, 128.5, 123.3, 122.3, 116.23, 116.15, 81.4, 64.0, 62.6, 49.3, 30.05, 27.96, 18.2, 13.7; δ (minor) 172.5, 166.1, 153.371, 140.1, 132.79, 129.3, 123.9, 121.4, 116.5, 116.18, 81.2, 64.3, 63.0, 48.4, 30.08, 28.00, 17.2, 13.9; HRMS (ESI$^+$ in MeCN) calcd for $C_{20}H_{24}O_5N_3ClNa$ [M+Na$^+$] 444.1297, found 444.1288. ; IR (KBr) ν 3385, 2991, 1734, 1610, 1466, 1367, 1257, 1164, 1111, 1056, 739 cm^{-1}; $[\alpha]^{20}_D$ = +16.8 (c = 0.198, $CHCl_3$); HPLC (CHIRALPAK IA-3 column, hexane/2-

propanol = 94/6, flow rate 1.0 mL/min, 25 °C, 220 nm) Major- (t_{major} = 37.6 min, t_{minor} = 35.4 min), Minor- (t_{major} = 10.0 min, t_{minor} = 12.0 min)

Ethyl 2-(3-(((benzyloxy)carbonyl)amino)-1-methyl-2-oxoindolin-3-yl)-2-cyanopropanoate (**17j**)

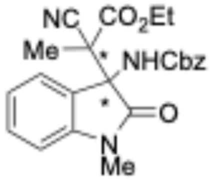

17j

Reaction time: 2 hours

White solid, 18.22mg, 0.052 mmol, 72% yield, dr 58 : 42, 23% ee, 16% ee

m.p.= 71-73°C ; ^{1}H-NMR (500 MHz, CHLOROFORM-D) δ (major) 7.43-7.32 (m, 5H), 7.21 (br, 2H), 7.13-7.08 (m, 1H), 6.85 (br, 1H), 6.60 (s, 1H), 5.02-4.90 (m, 2H) , 4.31-4.22 (m, 2H), 3.21 (s, 3H), 1.72 (s, 3H), 1.25 (t, J = 7.2 Hz, 3H); δ (minor) 7.43 -7.32 (m, 5H), 7.21 (br, 2H), 7.13 -7.08 (m, 1H), 6.93 (s, 1H), 6.85 (br, 1H), 5.02-4.90 (m, 2H), 4.47-4.40 (m, 1H), 4.39 -4.33 (m, 1H), 3.21 (s, 3H), 1.56 (s, 3H), 1.37 (t, J = 7.2 Hz, 3H); ^{13}C-NMR (126 MHz, CHLOROFORM-D) δ (major) 172.3, 166.562, 154.28, 144.4, 135.2, 130.76, 128.482, 128.280, 128.17, 124.7, 124.0, 122.8, 116.2, 108.764, 67.57, 64.0, 63.2, 48.8, 26.57, 18.2, 13.7; δ (minor) 171.7, 166.562, 154.24, 144.2, 135.3, 130.75, 128.482, 128.32, 128.280, 125.7, 123.3, 123.1, 116.5, 108.764, 67.49, 64.2, 63.3, 48.2, 26.64, 17.2, 13.9 HRMS (ESI^{+} in MeCN) calcd for $C_{23}H_{23}O_5N_3Na$ [M+Na^{+}] 444.1530, found 444.1518; IR (KBr) ν 3374, 2937, 1730, 1613, 1495, 1374, 1255, 1089, 1002, 755, 699 cm^{-1}; $[\alpha]^{20}_{D}$ = +2.8 (c = 0.240, $CHCl_3$); HPLC (CHIRALPAK AD-H column, hexane/ethanol = 96.5/3.5, flow rate 1.0 mL/min, 25 °C, 220 nm) Major- (t_{major} = 73.5 min, t_{minor} = 67.9 min), Minor- (t_{major} = 30.5 min, t_{minor} = 52.2 min)

Ethyl 2-(3-((*tert*-butoxycarbonyl)amino)-1-methyl-2-oxoindolin-3-yl)-2-cyano-2-phenylacetate (**17k**)

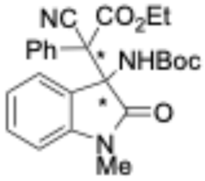

17k

Reaction time: 2 hours

White solid, 25.43 mg, 0.057 mmol, 94% yield, dr 92 : 8, 40% ee, 18% ee

m.p.= 150-152 °C ; ^{1}H-NMR (500 MHz, CHLOROFORM-D) δ (major) 7.41-7.28 (m, 6H), 6.92-6.87 (m, 2H), 6.69 (d, J = 7.6 Hz, 2H), 4.43 -4.30 (m, 2H), 3.04 (s, 3H), 1.30 (t, J = 7.0 Hz, 3H), 1.23 (s, 9H); δ (minor) 7.47-7.45 (m, 1H), 7.21-7.18 (m, 3H), 7.02 (td, J = 7.5, 1.0 Hz, 2H), 6.92-6.87 (m, 2H), 6.49 (d, J = 7.6 Hz, 2H), 4.57-4.45 (m, 2H), 3.04 (s, 3H), 1.38 (t, J = 7.2 Hz, 3H), 1.23 (s, 9H);^{13}C-NMR (126 MHz, CHLOROFORM-D) δ (major) 172.6, 165.8, 153.7, 144.6, 130.3, 129.7, 128.8, 128.13, 128.03, 125.6, 124.6, 122.1, 114.9, 108.2, 80.8, 66.0, 64.3, 58.1, 28.0, 26.3, 13.7; HRMS (ESI^{+} in MeCN) calcd for $C_{25}H_{27}O_5N_3Na$ [M+Na^{+}] 472.1843, found 472.1825; IR (KBr) ν 3370, 3265, 3149, 3061, 2979, 2932, 1706, 1613, 1491, 1372, 1247, 1160, 1017, 754, 696 cm^{-1}; $[\alpha]^{20}_{D}$ = +20.0 (c = 0.303, $CHCl_3$); HPLC (CHIRALPAK ID column, hexane/ethanol = 95/5, flow rate 1.0 mL/min, 25 °C, 220 nm) Major- (t_{major} =27.5 min, t_{minor} = 30.7 min), Minor- (t_{major} = 17.9 min, t_{minor} = 23.8 min)

Methyl 2-(3-((*tert*-butoxycarbonyl)amino)-1-methyl-2-oxoindolin-3-yl)-2-cyanopropanoate (**17l**)

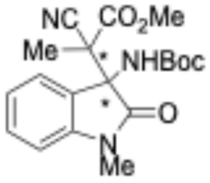

17l

Reaction time: 2 hours

White solid, 20.55 mg, 0.055 mmol, 92% yield, dr 65 : 35, 86% ee, 63% ee

m.p.= 131-133 °C; ^{1}H-NMR (500 MHz, CHLOROFORM-D) δ (major) 7.41-7.37 (m, 1H), 7.29 (dd, J = 7.5, 0.8 Hz, 1H), 7.12-7.07 (m, 1H), 6.88 (d, J = 7.6 Hz, 1H), 6.24 (s, 1H), 3.83 (s, 3H), 3.26 (s, 3H), 1.74 (s, 3H), 1.24 (s, 9H); δ (minor) 7.41-7.37 (m, 1H), 7.34 (d, J = 7.3 Hz, 1H), 7.12-7.07 (m, 1H), 6.88 (d, J = 7.6 Hz, 1H), 6.52 (s, 1H), 3.93 (s, 3H), 3.25 (s, 3H), 1.55 (s, 3H), 1.24 (s, 9H); ^{13}C-NMR (126 MHz, CHLOROFORM-D) δ (major) 172.8, 167.4, 153.5, 144.3, 130.53, 125.3, 123.9, 122.75, 116.2, 108.7, 81.1, 63.4, 54.2, 48.8, 27.95, 26.58, 18.3; δ (minor) 172.0, 167.2, 153.4, 144.1, 130.49, 126.3, 123.3, 122.77, 116.6, 108.6, 80.9, 63.3, 54.3, 48.5, 27.98, 26.64, 17.2; HRMS (ESI^{+} in MeCN) calcd for $C_{19}H_{23}O_5N_3Na$ [M+Na^{+}] 396.1530, found 396.1523; IR (KBr) ν 3398, 2982, 1720, 1611, 1493, 1374, 1256, 1171, 1087, 1025, 977, 762, 690, 539 cm^{-1}; $[\alpha]^{20}_{D}$ = +39.5 (c = 0.200, $CHCl_3$); HPLC (CHIRALPAK IA-3 column, hexane/ethanol = 97/3, flow rate 1.0 mL/min, 25 °C, 220 nm) Major- (t_{major} = 26.1 min, t_{minor} = 34.0 min), Minor- (t_{major} = 16.2 min, t_{minor} = 18.6 min)

tert-Butyl-2-(3-((*tert*-butoxycarbonyl)amino)-1-methyl-2-oxoindolin-3-yl)-2-cyanopropanoate (**17m**)

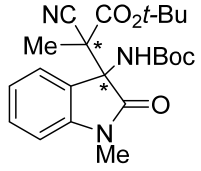

17m

Reaction time: 24 hours

White solid, 22.71 mg, 0.055 mmol, 91% yield, dr 32 : 68, 71% ee, 52% ee

m.p.= 150-152 °C ; ^{1}H-NMR (500 MHz, CHLOROFORM-D) δ (major) 7.43-7.35 (m, 2H), 7.12-7.07 (m, 1H), 6.87 (d, *J* = 7.6 Hz, 1H), 6.70 (s, 1H), 3.25 (s, 3H), 1.60 (s, 9H), 1.46 (s, 3H), 1.24 (s, 9H), δ (minor); 7.43-7.35 (m, 2H), 7.12-7.07 (m, 1H), 6.84 (d, *J* = 7.9 Hz, 1H), 6.11 (s, 1H), 3.24 (s, 3H), 1.74 (s, 3H), 1.38 (s, 9H), 1.27 (s, 9H);^{13}C-NMR (126 MHz, CHLOROFORM-D) δ (major) 172.2, 164.87, 153.5, 144.1, 130.37, 126.6, 123.1(2C), 117.1, 108.46, 86.6, 80.7, 63.3, 48.8, 28.03, 27.7, 26.6, 17.3; δ (minor) 172.5, 164.87, 153.4, 144.3, 130.35, 125.8, 123.9, 122.6, 117.0, 108.46, 85.8, 80.7, 62.5, 50.2, 27.96, 27.4, 26.5, 18.2; HRMS (ESI^{+} in MeCN) calcd for $C_{22}H_{29}O_5N_3K$ [M+K^{+}] 454.1739, found 454.1733; IR (KBr) ν 3442, 3267, 3141, 2971, 2937, 1701, 1612, 1475, 1371, 1278, 1160, 754 cm^{-1}; $[\alpha]^{20}_{D}$ = +45.2 (*c* = 0.305, $CHCl_3$); HPLC (CHIRALPAK IA-3 column, hexane/ethanol = 97/3, flow rate 1.0 mL/min, 25 °C, 254 nm) Major- (t_{major} = 7.36 min, t_{minor} = 9.46 min), Minor- (t_{major} =16.4 min, t_{minor} = 22.8 min)

References

1) Y. Yoshida *et al.*, *ACS Catal.*, **2021**, *11*, 13028.
2) A. Tsuchihashi, S. Shirakawa, *Synlett,* **2019**, *30*, 1662.
3) (a) J. Veselý *et al.*, *Org. Biomol. Chem.*, **2017**, *15*, 9071; (b) R. Šebesta *et al.*, *ACS Sustainable Chem. Eng.* **2020**, *8*, 14417; (c) S. Nakamura *et al., Chem. Eur. J.,* **2013**, *19,* 7304; (d) R.Wang *et al.,Org. Lett.,* **2012**, *14*, 2512; (e) J. Xu and H. Ren *et al., J. Org. Chem.,* **2020**, *85*, 3894.
4) (a) S. Mahapatra *et al., Angew. Chem. Int. Ed.*, **2015**, *54*, 6032; (b) G. D. Chen and X. Zhang *et al., J. Am. Chem. Soc.,* **2021**, *143*, 2477.
5) X. Zhang and J. Ren *et al., Science,* **2019**, *363,* 400.
6) E. S. Stratford *et al., J. Med. Chem.,* **1983**, *26,* 1463.

5. ^{1}H-, ^{13}C-NMR spectra and HPLC charts.

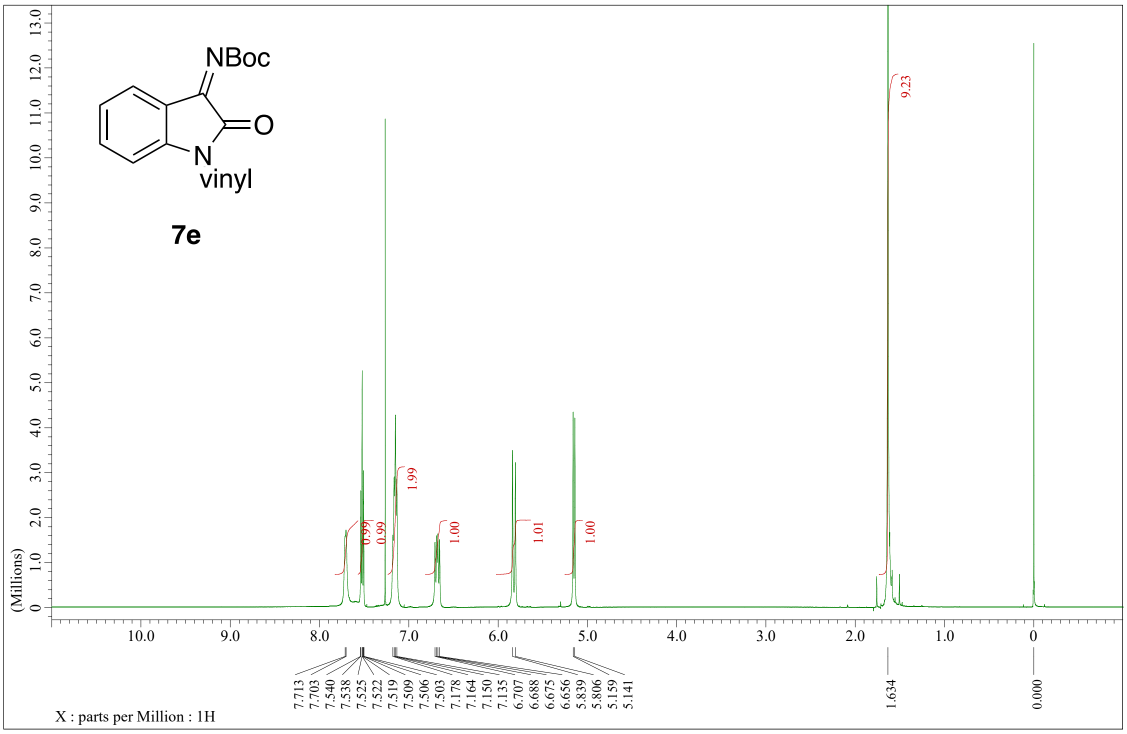

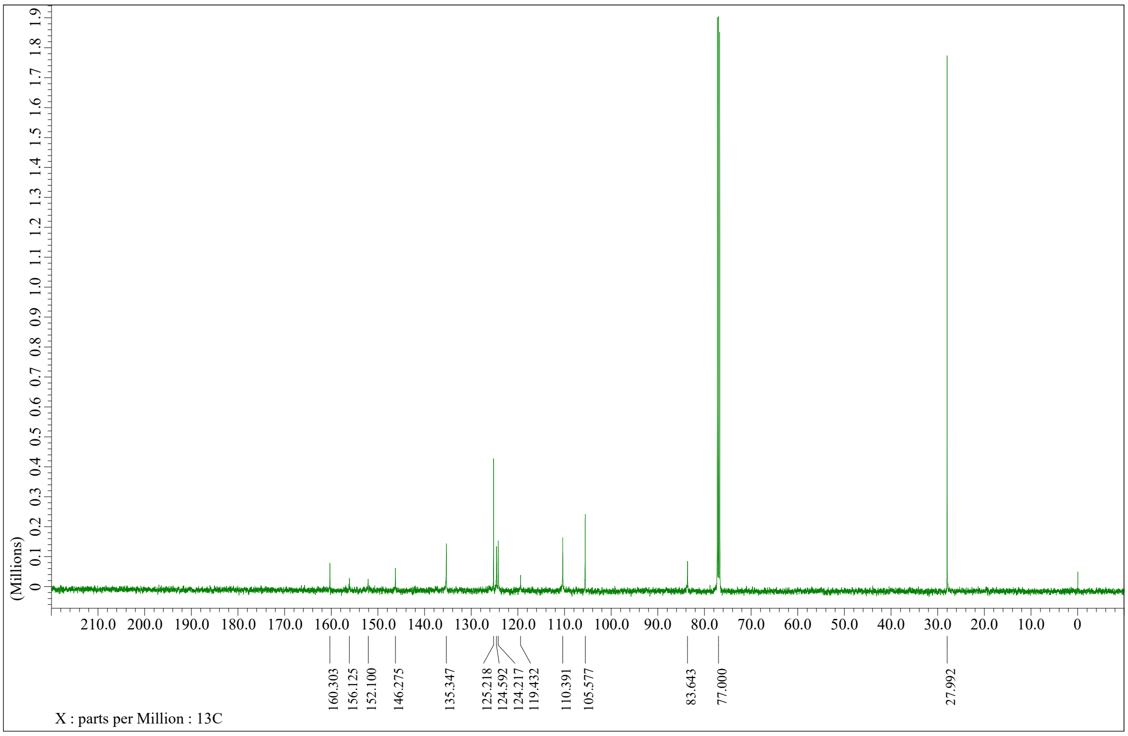

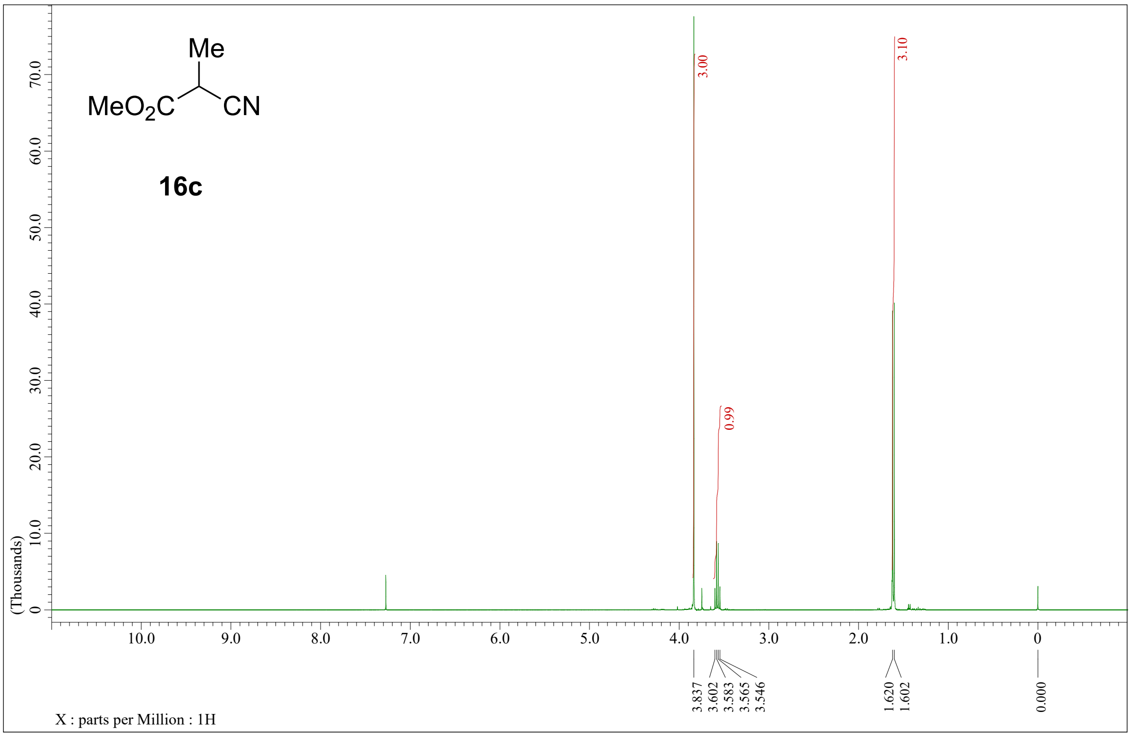
Me
MeO2C
CN
16c
3.00
0.99
3.10
(Thousands)
70.0
60.0
50.0
40.0
30.0
20.0
10.0
0
10.0
9.0
8.0
7.0
6.0
5.0
4.0
3.0
2.0
1.0
0
3.837
3.602
3.583
3.565
3.546
1.620
1.602
0.000
X : parts per Million : 1H

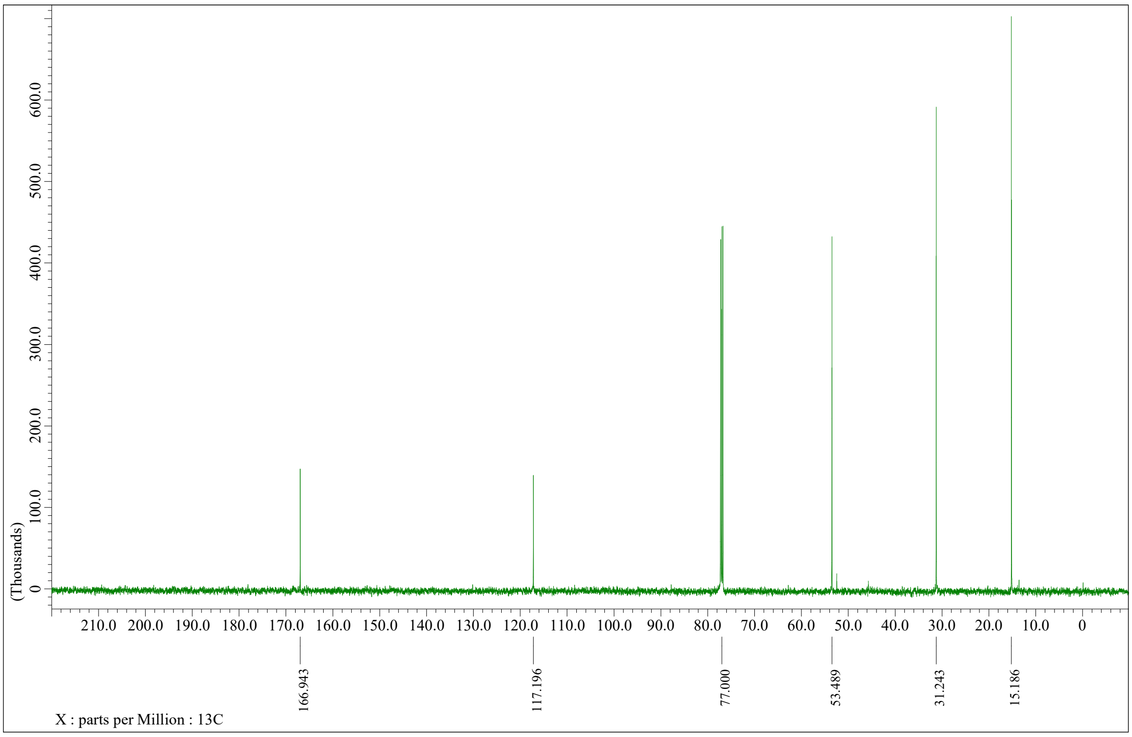
(Thousands)
600.0
500.0
400.0
300.0
200.0
100.0
0
210.0
200.0
190.0
180.0
170.0
160.0
150.0
140.0
130.0
120.0
110.0
100.0
90.0
80.0
70.0
60.0
50.0
40.0
30.0
20.0
10.0
0
166.943
117.196
77.000
53.489
31.243
15.186
X : parts per Million : 13C

▪^{1}H-NMR and HPLC charts of **17a** for **Scheme 1**.

Without Catalyst (65% yield, dr 28 : 72)

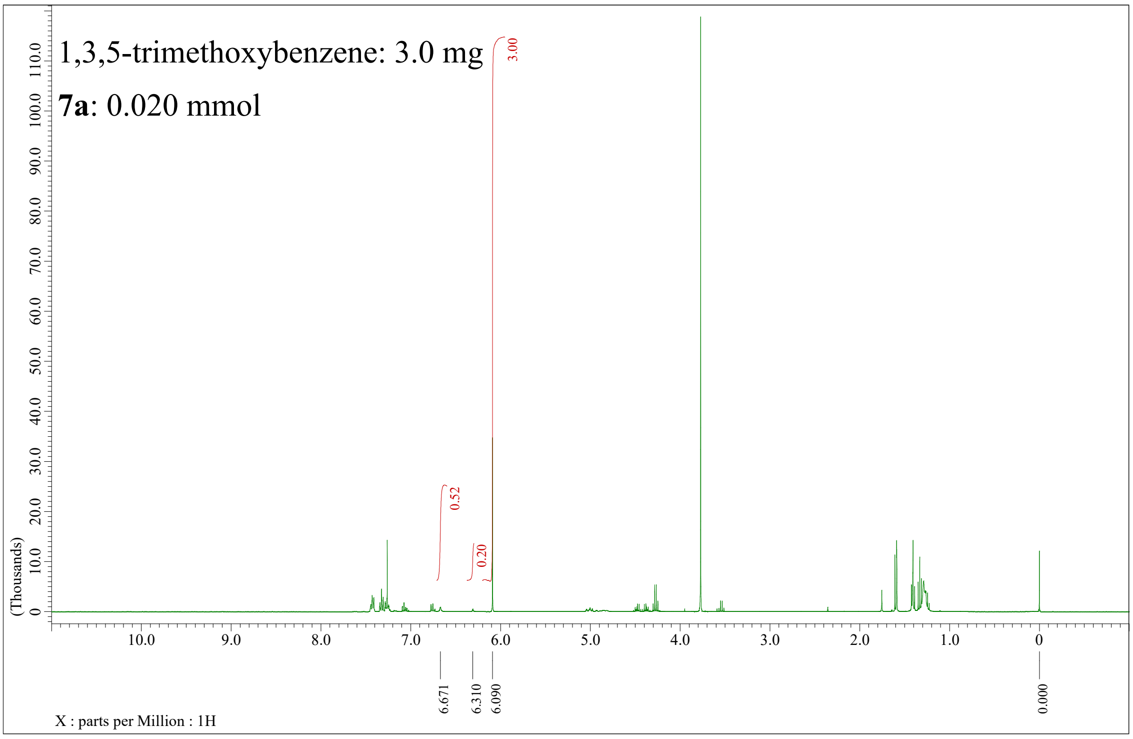

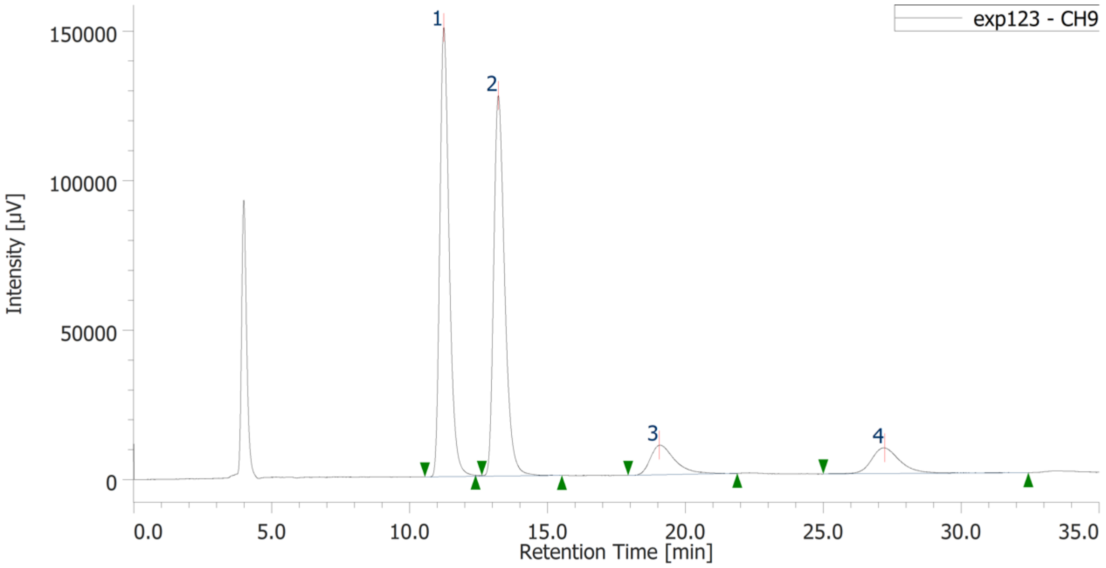

#	Peak Name	CH	tR [min]	Area [µV·sec]	Height [µV]	Area%	Height%
1	Unknown	9	11.237	3542218	150105	42.747	50.7
2	Unknown	9	13.213	3509767	127176	42.355	43.0
3	Unknown	9	19.053	617796	9904	7.455	3.35
4	Unknown	9	27.217	616677	8603	7.442	2.91

Catalyzed by **9a** (83% yield, dr 51 : 49)

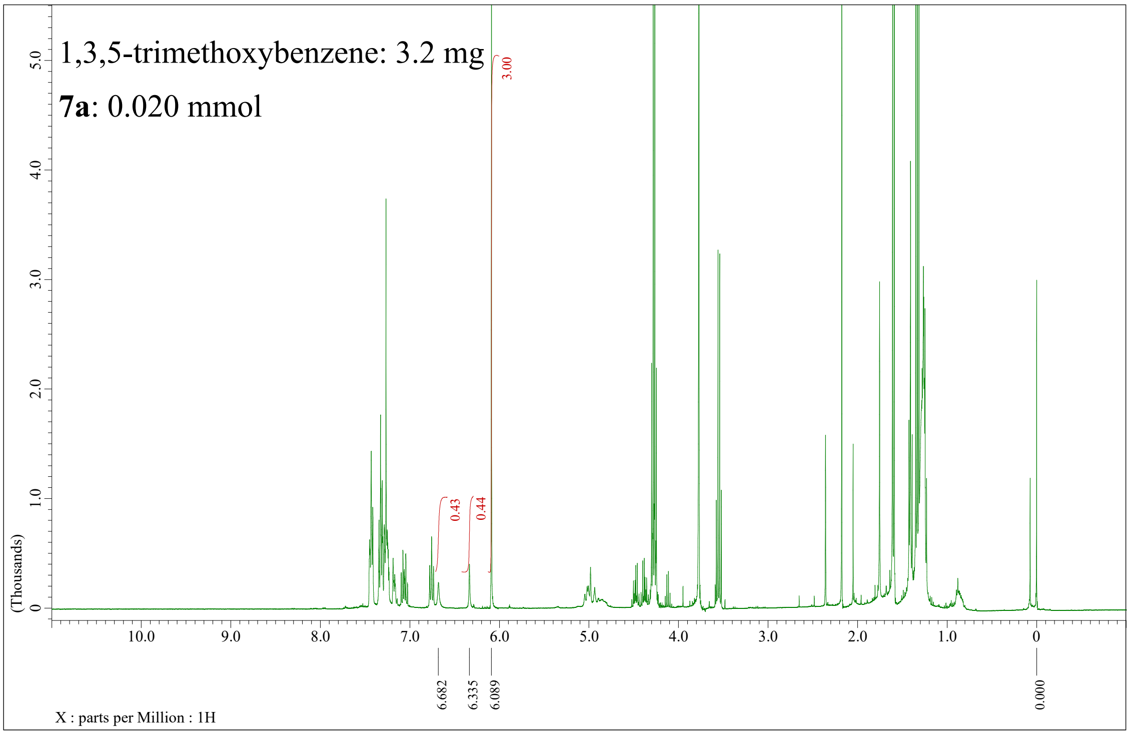

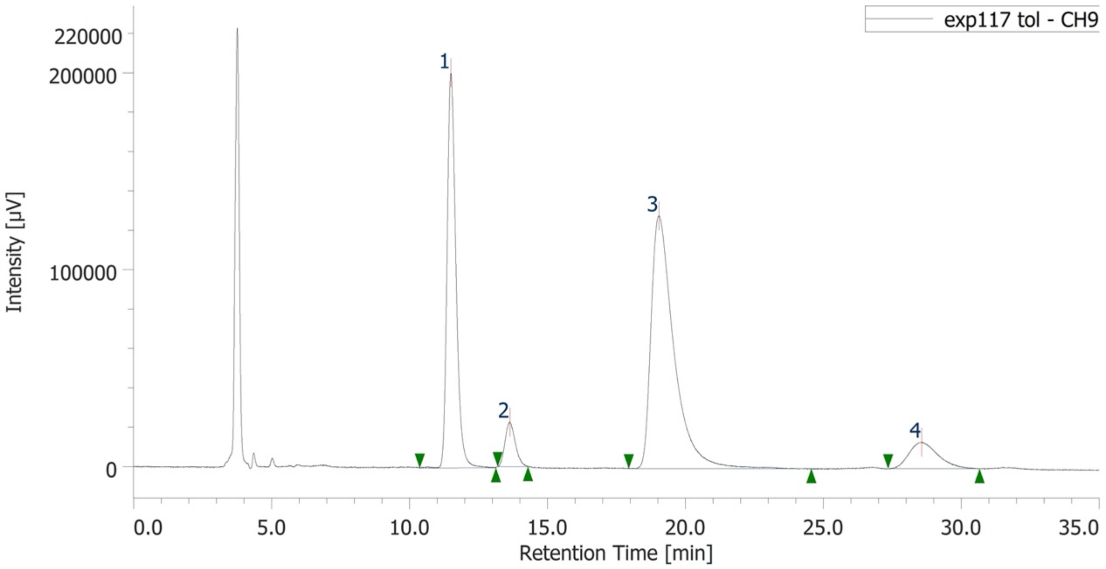

#	Peak Name	CH	tR [min]	Area [µV·sec]	Height [µV]	Area%	Height%
1	Unknown	9	11.49	4444070	200381	33.111	54.890
2	Unknown	9	13.63	620004	23006	4.619	6.302
3	Unknown	9	19.04	7377623	128250	54.967	35.131
4	Unknown	9	28.55	980218	13423	7.303	3.677

Catalyzed by **9b** (79% yield, dr 70 : 30)

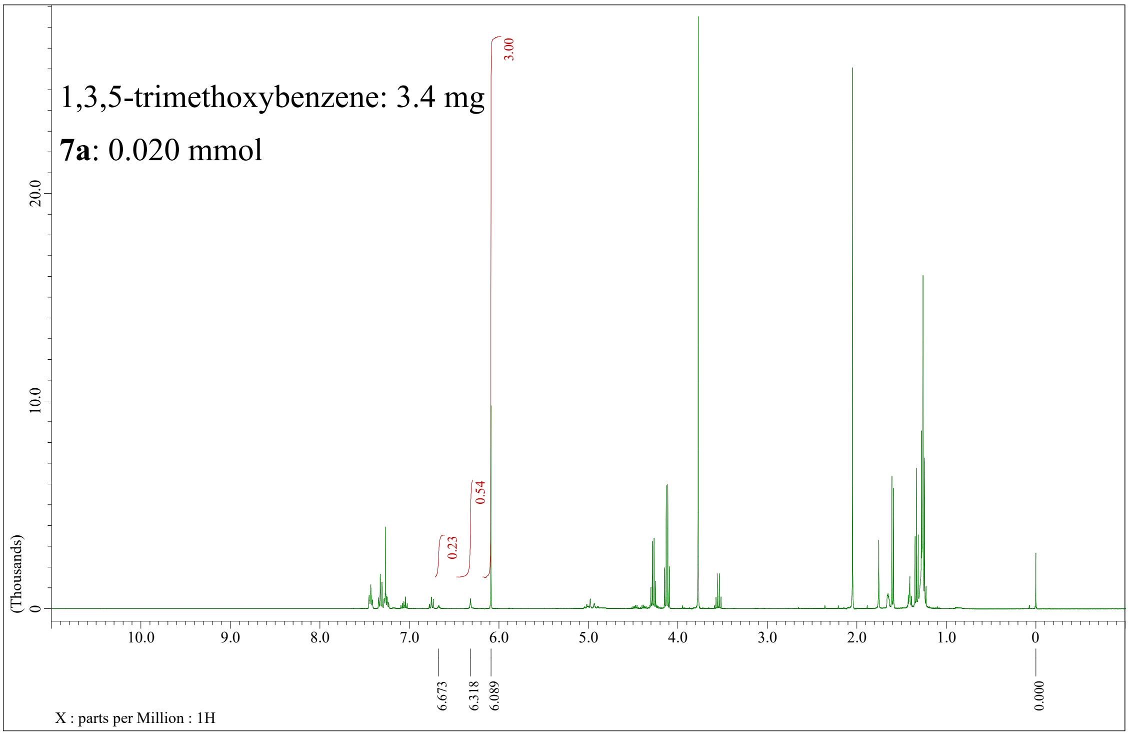

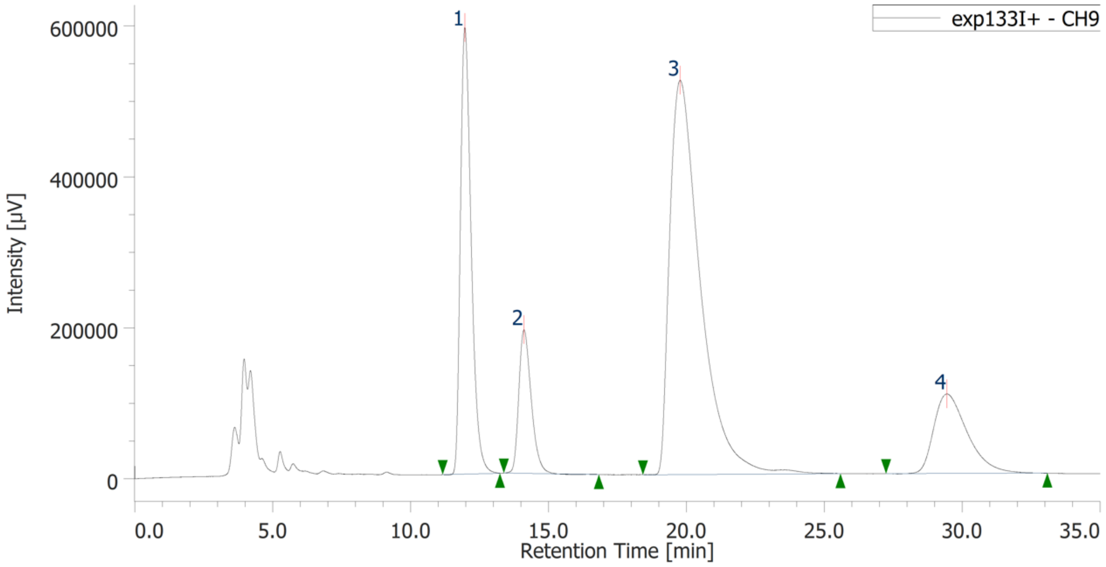

#	Peak Name	CH	tR [min]	Area [μV·sec]	Height [μV]	Area%	Height%
1	Unknown	9	11.965	16085241	591296	22.559	42.0
2	Unknown	9	14.107	5946023	190228	8.339	13.5
3	Unknown	9	19.770	40345132	521987	56.582	37.0
4	Unknown	9	29.437	8927122	105394	12.520	7.48

Catalyzed by **9c** (63% yield, dr 24 : 76)

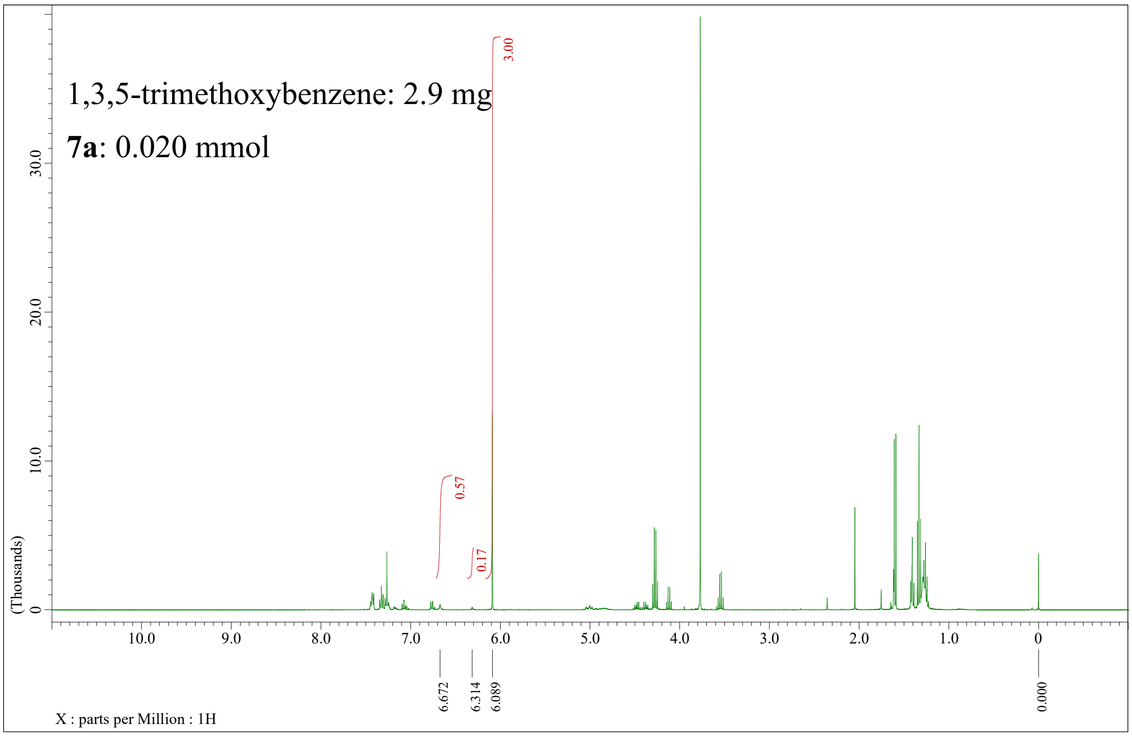

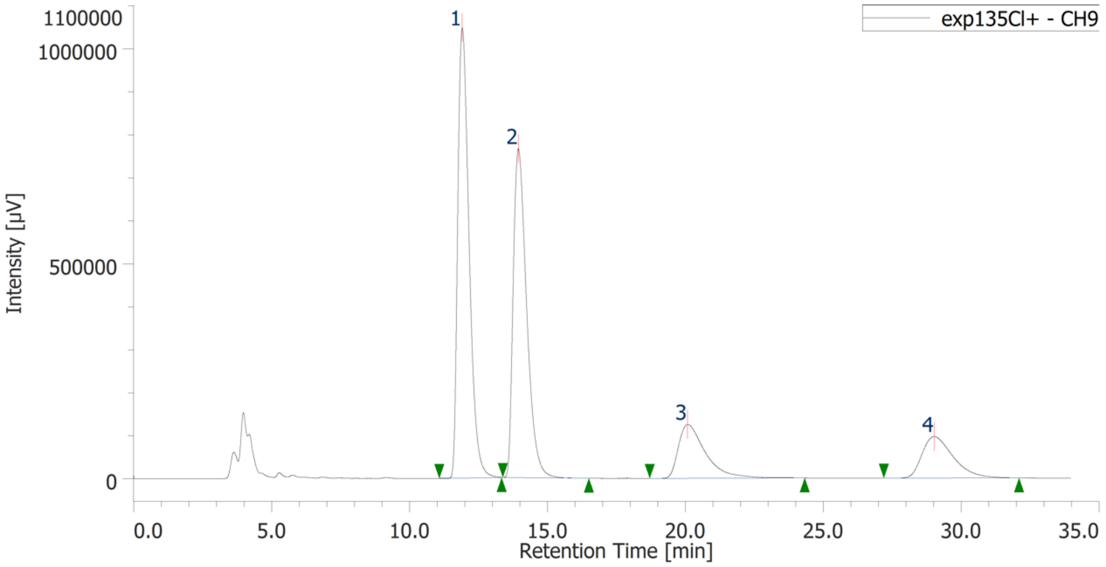

#	Peak Name	CH	tR [min]	Area [µV·sec]	Height [µV]	Area%	Height%
1	Unknown	9	11.903	29893775	1047237	41.616	51.5
2	Unknown	9	13.940	25843111	764695	35.977	37.6
3	Unknown	9	20.070	8526835	124908	11.871	6.14
4	Unknown	9	29.013	7567909	96585	10.536	4.75

^{1}H-NMR and HPLC charts of **17a** for **Table 1**.

Entry 1 (Toluene) (83% yield, dr 51 : 49)

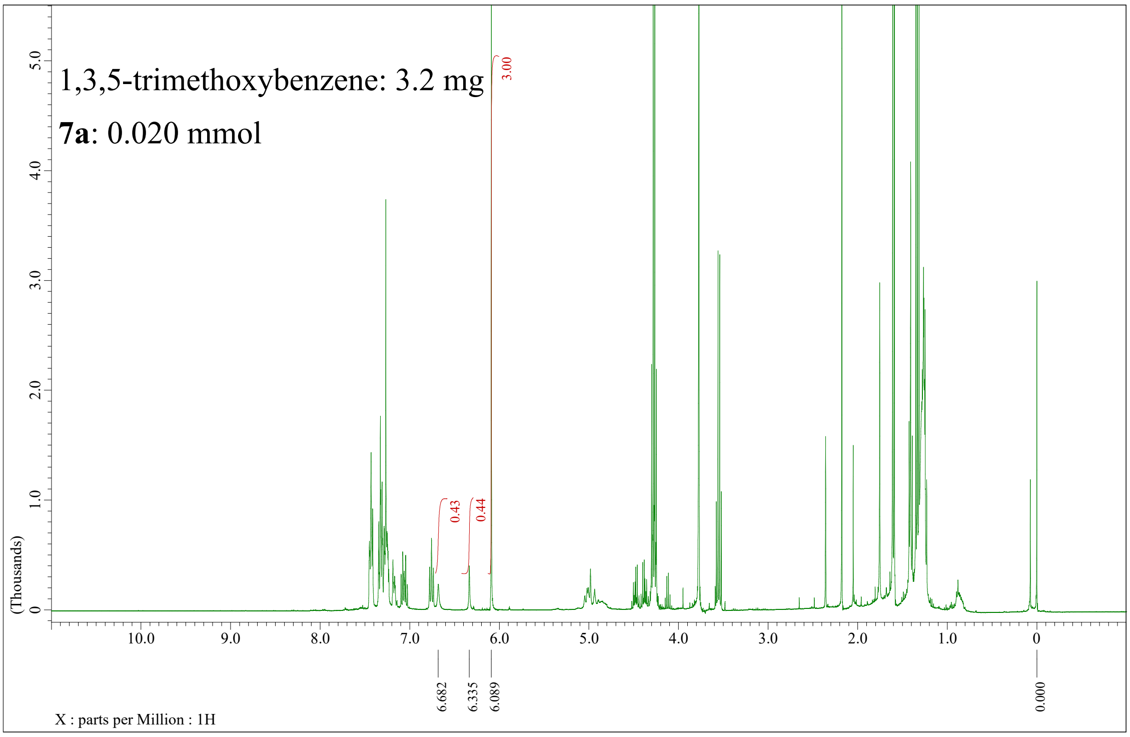

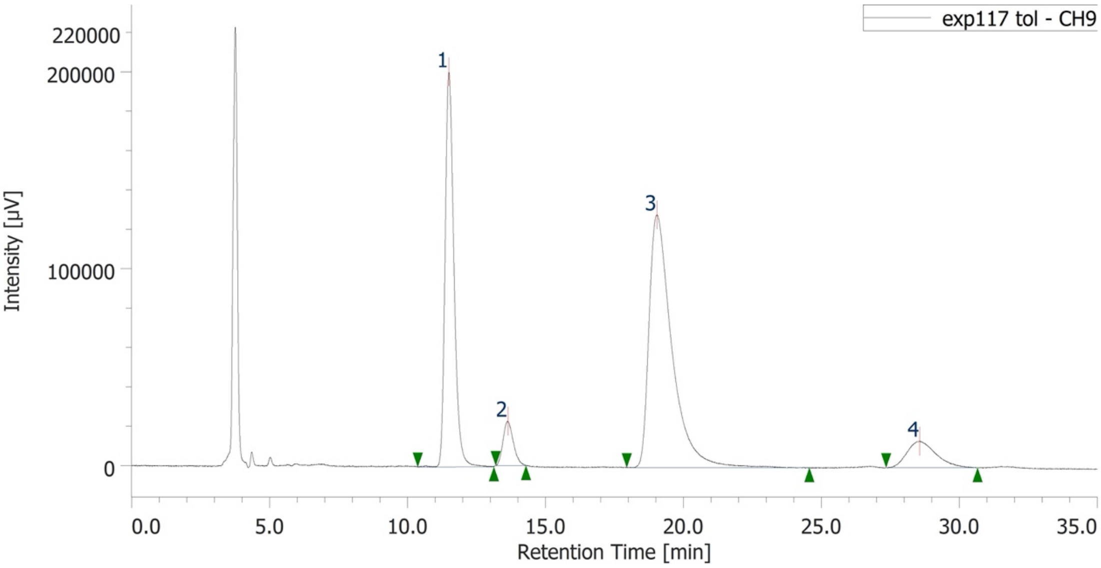

#	Peak Name	CH	tR [min]	Area [μV·sec]	Height [μV]	Area%	Height%
1	Unknown	9	11.49	4444070	200381	33.111	54.890
2	Unknown	9	13.63	620004	23006	4.619	6.302
3	Unknown	9	19.04	7377623	128250	54.967	35.131
4	Unknown	9	28.55	980218	13423	7.303	3.677

Entry 2 (Et_2O) (76% yield, dr 57 : 43)

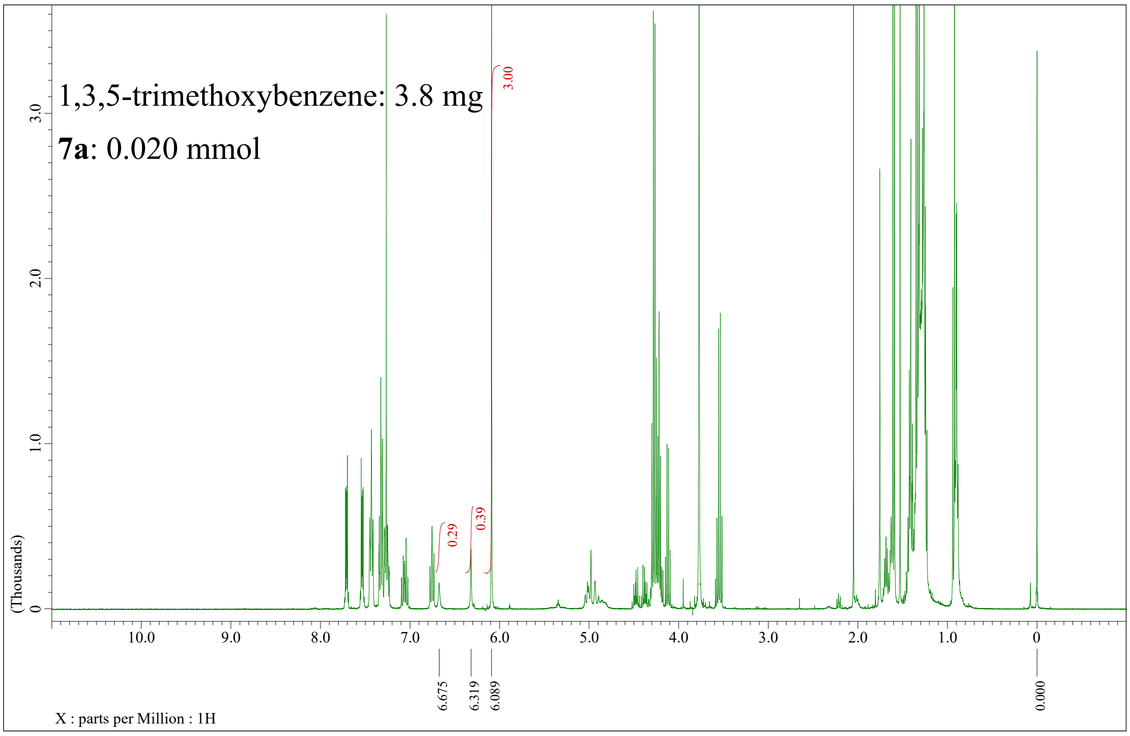

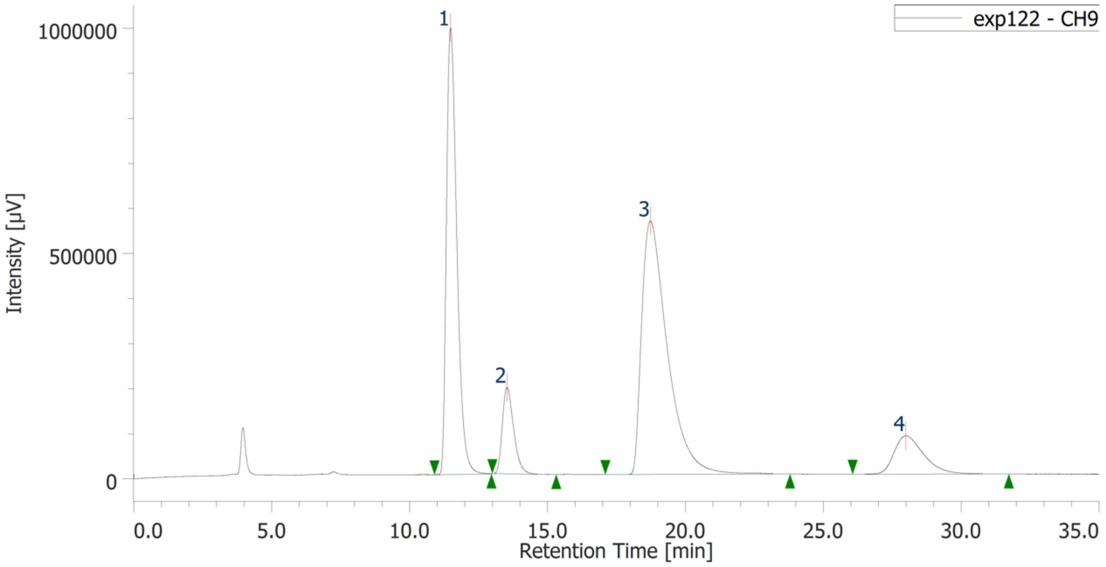

#	Peak Name	CH	tR [min]	Area [µV·sec]	Height [µV]	Area%	Height%
1	Unknown	9	11.480	25913687	991282	34.922	54.2
2	Unknown	9	13.527	5479073	192224	7.384	10.5
3	Unknown	9	18.728	36452785	562111	49.124	30.7
4	Unknown	9	27.985	6359890	84995	8.571	4.64

Entry 3 (CH_2Cl_2) (76% yield, dr 58 : 42)

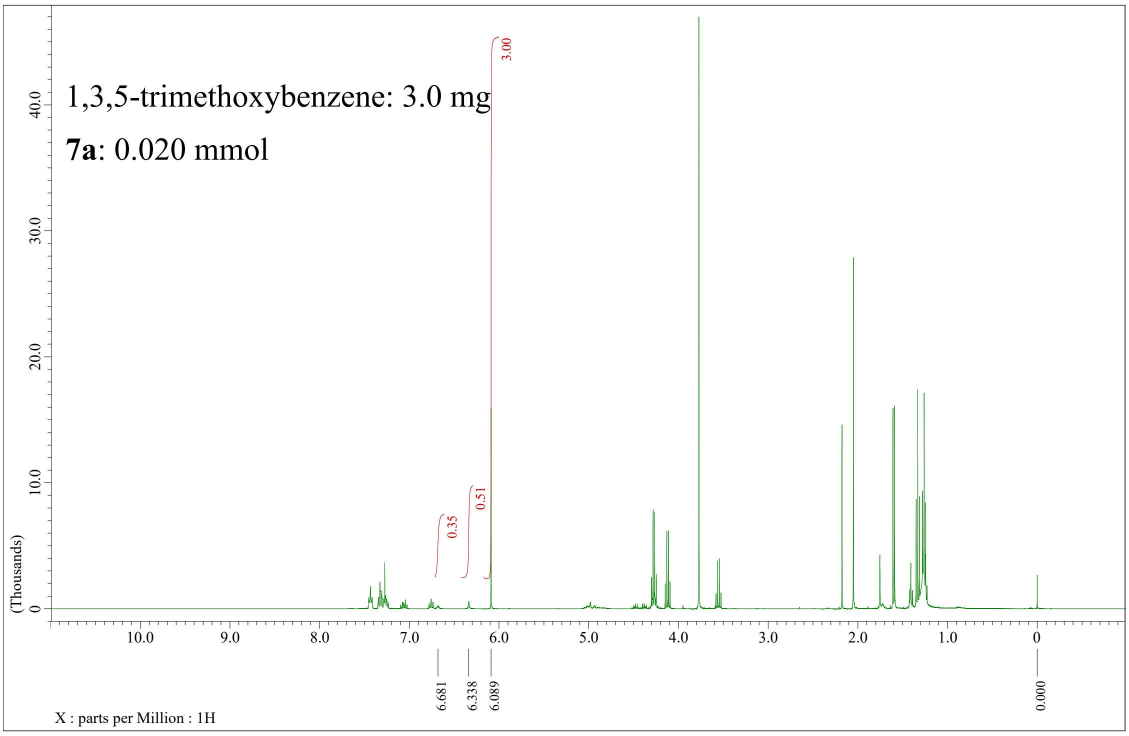

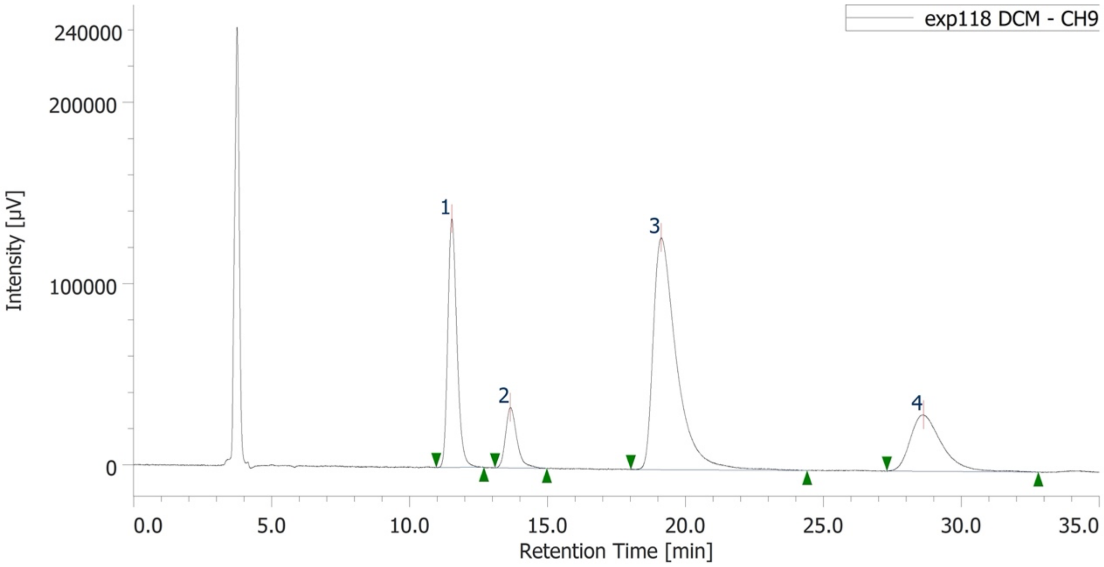

#	Peak Name	CH	tR [min]	Area [µV·sec]	Height [µV]	Area%	Height%
1	Unknown	9	11.53	3132883	137068	22.220	41.598
2	Unknown	9	13.65	950701	33354	6.743	10.122
3	Unknown	9	19.12	7538941	127910	53.470	38.819
4	Unknown	9	28.62	2476931	31175	17.568	9.461

Entry 4 (THF) (77% yield, dr 58 : 42)

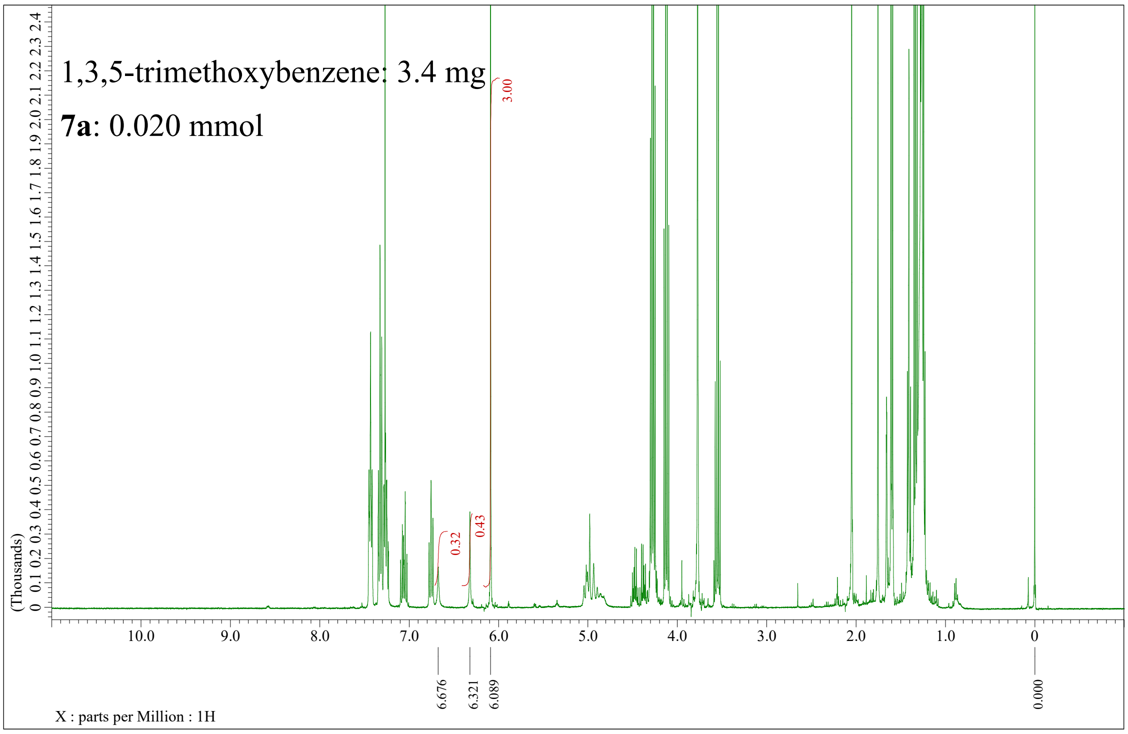

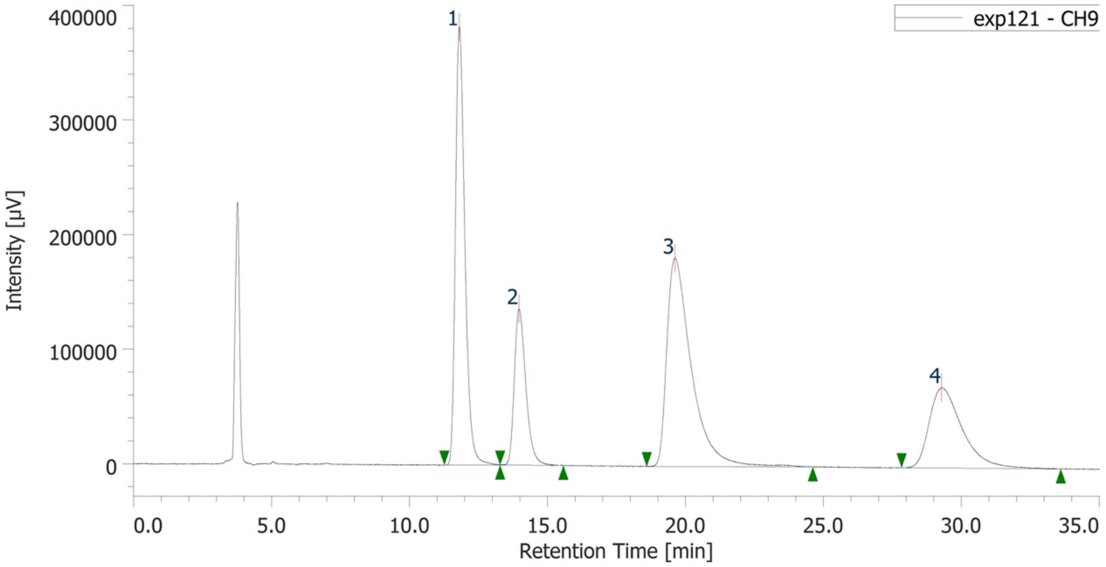

#	Peak Name	CH	tR [min]	Area [μV·sec]	Height [μV]	Area%	Height%
1	Unknown	9	11.80	9068487	381929	30.134	49.599
2	Unknown	9	13.96	3941462	136335	13.097	17.705
3	Unknown	9	19.61	11264992	181751	37.432	23.603
4	Unknown	9	29.27	5819384	70018	19.337	9.093

Entry 5 ($CHCl_3$) (68% yield, dr 60 : 40)

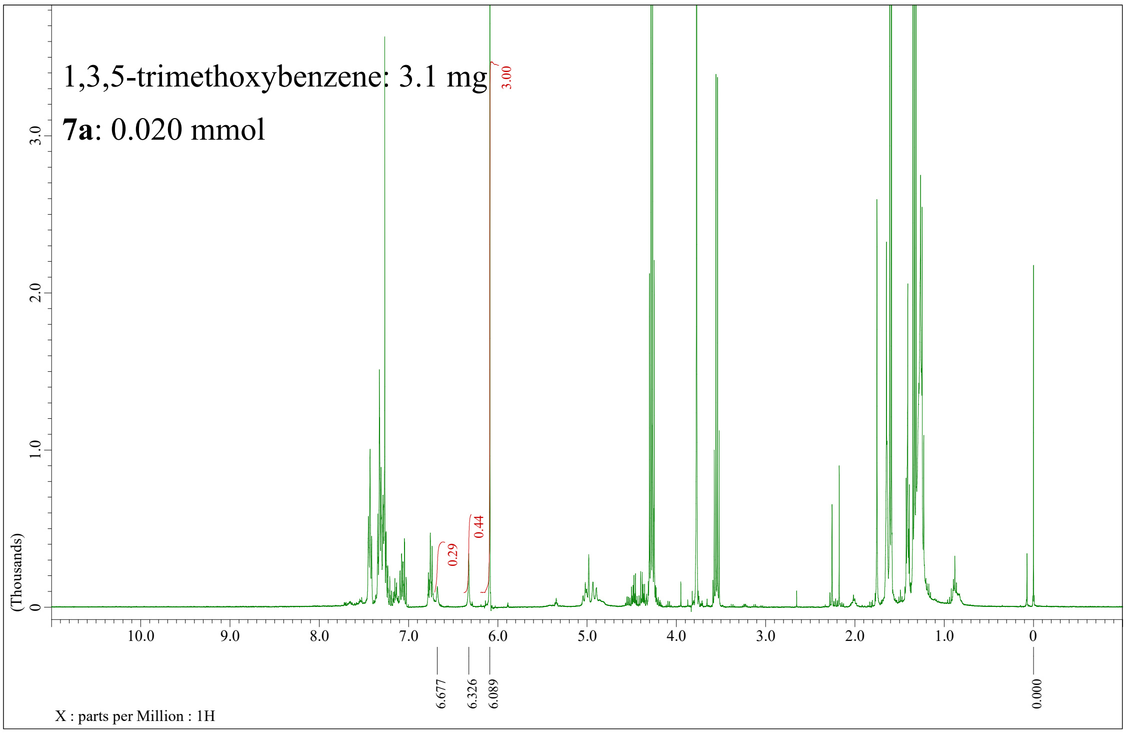

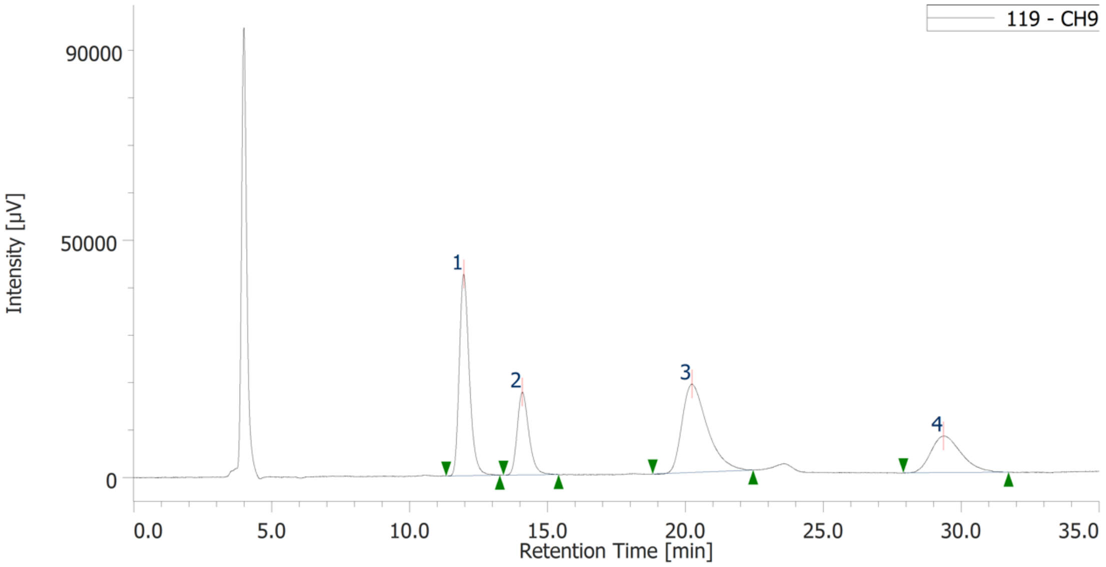

#	Peak Name	CH	tR [min]	Area [µV·sec]	Height [µV]	Area%	Height%
1	Unknown	9	11.960	1066406	42423	31.859	49.2
2	Unknown	9	14.083	510216	17414	15.243	20.2
3	Unknown	9	20.230	1182729	18626	35.334	21.6
4	Unknown	9	29.350	587903	7703	17.564	8.94

Entry 6 (CH_3CN) (90% yield, dr 70 : 30)

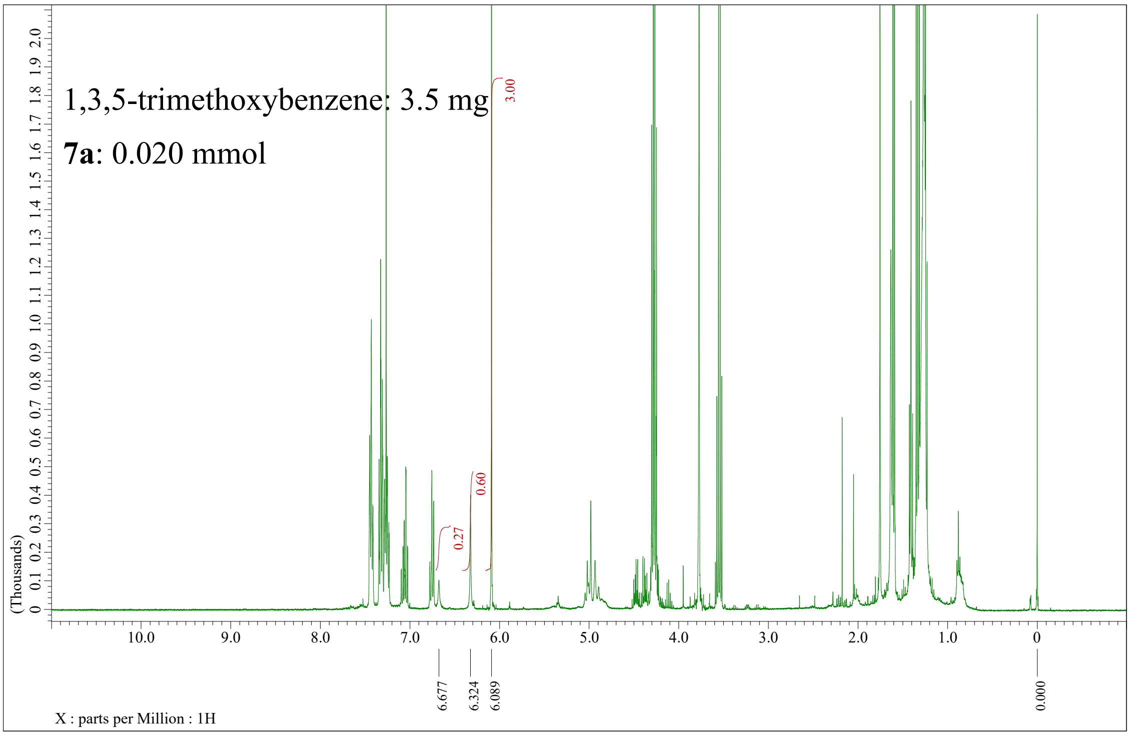

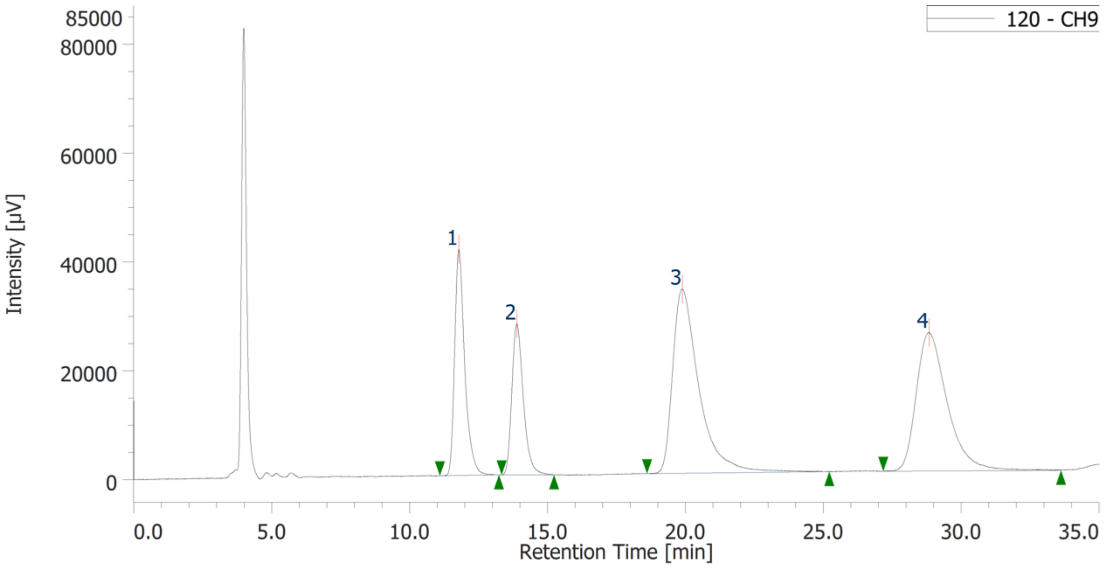

#	Peak Name	CH	tR [min]	Area [μV·sec]	Height [μV]	Area%	Height%
1	Unknown	9	11.780	1057801	41531	17.365	32.3
2	Unknown	9	13.883	803230	27718	13.186	21.6
3	Unknown	9	19.883	2245554	33828	36.863	26.3
4	Unknown	9	28.823	1985005	25436	32.586	19.8

Entry 7 (0 °C) (84% yield, dr 25 : 75)

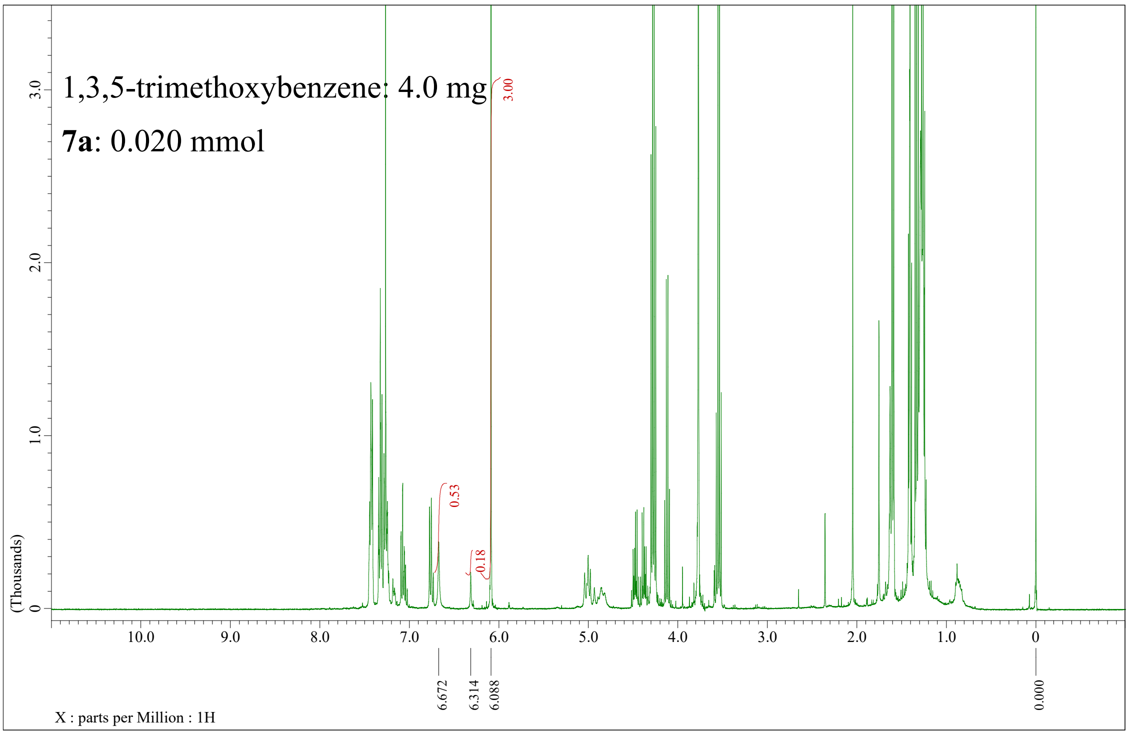

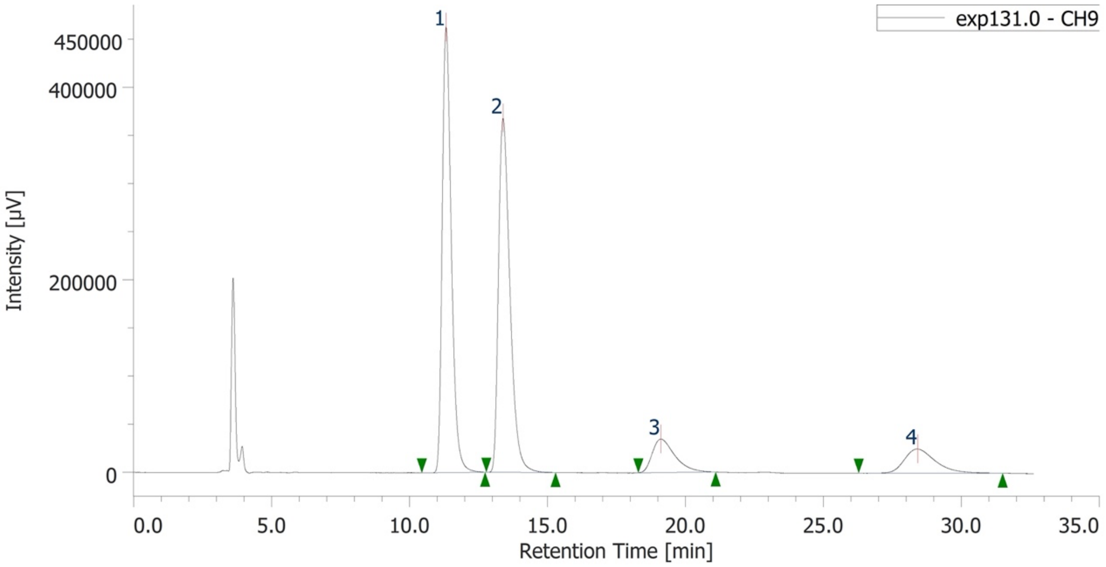

#	Peak Name	CH	tR [min]	Area [µV·sec]	Height [µV]	Area%	Height%
1	Unknown	9	11.32	10636192	462569	42.226	51.959
2	Unknown	9	13.38	10597306	367755	42.071	41.308
3	Unknown	9	19.11	1986256	34808	7.885	3.910
4	Unknown	9	28.42	1969145	25134	7.818	2.823

Entry 8 (−20 °C) (90% yield, dr 52 : 48)

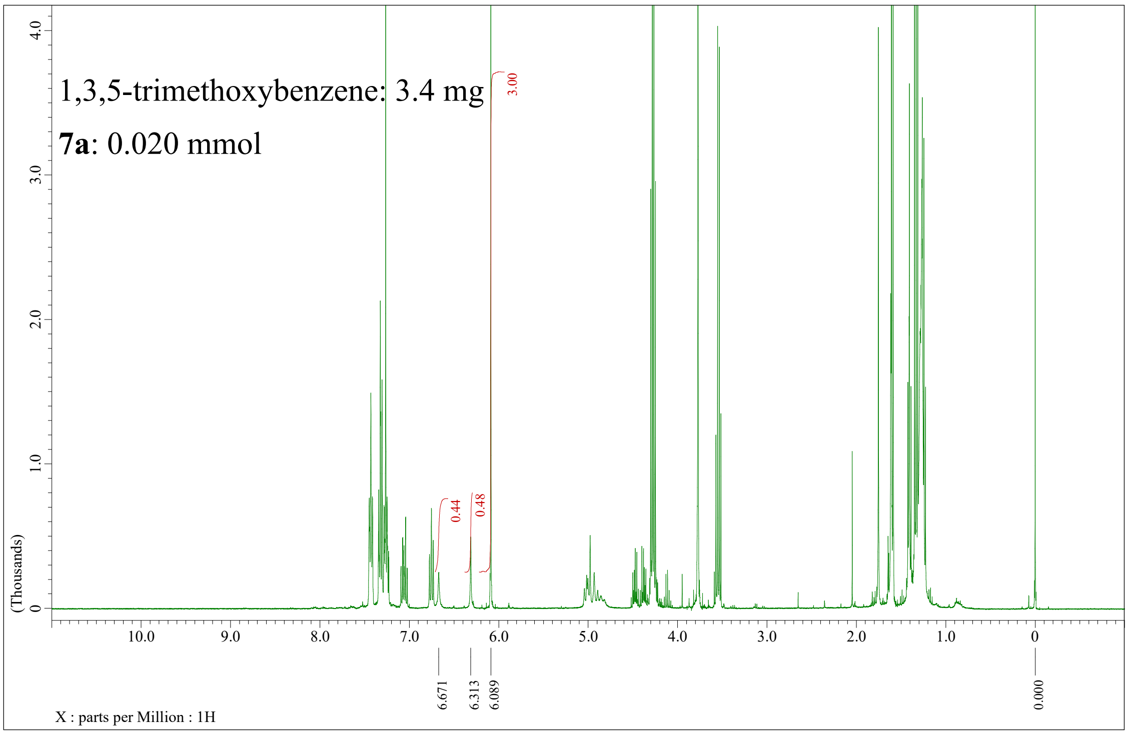

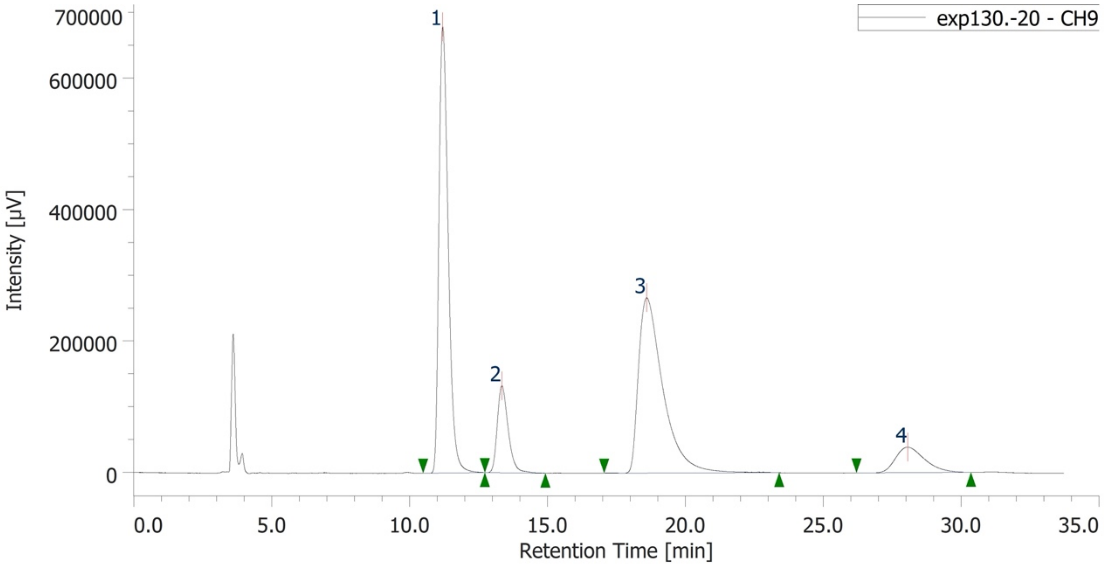

#	Peak Name	CH	tR [min]	Area [µV·sec]	Height [µV]	Area%	Height%
1	Unknown	9	11.20	15974252	678798	41.042	60.799
2	Unknown	9	13.34	3624133	132198	9.311	11.841
3	Unknown	9	18.59	16466733	266607	42.307	23.880
4	Unknown	9	28.05	2856681	38866	7.340	3.481

Entry 9 (−80 °C, 96 h) (57% yield, dr 40 : 60)

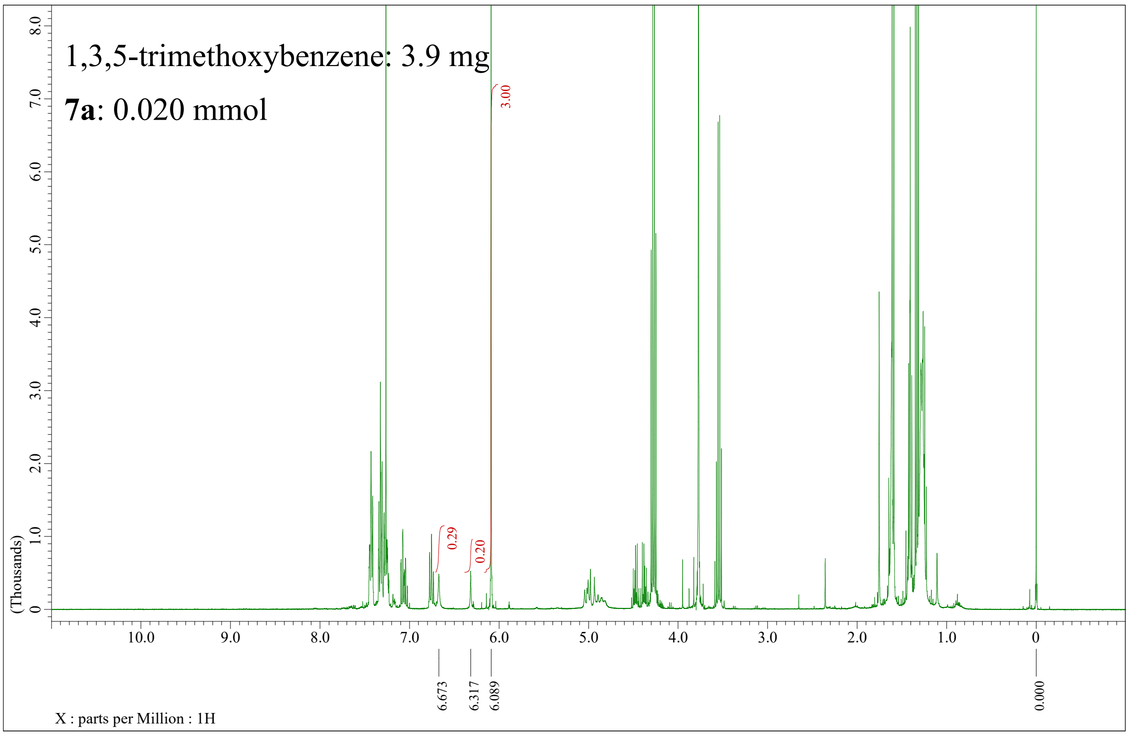

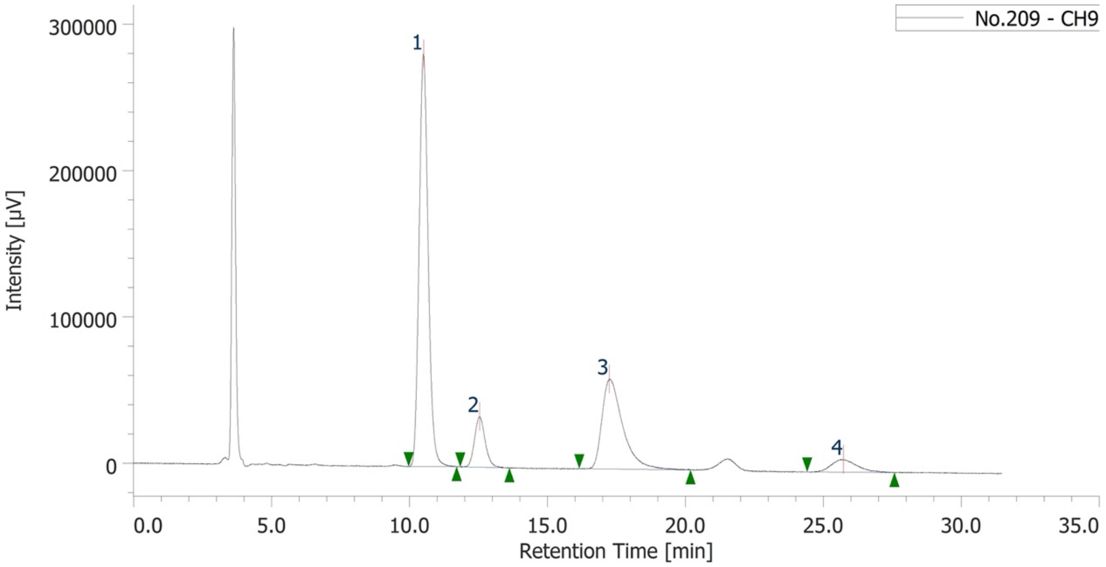

#	Peak Name	CH	tR [min]	Area [µV·sec]	Height [µV]	Area%	Height%
1	Unknown	9	10.50	6424854	281666	57.867	72.928
2	Unknown	9	12.54	923493	34466	8.318	8.924
3	Unknown	9	17.23	3199476	61394	28.817	15.896
4	Unknown	9	25.73	555040	8699	4.999	2.252

Entry 10 (10 mol% of K_2CO_3) (87% yield, dr 51 : 49)

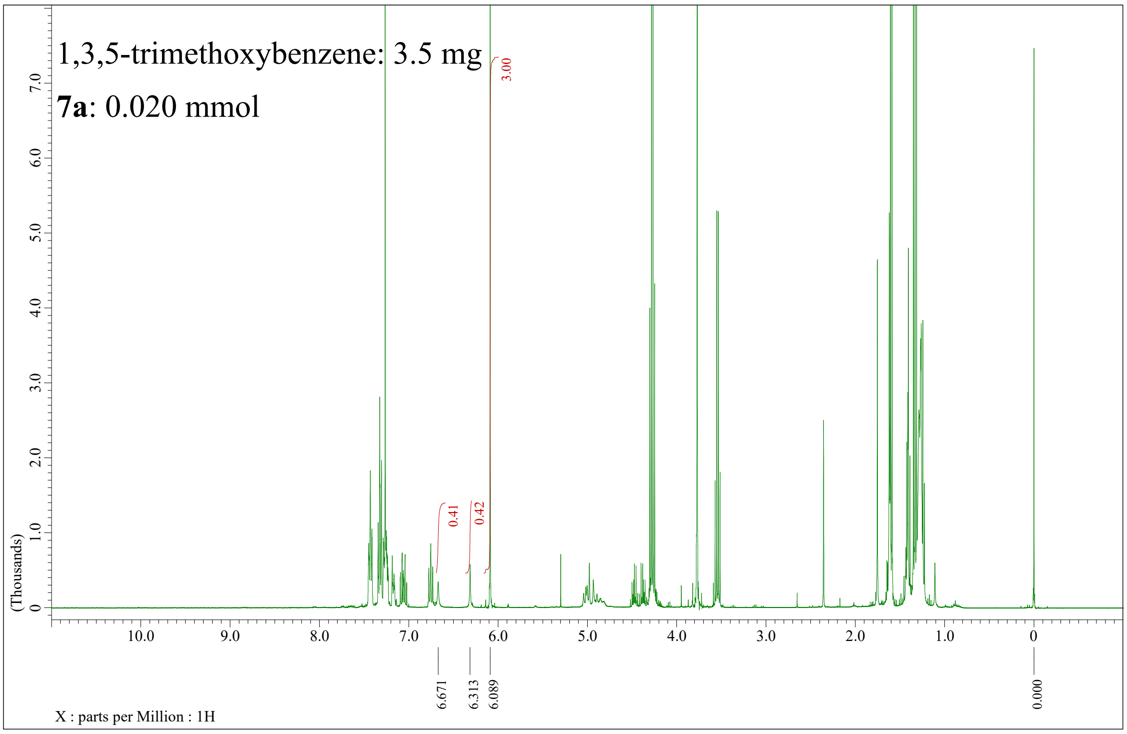

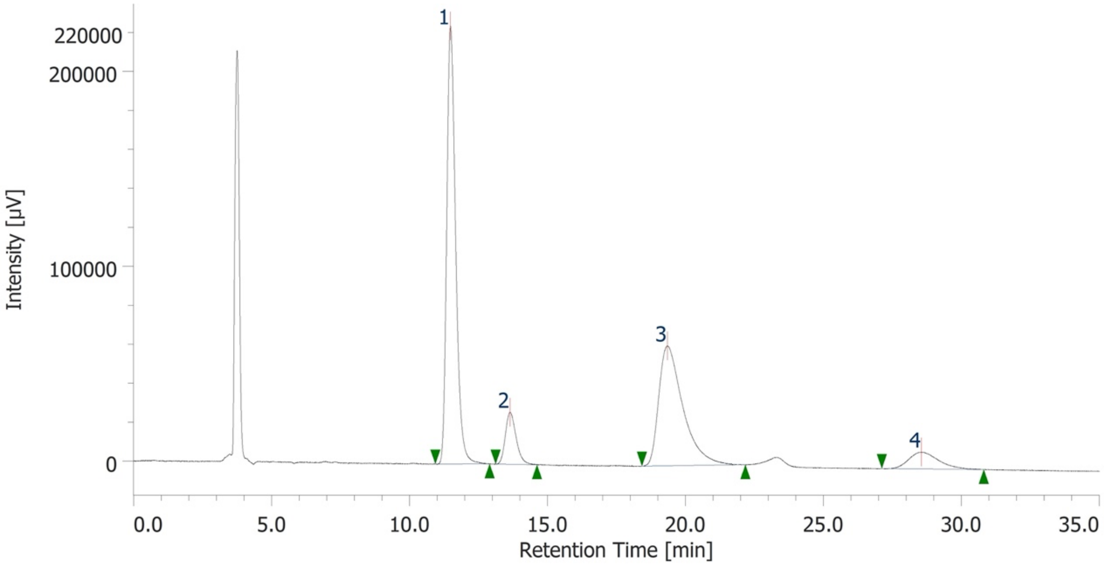

#	Peak Name	CH	tR [min]	Area [µV·sec]	Height [µV]	Area%	Height%
1	Unknown	9	11.48	5040019	224592	49.662	69.900
2	Unknown	9	13.64	716178	26638	7.057	8.290
3	Unknown	9	19.34	3741796	61449	36.870	19.125
4	Unknown	9	28.55	650717	8625	6.412	2.684

Entry 11 (5 mol% of **9a**) (82% yield, dr 51 : 49)

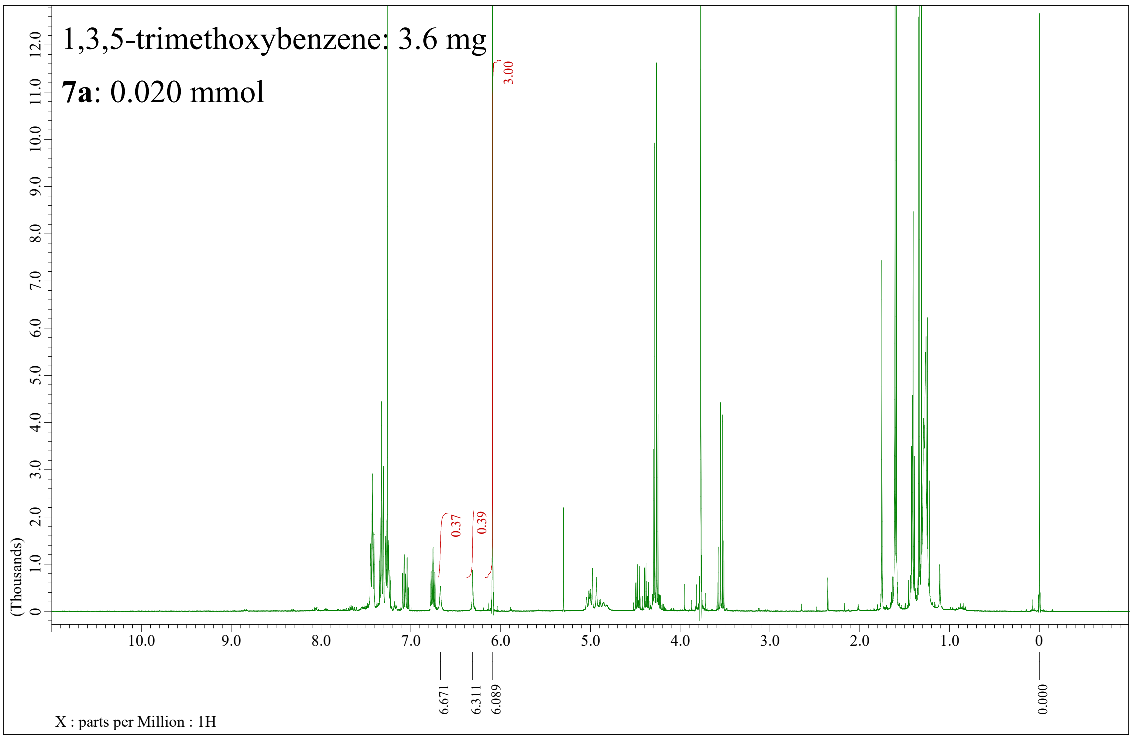

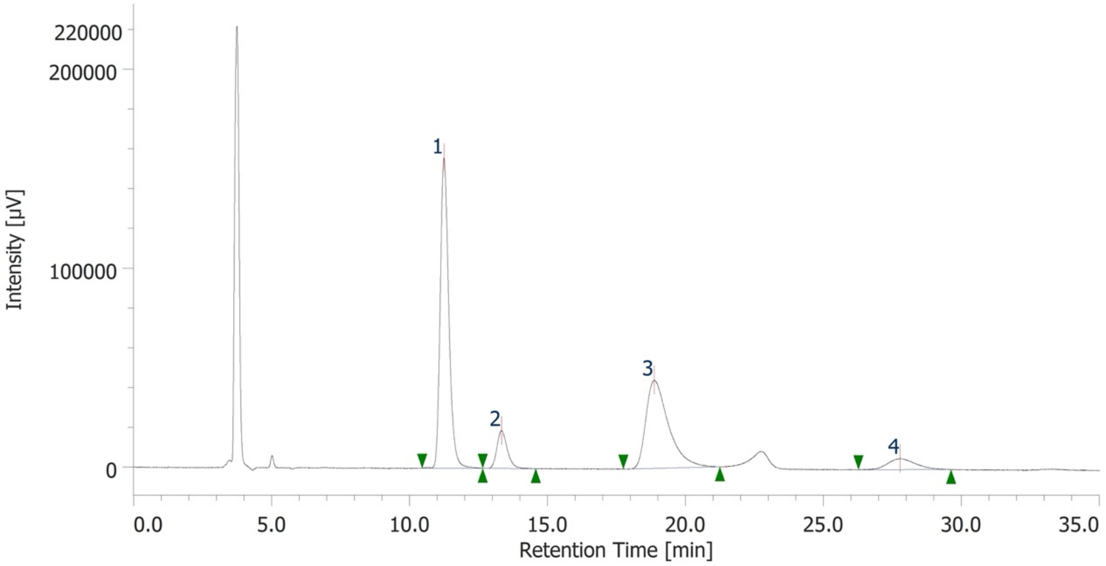

#	Peak Name	CH	tR [min]	Area [µV·sec]	Height [µV]	Area%	Height%
1	Unknown	9	11.24	3349355	155839	49.683	69.357
2	Unknown	9	13.33	493124	19010	7.315	8.460
3	Unknown	9	18.86	2510137	44278	37.234	19.706
4	Unknown	9	27.78	388875	5565	5.768	2.477

Entry 12 (1.5 equiv of **16a**) (68% yield, dr 54 : 46)

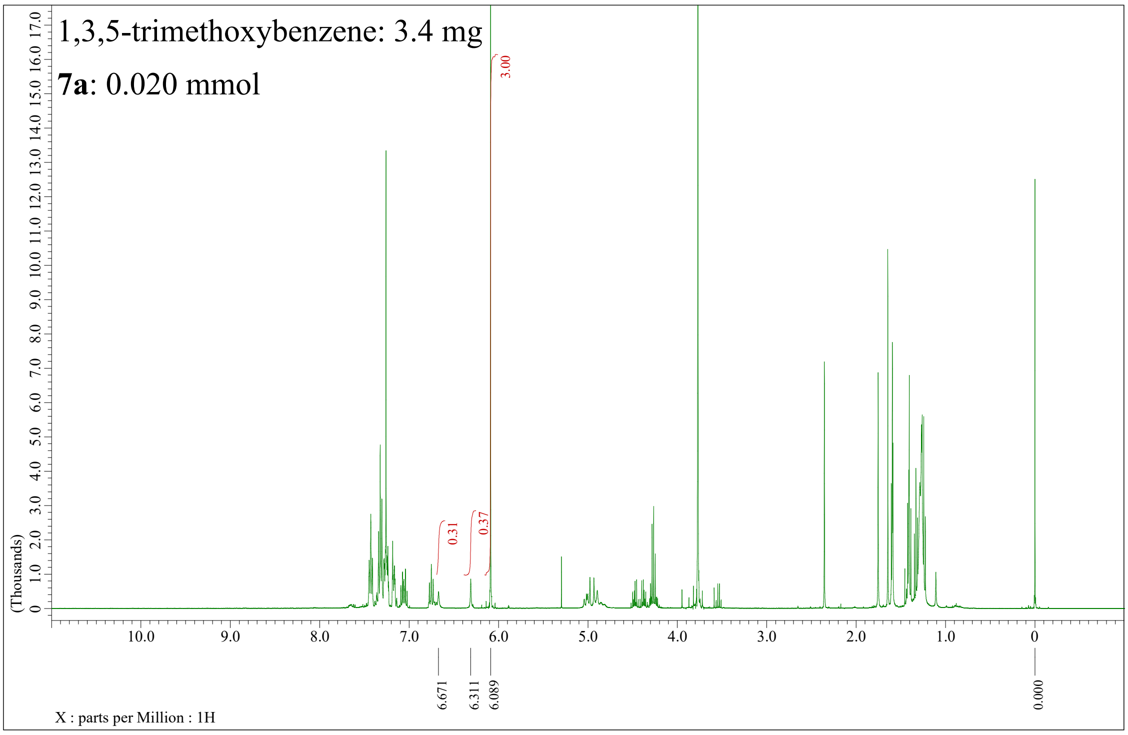

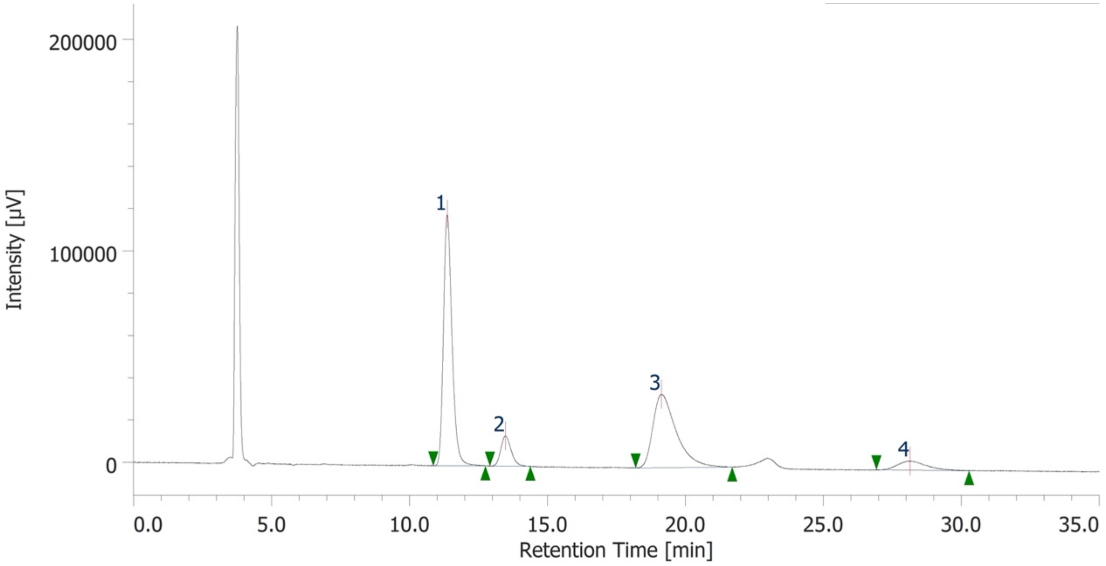

#	Peak Name	CH	tR [min]	Area [µV·sec]	Height [µV]	Area%	Height%
1	Unknown	9	11.36	2565657	118801	48.524	69.080
2	Unknown	9	13.47	373059	14256	7.056	8.290
3	Unknown	9	19.12	2039123	34595	38.565	20.116
4	Unknown	9	28.13	309614	4323	5.856	2.514

Entry 13 (Toluene 0.1M) (74% yield, dr 50 : 50)

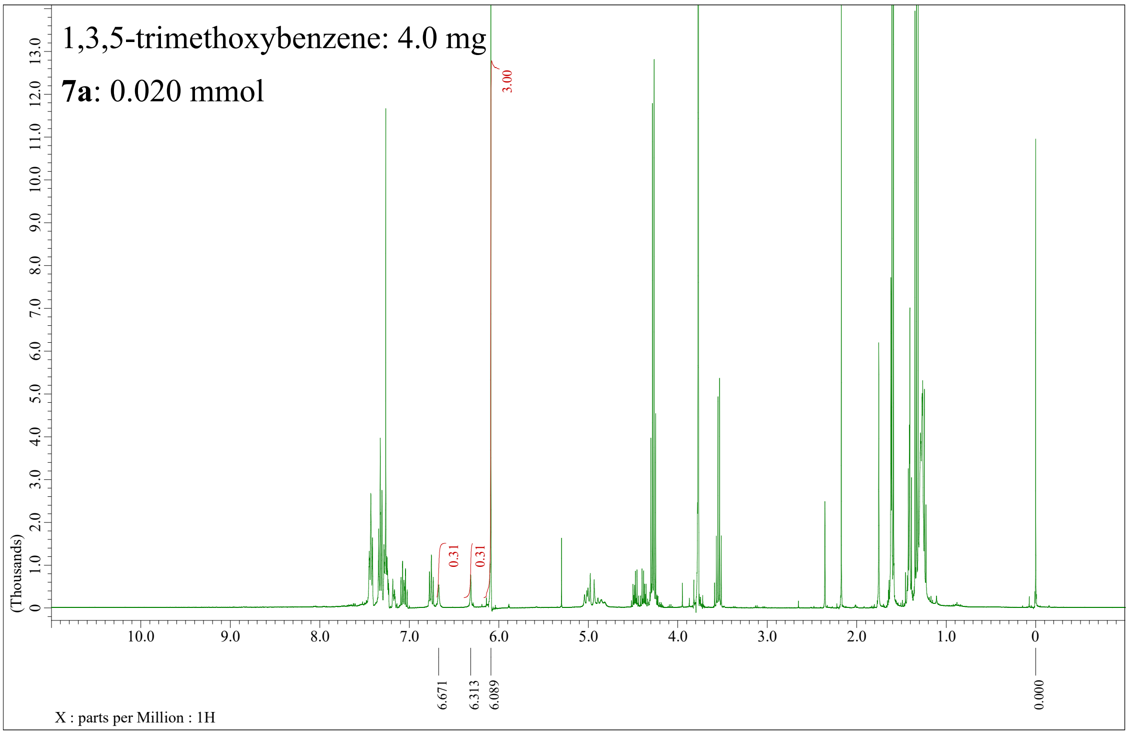

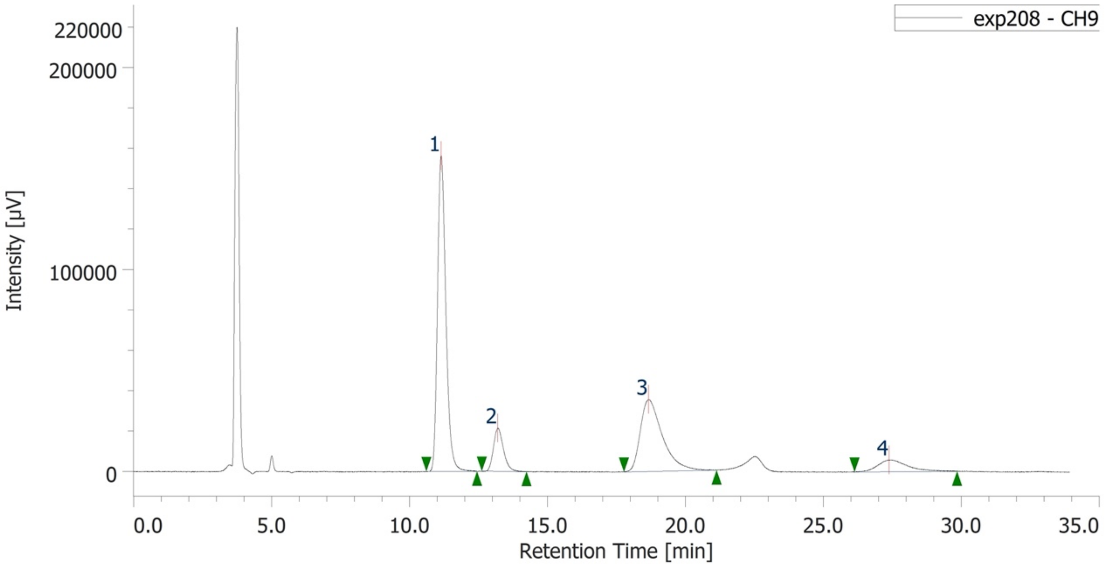

#	Peak Name	CH	tR [min]	Area [µV·sec]	Height [µV]	Area%	Height%
1	Unknown	9	11.14	3271232	156218	52.629	71.327
2	Unknown	9	13.19	534973	21352	8.607	9.749
3	Unknown	9	18.66	1969618	35581	31.688	16.246
4	Unknown	9	27.37	439790	5866	7.076	2.678

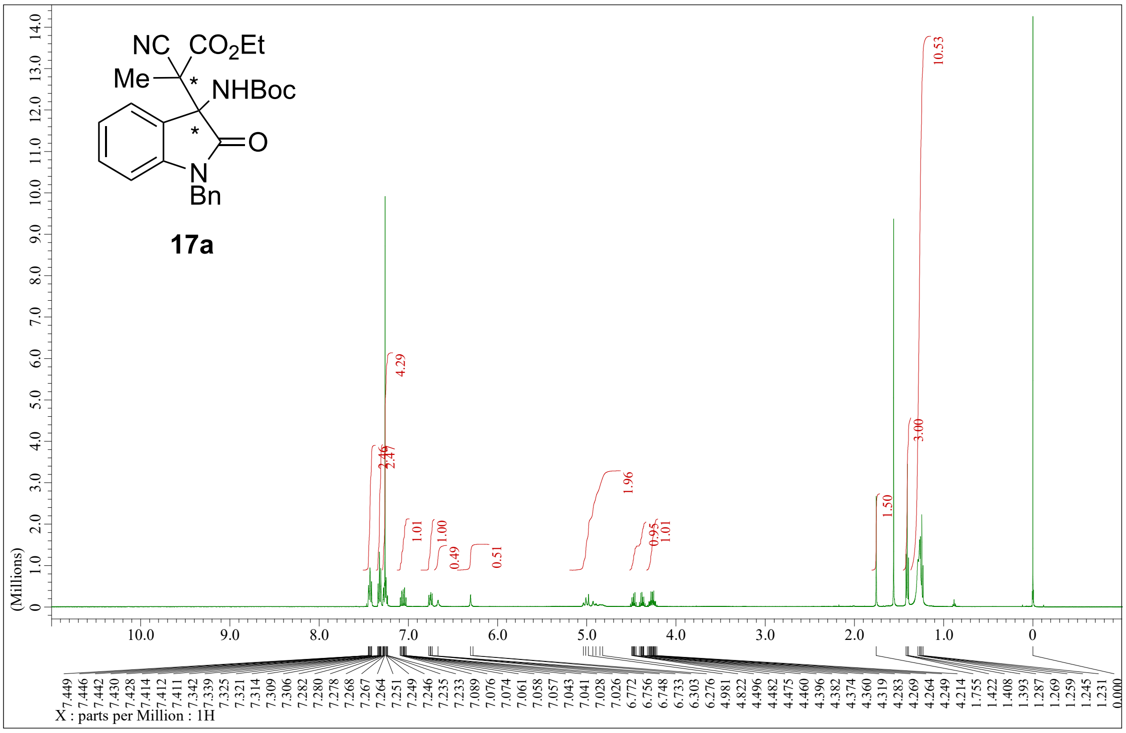

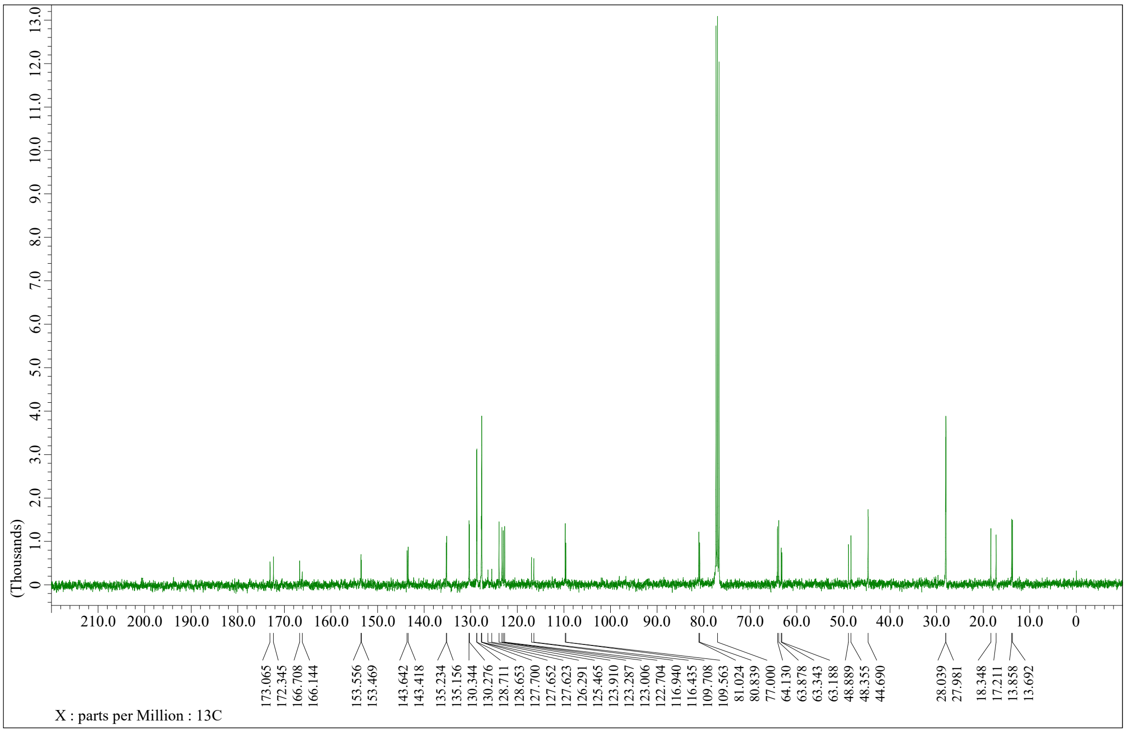

Racemic sample of **17a**

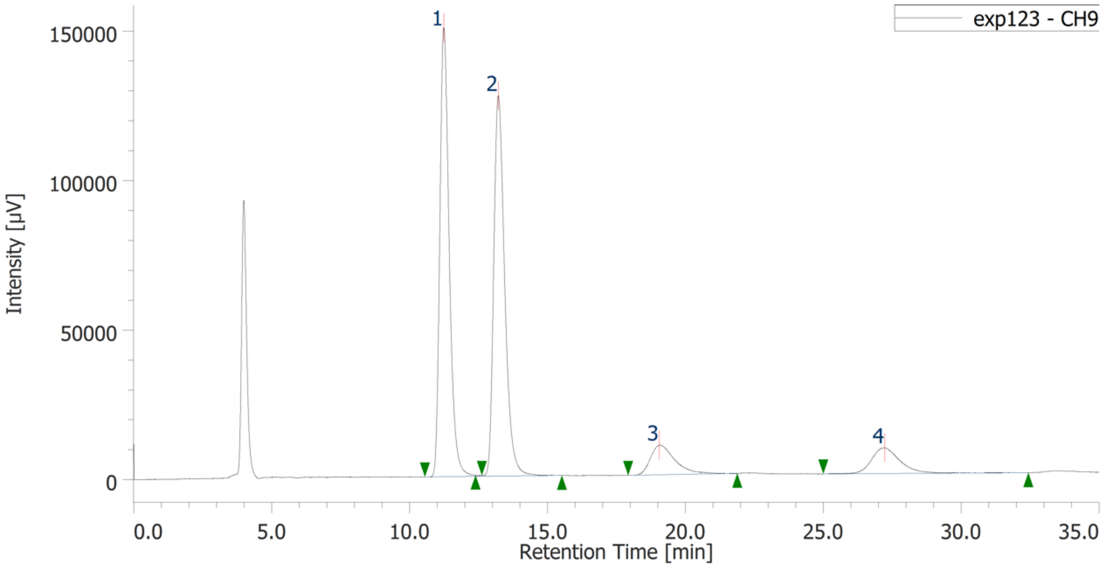

#	Peak Name	CH	tR [min]	Area [μV·sec]	Height [μV]	Area%	Height%
1	Unknown	9	11.237	3542218	150105	42.747	50.7
2	Unknown	9	13.213	3509767	127176	42.355	43.0
3	Unknown	9	19.053	617796	9904	7.455	3.35
4	Unknown	9	27.217	616677	8603	7.442	2.91

17a catalyzed by **9a**

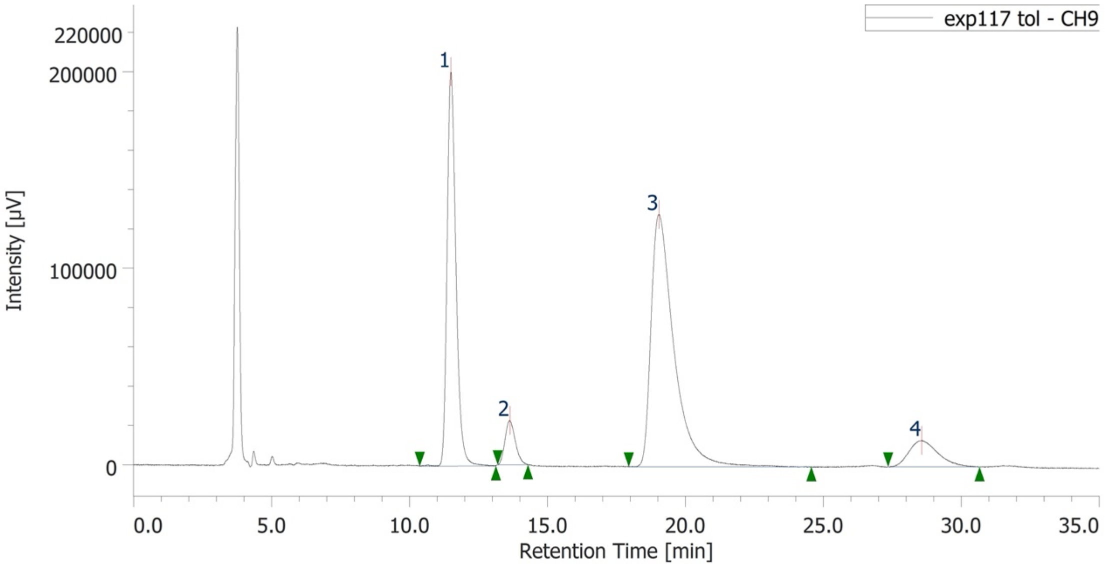

#	Peak Name	CH	tR [min]	Area [μV·sec]	Height [μV]	Area%	Height%
1	Unknown	9	11.49	4444070	200381	33.111	54.890
2	Unknown	9	13.63	620004	23006	4.619	6.302
3	Unknown	9	19.04	7377623	128250	54.967	35.131
4	Unknown	9	28.55	980218	13423	7.303	3.677

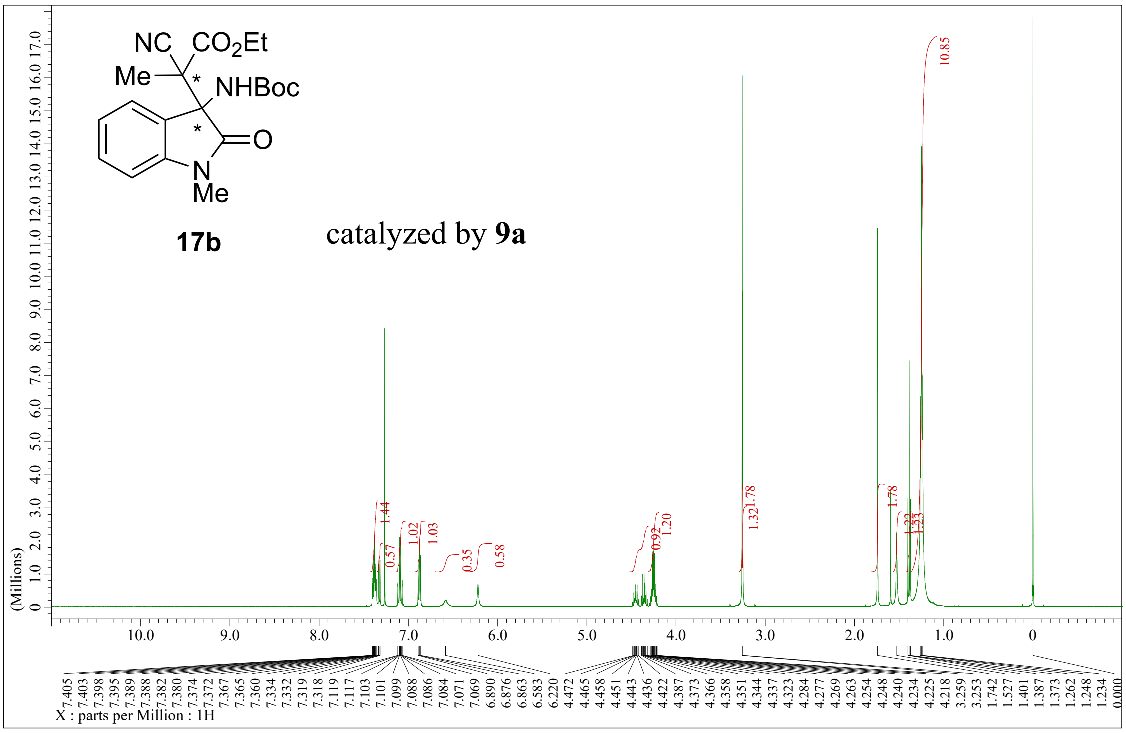
17b
catalyzed by 9a
X : parts per Million : 1H

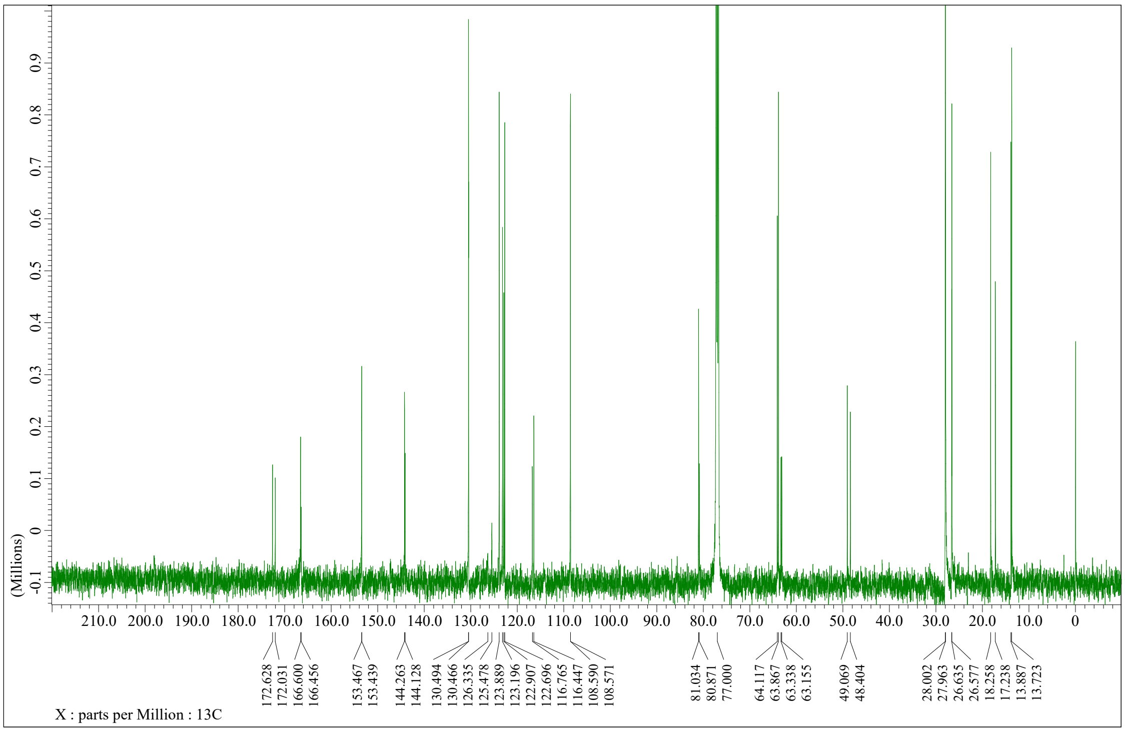
X : parts per Million : 13C

Racemic sample of **17b**

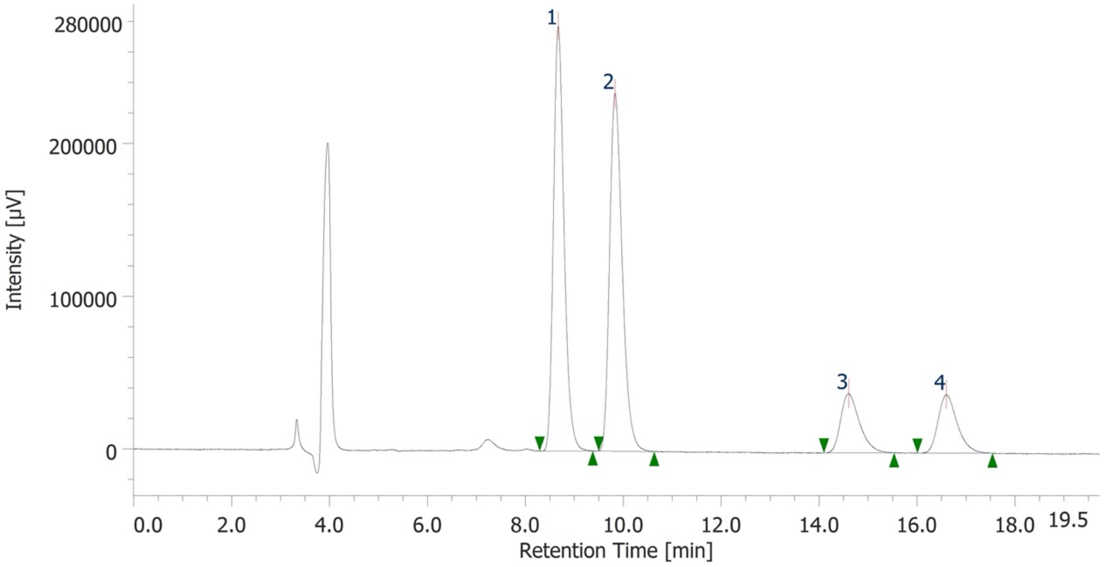

#	Peak Name	CH	tR [min]	Area [μV·sec]	Height [μV]	Area%	Height%
1	Unknown	9	8.67	4178901	278131	40.051	47.190
2	Unknown	9	9.83	4190131	234158	40.159	39.729
3	Unknown	9	14.60	1033791	38783	9.908	6.580
4	Unknown	9	16.59	1031144	38311	9.883	6.500

17b catalyzed by **9a**

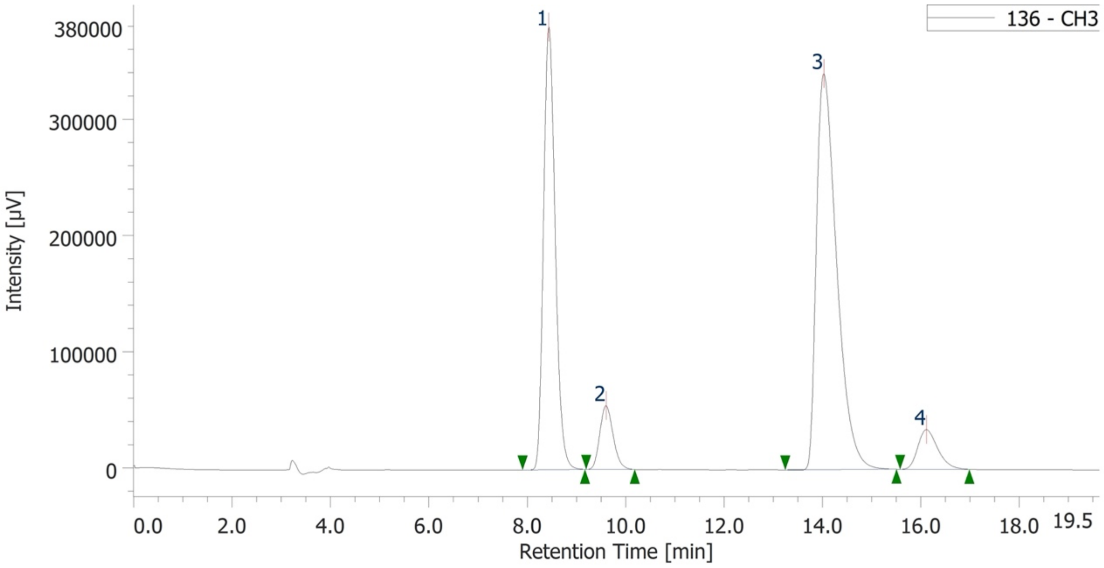

#	Peak Name	CH	tR [min]	Area [μV·sec]	Height [μV]	Area%	Height%
1	Unknown	3	8.43	6273137	380449	34.837	46.985
2	Unknown	3	9.60	1034832	54810	5.747	6.769
3	Unknown	3	14.03	9742849	340388	54.106	42.038
4	Unknown	3	16.11	956166	34073	5.310	4.208

17b catalyzed by **9b** (Scheme 4)

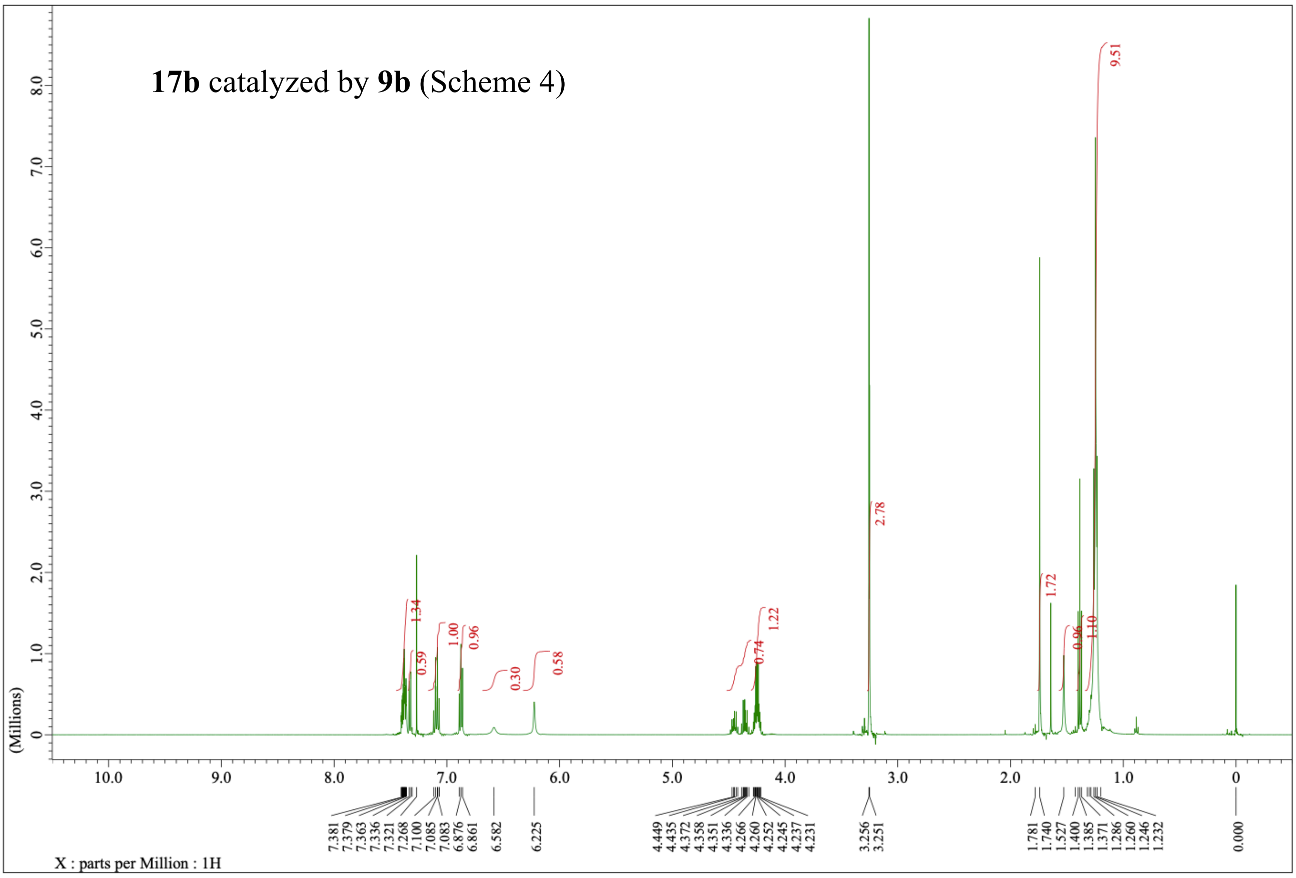

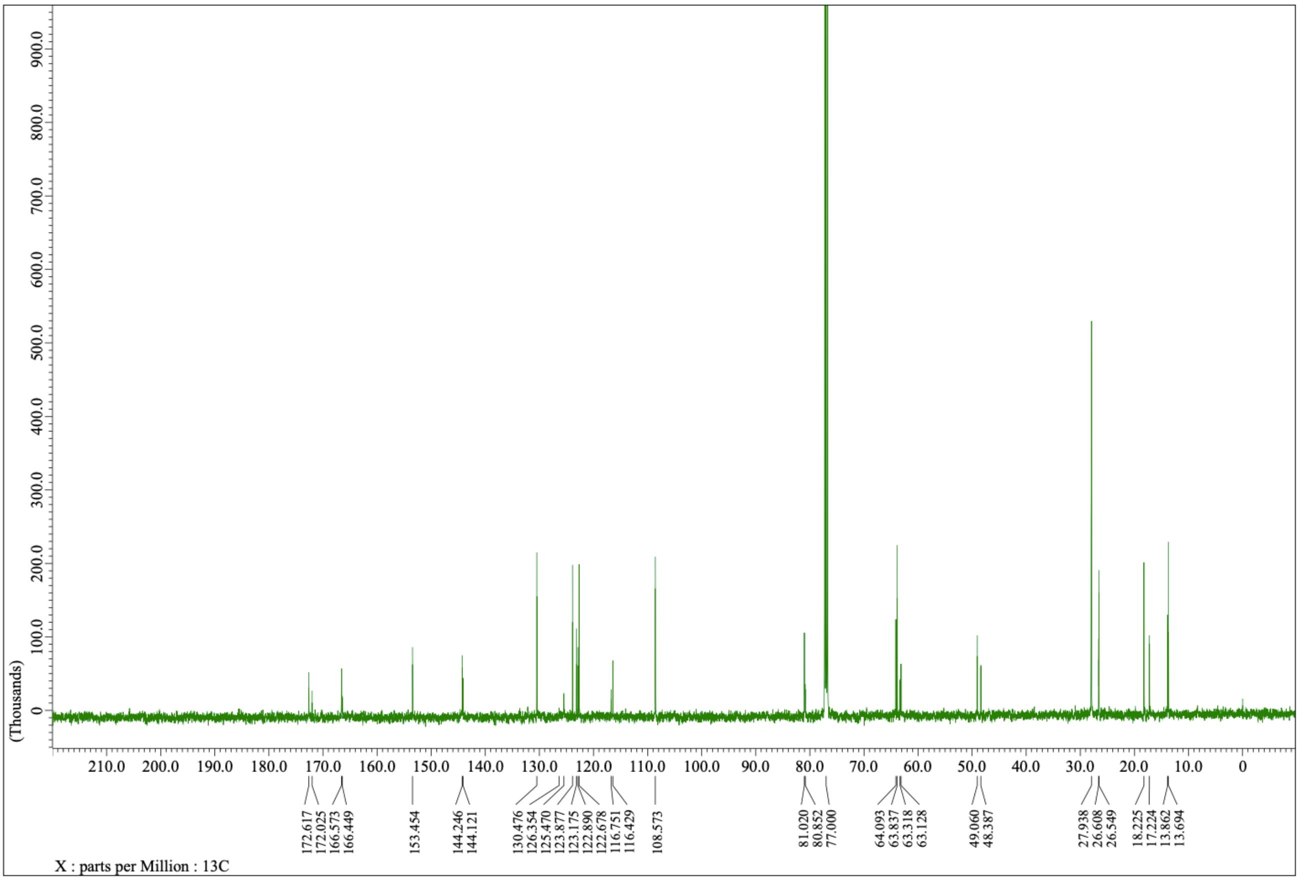

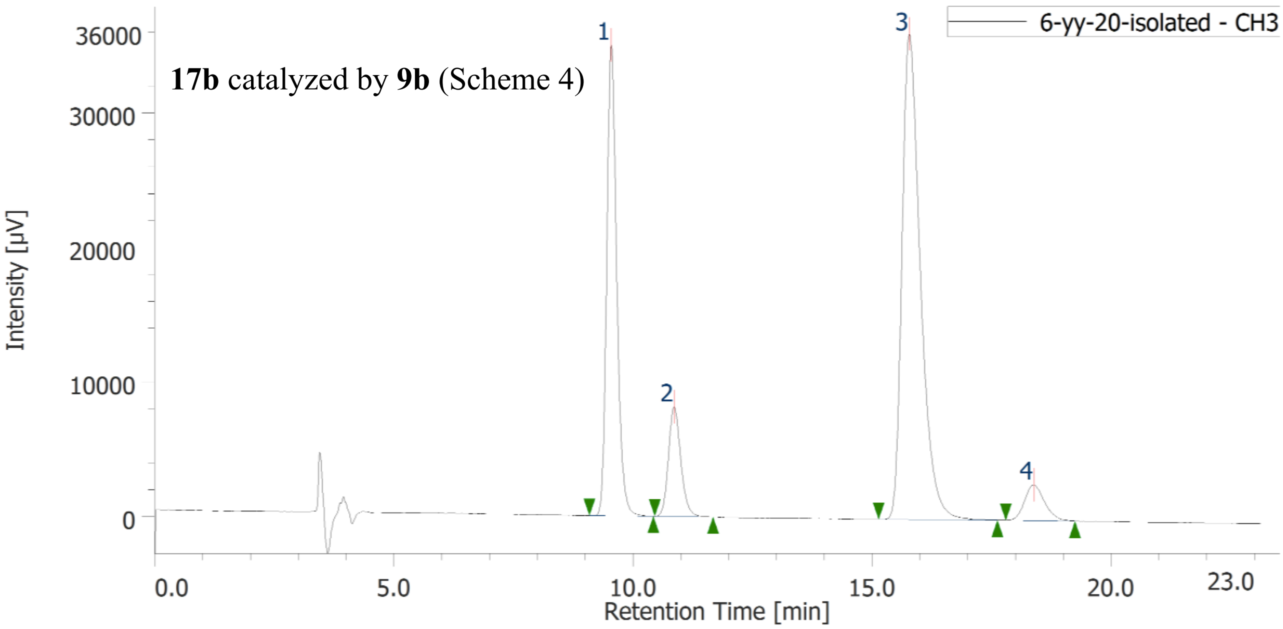

#	Peak Name	CH	tR [min]	Area [μV·sec]	Hight [μV]	Area%	Hight%
1	Unknown	3	9.542	506583	34966	29.92	42.7
2	Unknown	3	10.858	146582	8149	8.658	9.96
3	Unknown	3	15.775	963142	36056	56.89	44.1
4	Unknown	3	18.375	76808	2658	4.536	3.25

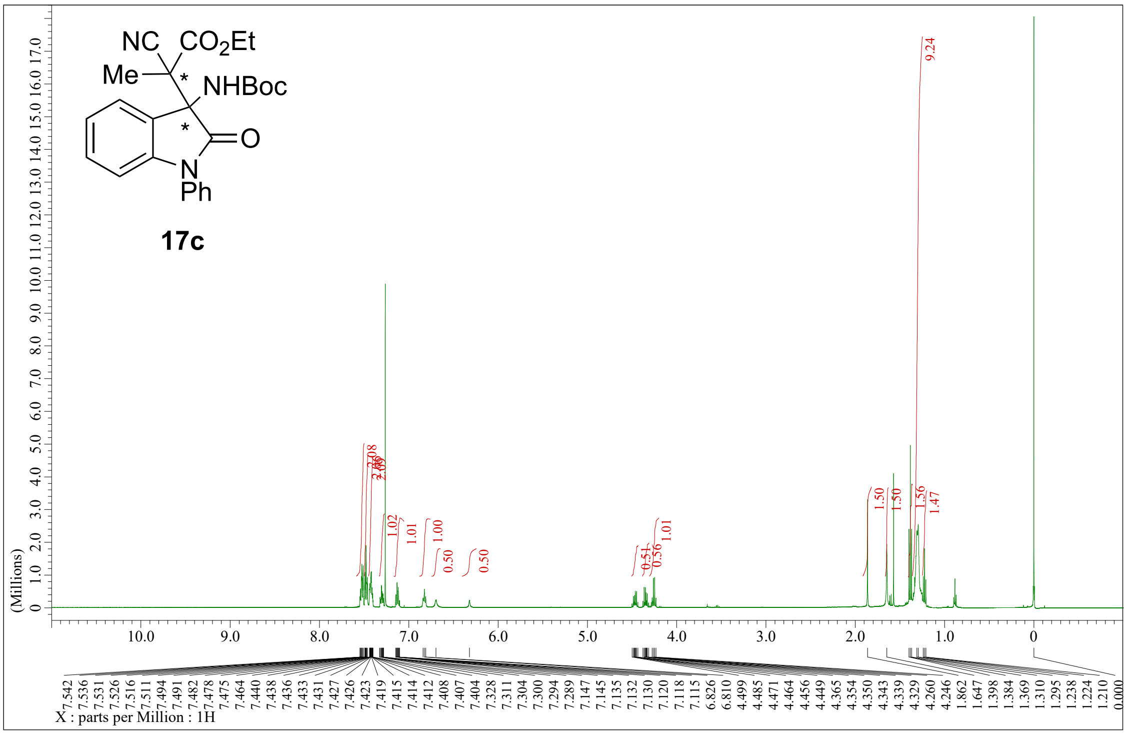
NC CO2Et
Me
NHBoc
O
N
Ph
17c
(Millions)
X : parts per Million : 1H
7.542 7.536 7.531 7.526 7.516 7.511 7.494 7.491 7.482 7.478 7.475 7.464 7.440 7.438 7.436 7.433 7.431 7.427 7.426 7.423 7.419 7.415 7.414 7.412 7.408 7.407 7.404 7.328 7.311 7.304 7.300 7.294 7.289 7.147 7.145 7.135 7.132 7.130 7.120 7.118 7.115 6.826 6.810 4.499 4.485 4.471 4.464 4.456 4.449 4.365 4.354 4.350 4.343 4.339 4.329 4.260 4.246 1.862 1.647 1.398 1.384 1.369 1.310 1.295 1.238 1.224 1.210 0.000

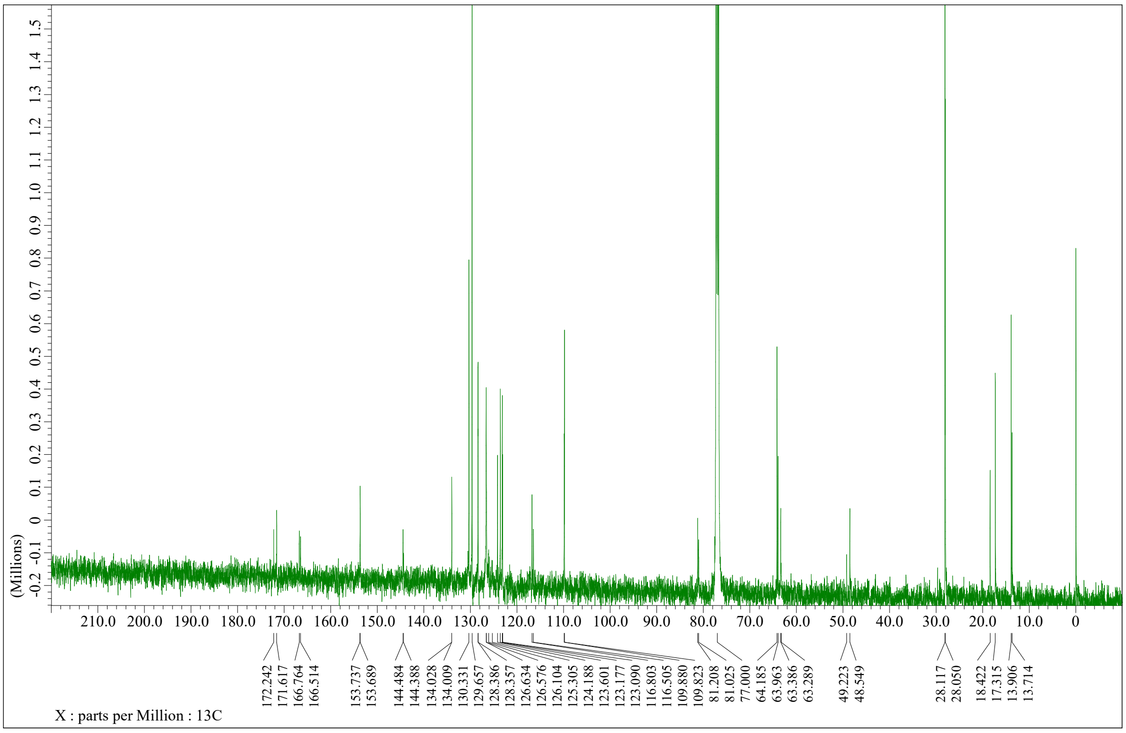
(Millions)
X : parts per Million : 13C
172.242 171.617 166.764 166.514 153.737 153.689 144.484 144.388 134.028 134.009 130.331 129.657 128.386 128.357 126.634 126.576 126.104 125.305 124.188 123.601 123.177 123.090 116.803 116.505 109.880 109.823 81.208 81.025 77.000 64.185 63.963 63.386 63.289 49.223 48.549 28.117 28.050 18.422 17.315 13.906 13.714

Racemic sample of **17c**

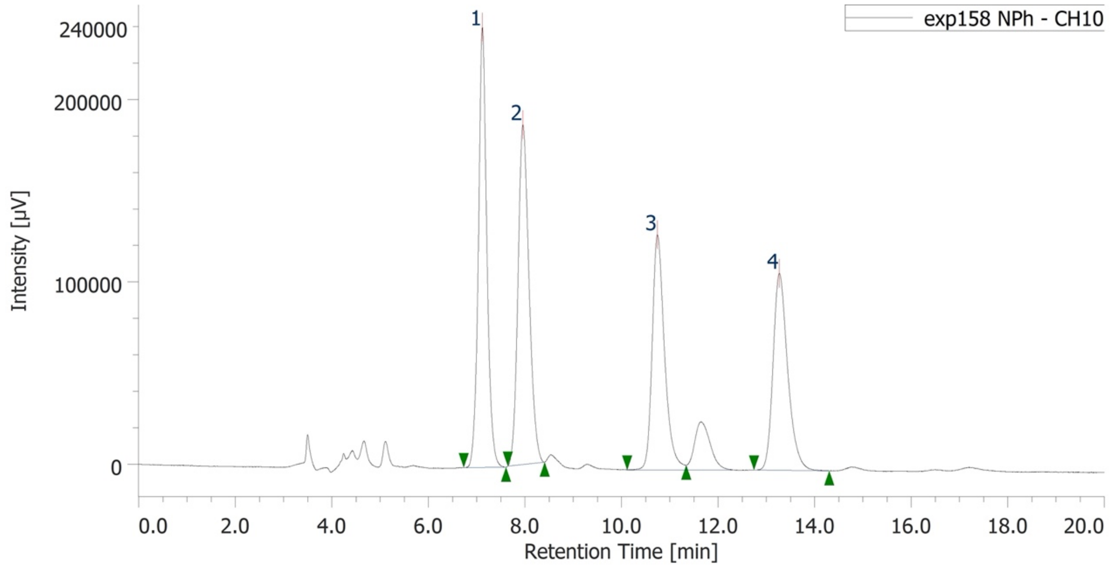

#	Peak Name	CH	tR [min]	Area [μV·sec]	Height [μV]	Area%	Height%
1	Unknown	10	7.12	2924061	241226	28.240	36.403
2	Unknown	10	7.96	2948906	186083	28.480	28.081
3	Unknown	10	10.74	2199689	127447	21.244	19.233
4	Unknown	10	13.27	2281790	107898	22.037	16.283

17c catalyzed by **9a**

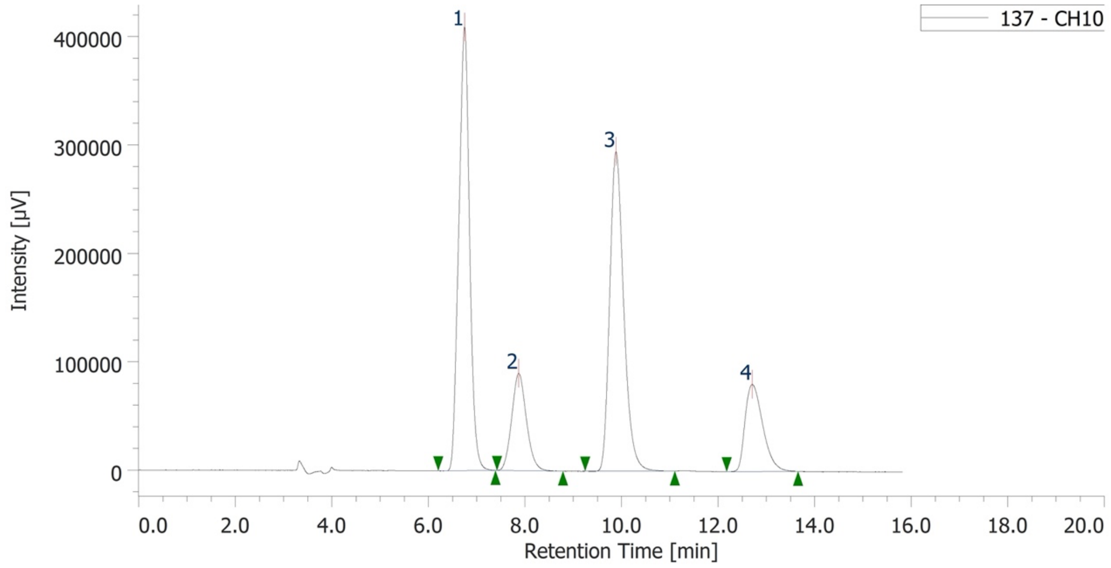

#	Peak Name	CH	tR [min]	Area [μV·sec]	Height [μV]	Area%	Height%
1	Unknown	10	6.75	6347149	409009	39.128	46.815
2	Unknown	10	7.87	1890829	89680	11.656	10.265
3	Unknown	10	9.88	5931257	294672	36.564	33.728
4	Unknown	10	12.71	2052216	80309	12.651	9.192

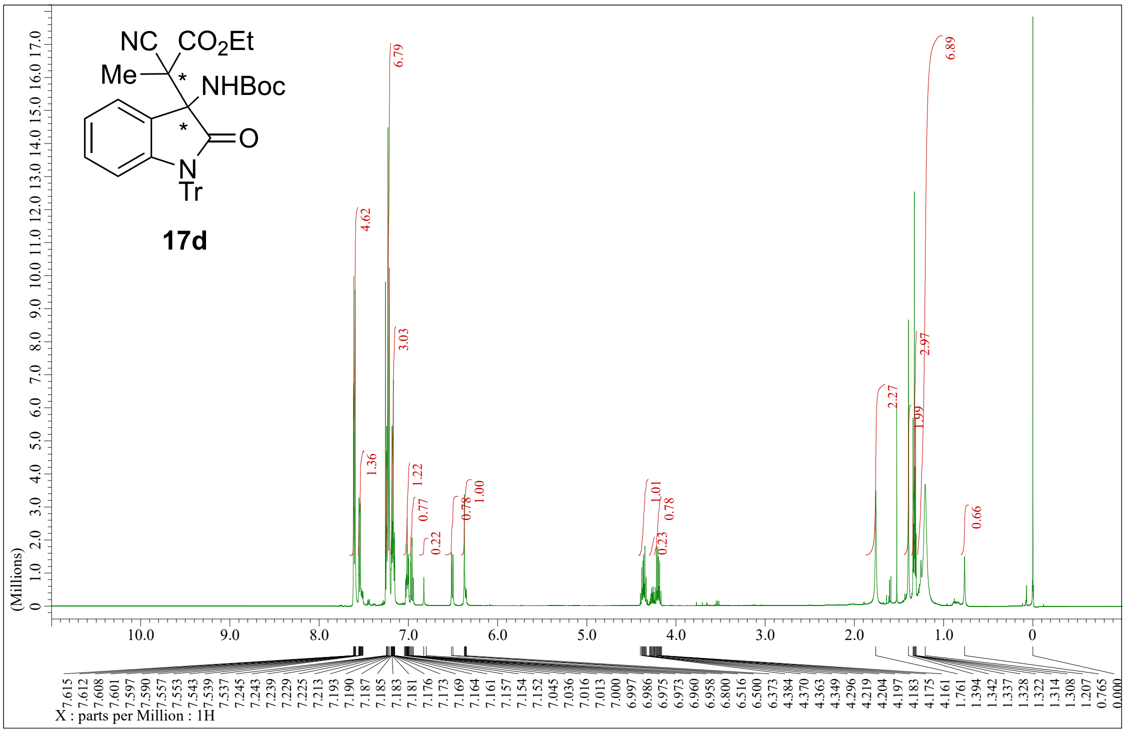
NC CO2Et
Me
NHBoc
O
N
Tr
17d
(Millions)
4.62 1.36 6.79 3.03 1.22 0.77 0.22 0.78 1.00 1.01 0.23 0.78 2.27 1.99 2.97 6.89 0.66
7.615 7.612 7.608 7.601 7.597 7.590 7.557 7.553 7.543 7.539 7.537 7.245 7.243 7.239 7.229 7.225 7.213 7.193 7.190 7.187 7.185 7.183 7.181 7.176 7.173 7.169 7.164 7.161 7.157 7.154 7.152 7.045 7.036 7.016 7.013 7.000 6.997 6.986 6.975 6.973 6.960 6.958 6.800 6.516 6.500 6.373 4.384 4.370 4.363 4.349 4.296 4.219 4.204 4.197 4.183 4.175 4.161 1.761 1.394 1.342 1.337 1.328 1.322 1.314 1.308 1.207 0.765 0.000
X : parts per Million : 1H

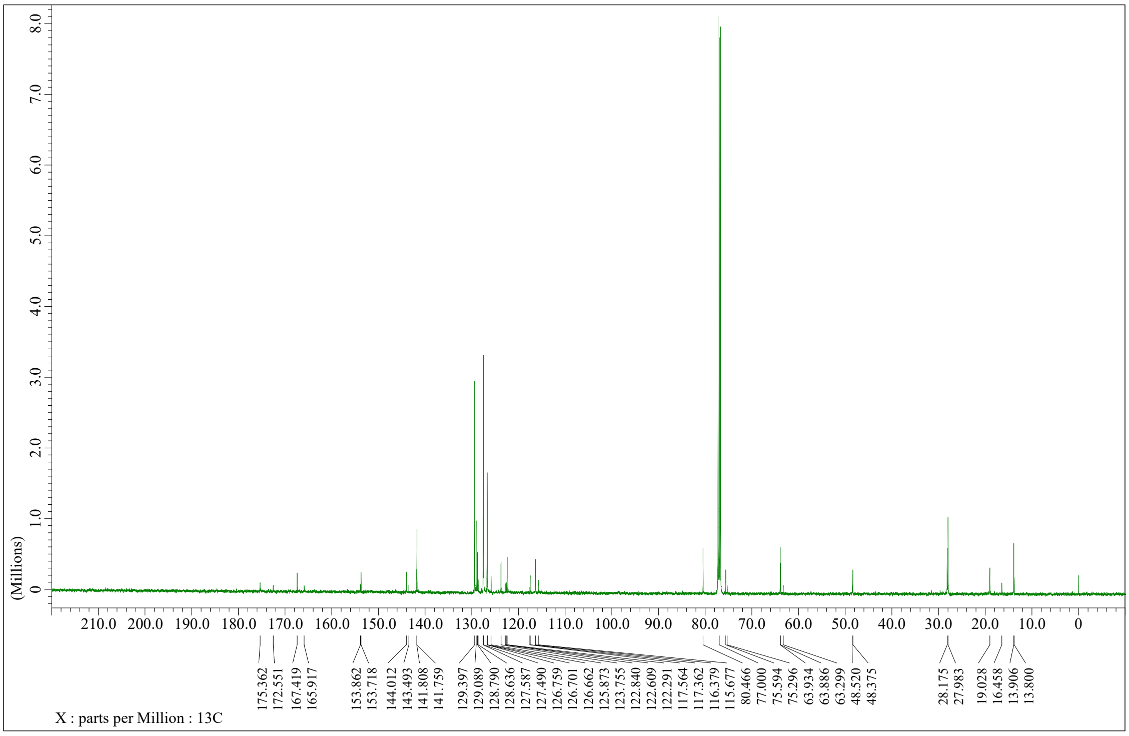
(Millions)
175.362 172.551 167.419 165.917 153.862 153.718 144.012 143.493 141.808 141.759 129.397 129.089 128.790 128.636 127.587 127.490 126.759 126.701 126.662 125.873 123.755 122.840 122.609 122.291 117.564 117.362 116.379 115.677 80.466 77.000 75.594 75.296 63.934 63.886 63.299 48.520 48.375 28.175 27.983 19.028 16.458 13.906 13.800
X : parts per Million : 13C

Racemic sample of **17d**

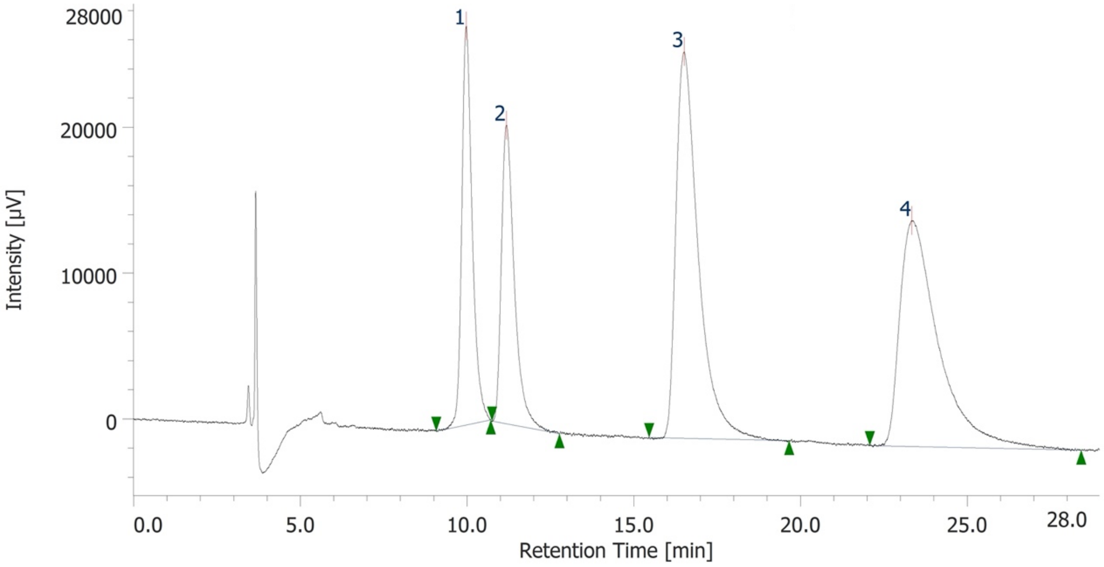

#	Peak Name	CH	tR [min]	Area [µV·sec]	Height [µV]	Area%	Height%
1	Unknown	10	9.97	593764	27336	16.379	30.448
2	Unknown	10	11.18	552546	20474	15.242	22.804
3	Unknown	10	16.51	1236065	26491	34.097	29.506
4	Unknown	10	23.34	1242736	15481	34.281	17.243

17d catalyzed by **9a**

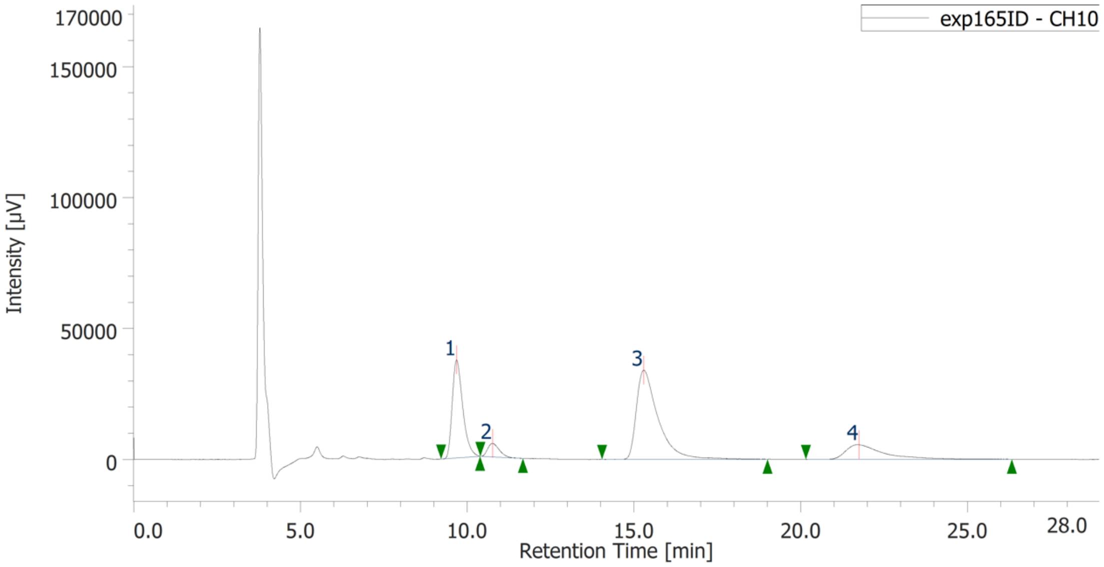

#	Peak Name	CH	tR [min]	Area [µV·sec]	Height [µV]	Area%	Height%
1	Unknown	10	9.677	820662	37432	28.035	45.5
2	Unknown	10	10.757	124860	5182	4.265	6.30
3	Unknown	10	15.287	1534800	34042	52.431	41.4
4	Unknown	10	21.740	446954	5574	15.269	6.78

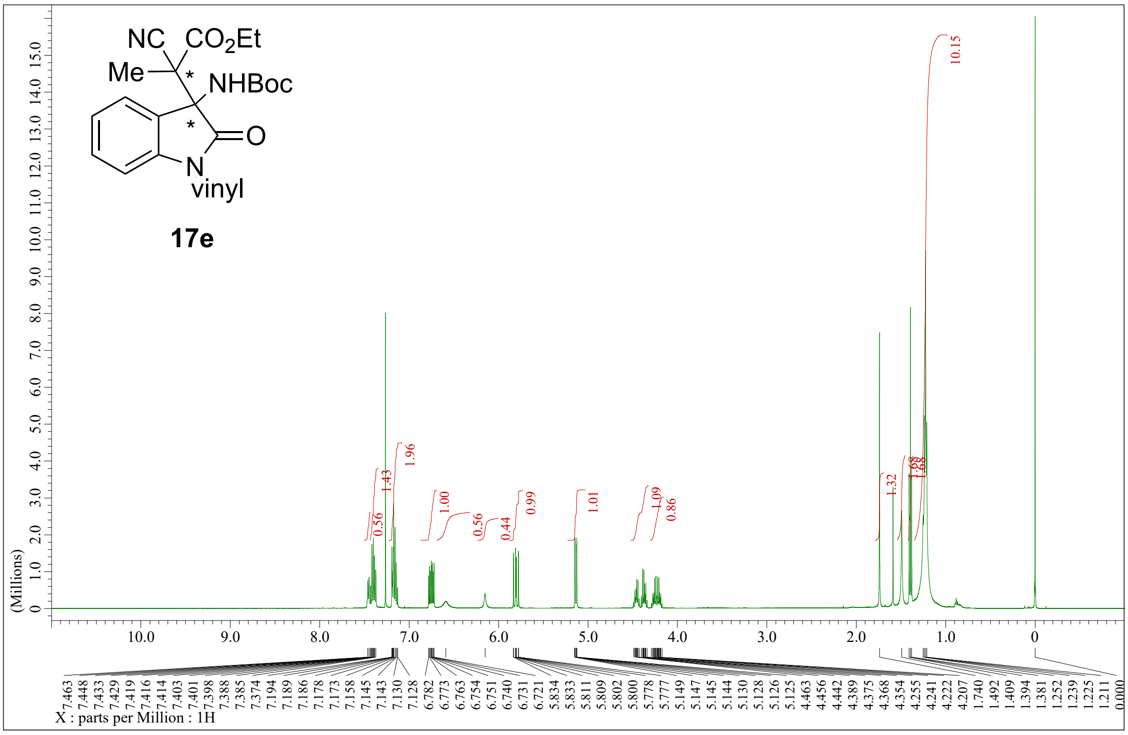
NC CO2Et
Me
NHBoc
N
vinyl
17e
X : parts per Million : 1H
(Millions)

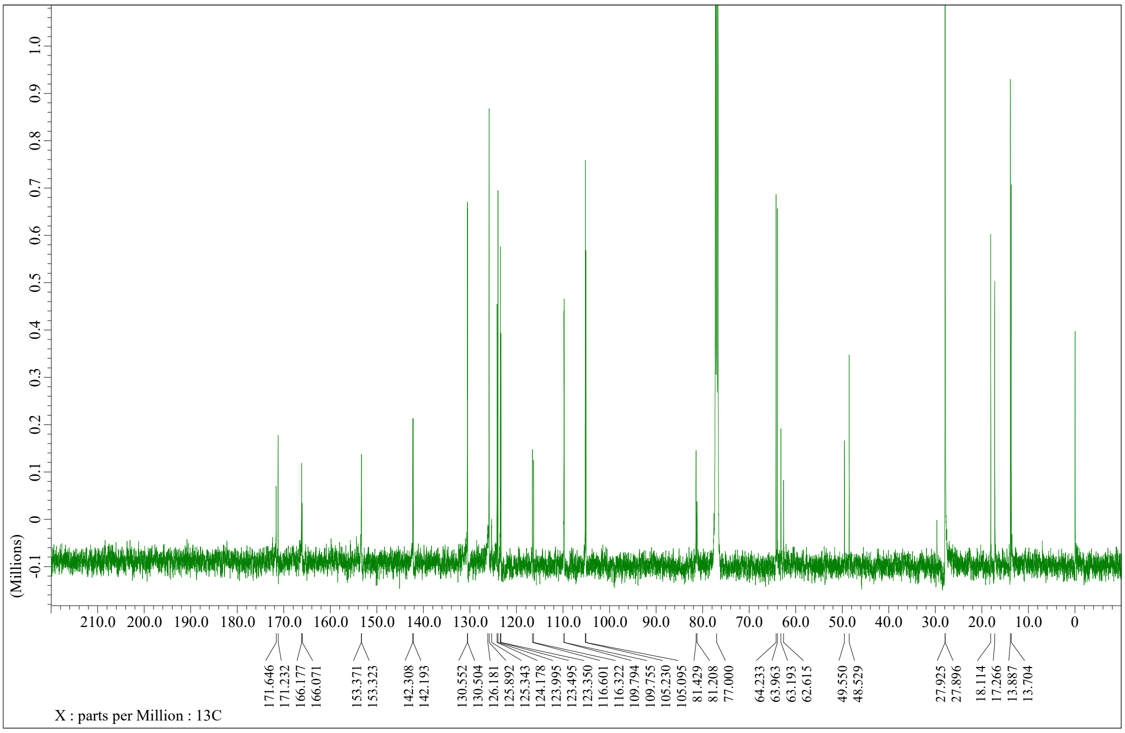
X : parts per Million : 13C
(Millions)

Racemic sample of **17e**

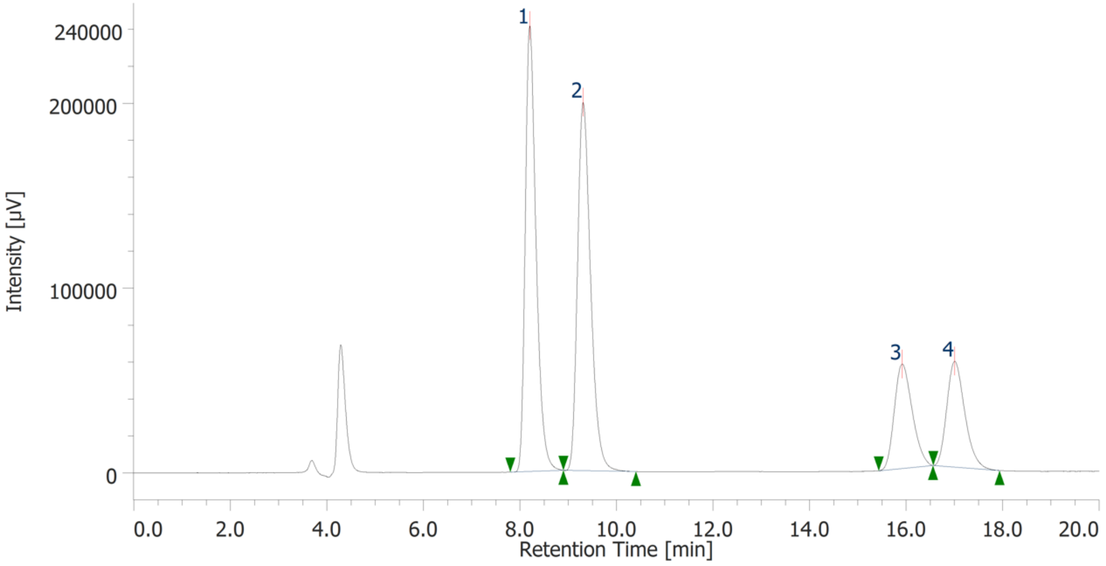

#	Peak Name	CH	tR [min]	Area [μV·sec]	Height [μV]	Area%	Height%
1	Unknown	9	8.200	3744670	241147	35.901	43.5
2	Unknown	9	9.307	3746009	199213	35.913	36.0
3	Unknown	9	15.917	1458588	56498	13.984	10.2
4	Unknown	9	17.003	1481391	57269	14.202	10.3

17e catalyzed by **9a**

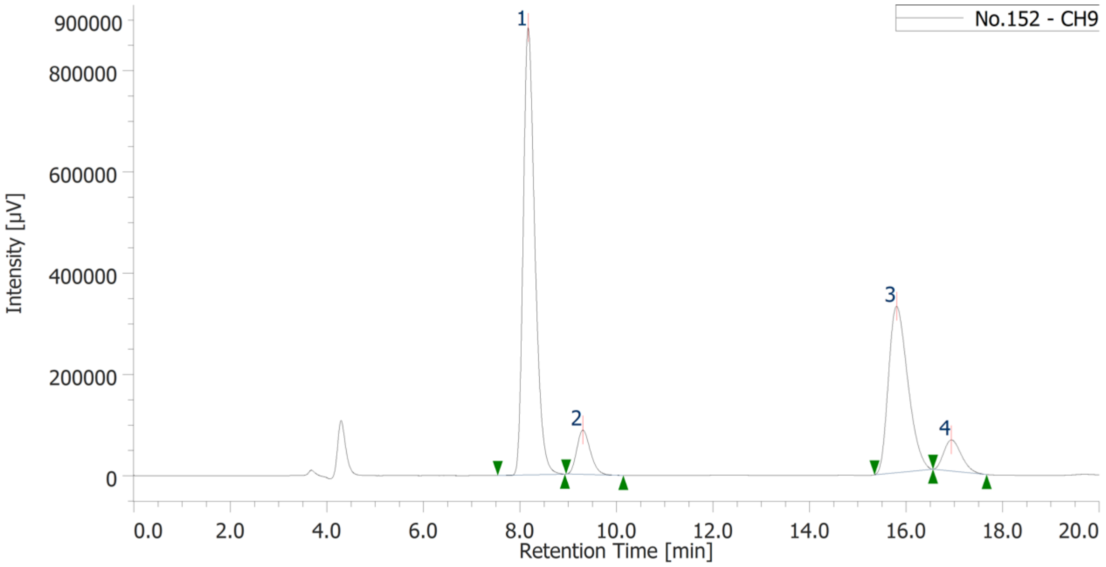

#	Peak Name	CH	tR [min]	Area [μV·sec]	Height [μV]	Area%	Height%
1	Unknown	9	8.170	14856358	883198	54.381	64.9
2	Unknown	9	9.300	1712639	87525	6.269	6.43
3	Unknown	9	15.800	9191246	328575	33.644	24.1
4	Unknown	9	16.933	1558747	61485	5.706	4.52

・^{1}H-NMR and HPLC charts of **17b** for **Scheme 3**.

Without catalyst (65% yield, dr 19 : 81)

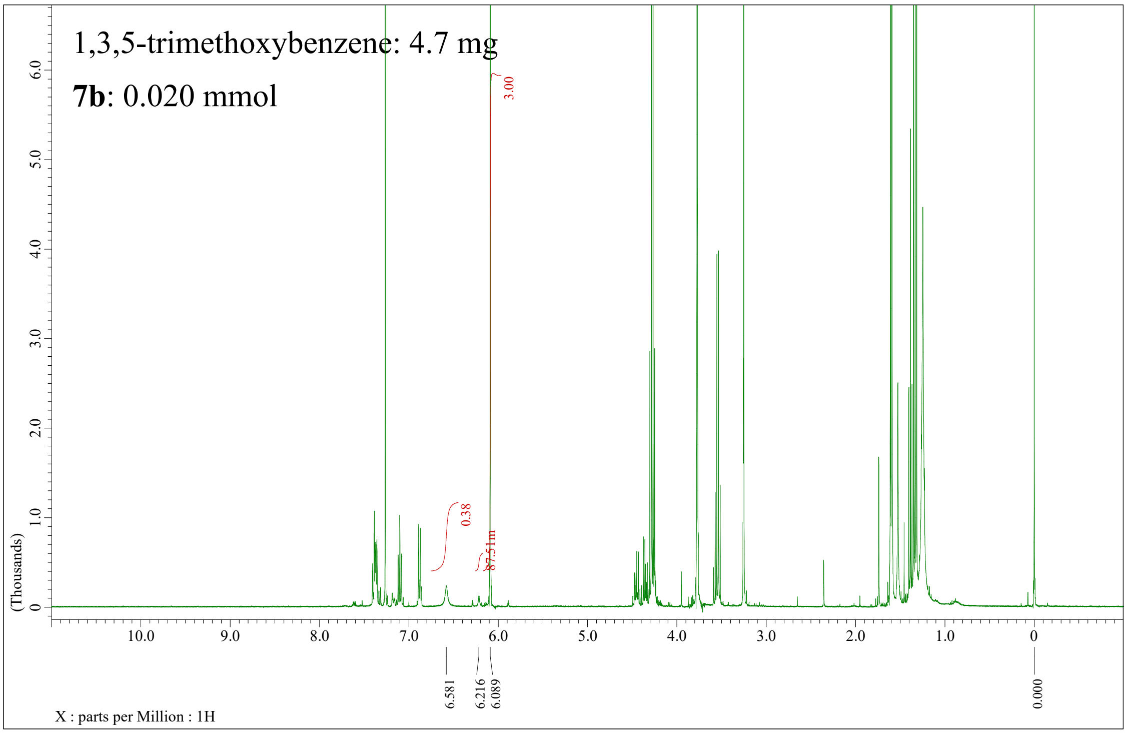

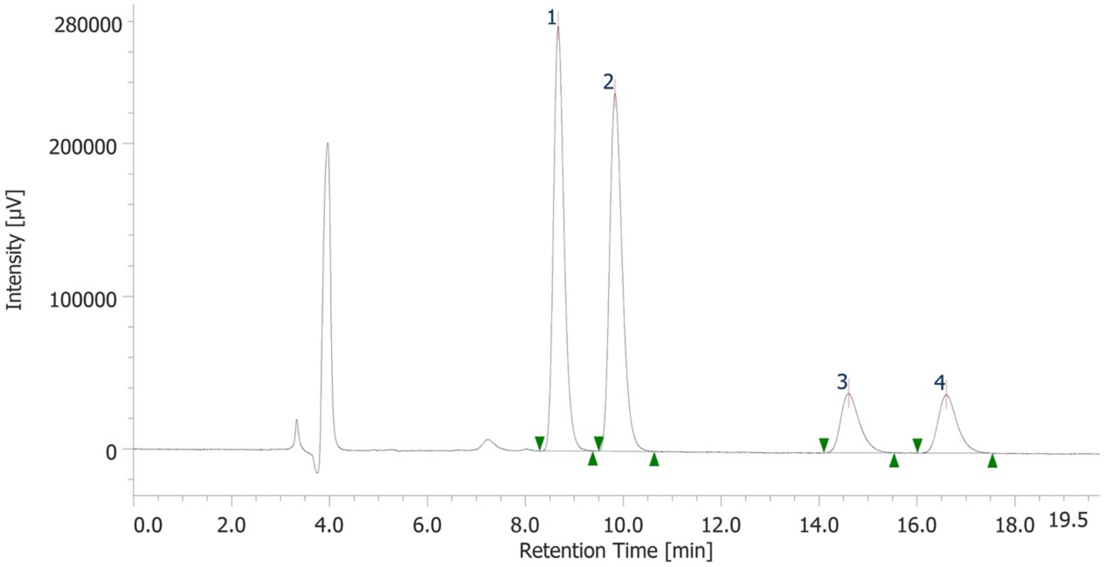

#	Peak Name	CH	tR [min]	Area [μV·sec]	Height [μV]	Area%	Height%
1	Unknown	9	8.67	4178901	278131	40.051	47.190
2	Unknown	9	9.83	4190131	234158	40.159	39.729
3	Unknown	9	14.60	1033791	38783	9.908	6.580
4	Unknown	9	16.59	1031144	38311	9.883	6.500

Catalyzed by **9a** (81% yield, dr 59 : 41)

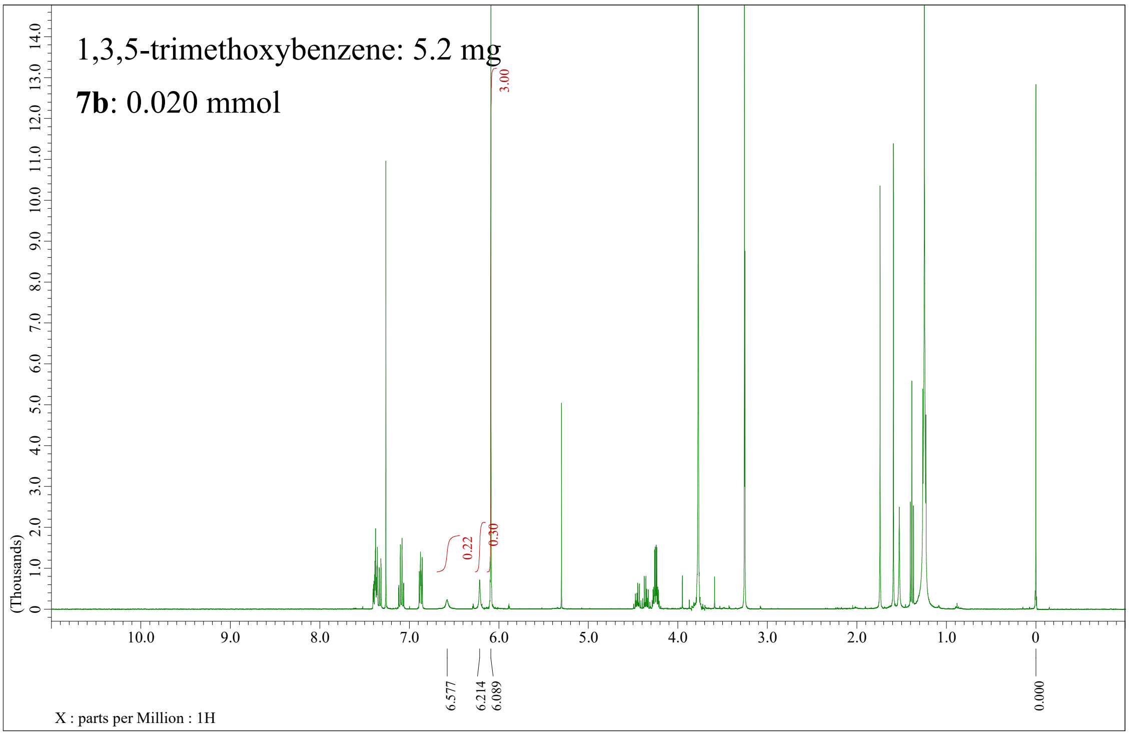

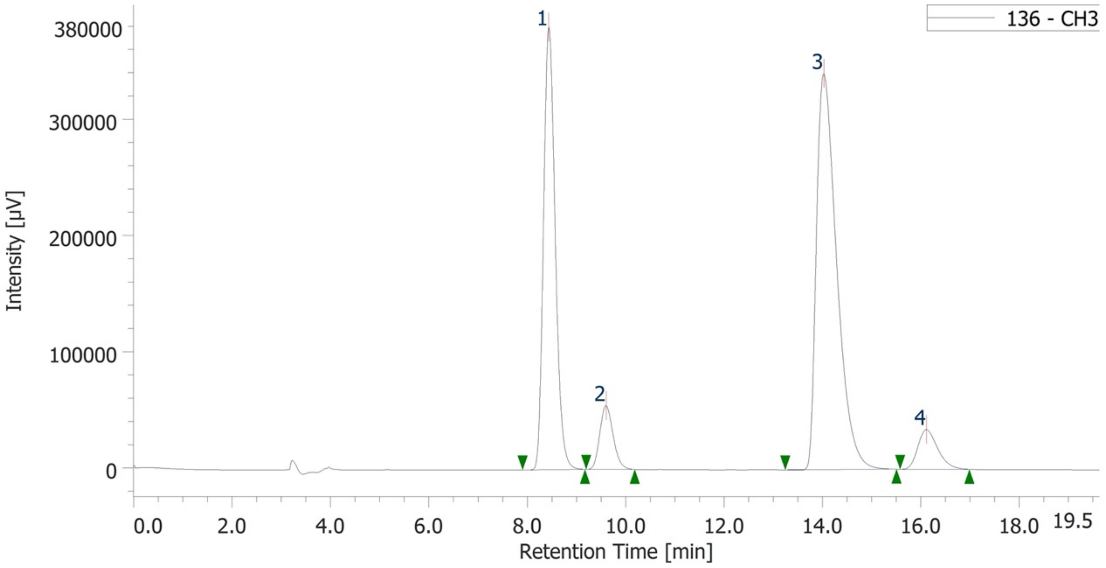

#	Peak Name	CH	tR [min]	Area [µV·sec]	Height [µV]	Area%	Height%
1	Unknown	3	8.43	6273137	380449	34.837	46.985
2	Unknown	3	9.60	1034832	54810	5.747	6.769
3	Unknown	3	14.03	9742849	340388	54.106	42.038
4	Unknown	3	16.11	956166	34073	5.310	4.208

Catalyzed by **9b** (98% yield, dr 67 : 33)

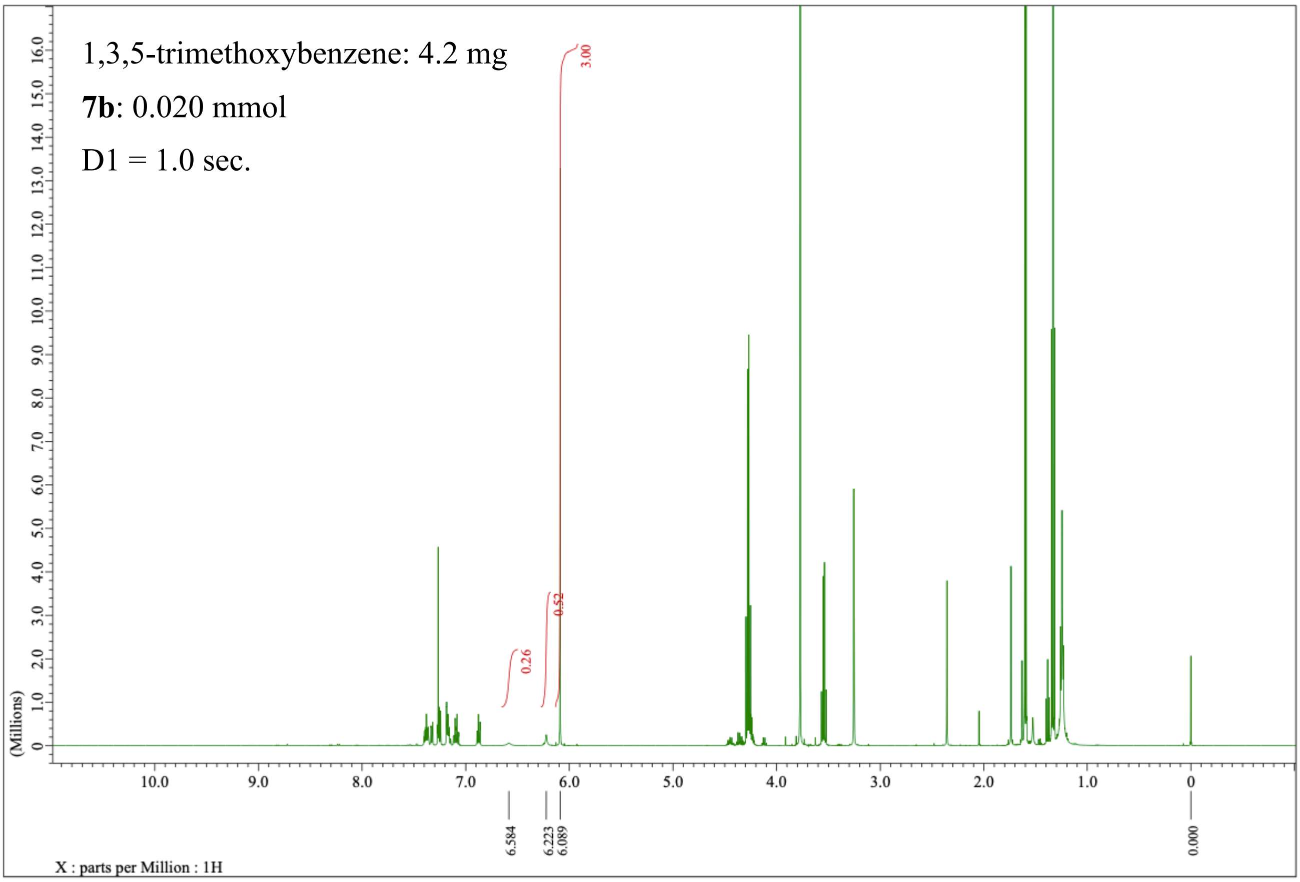

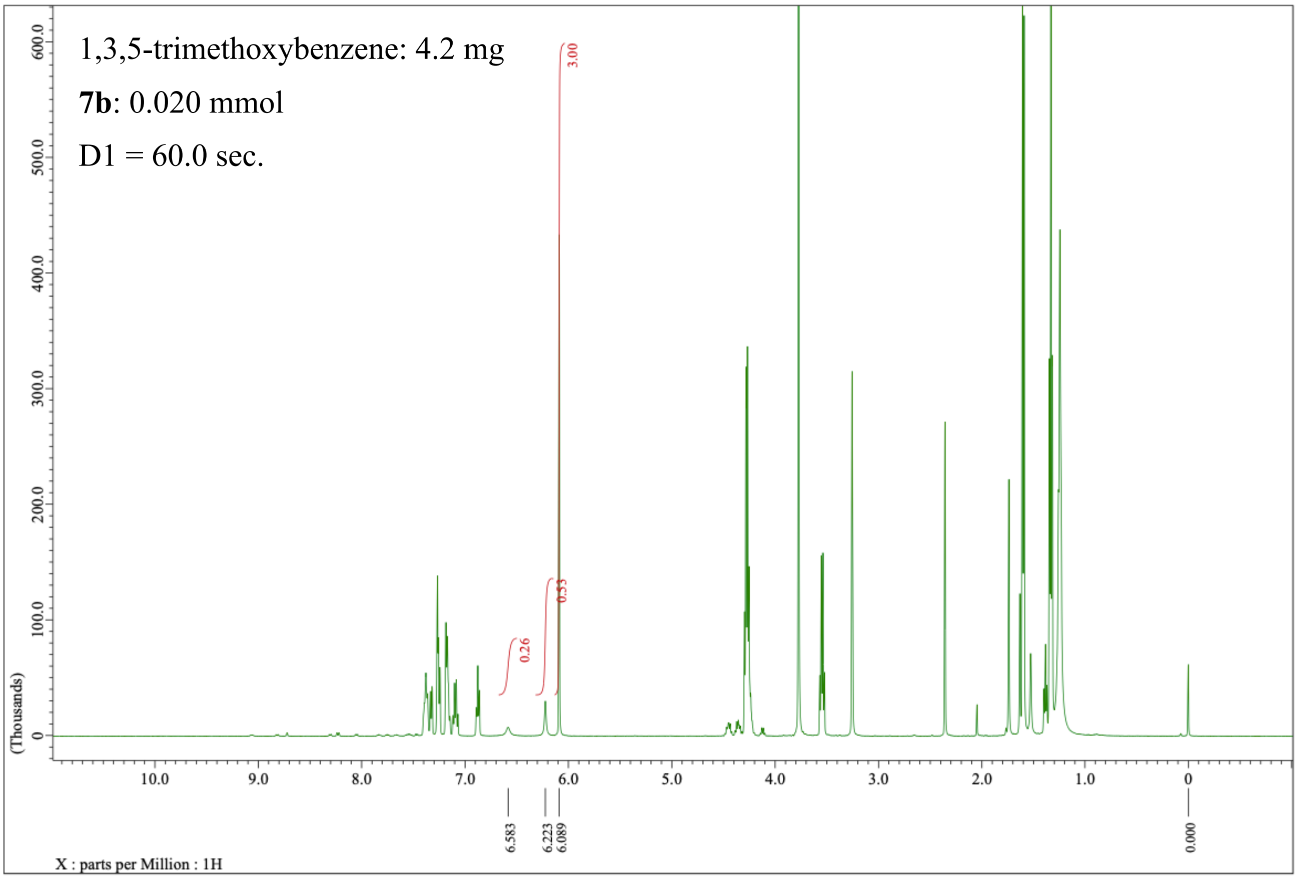

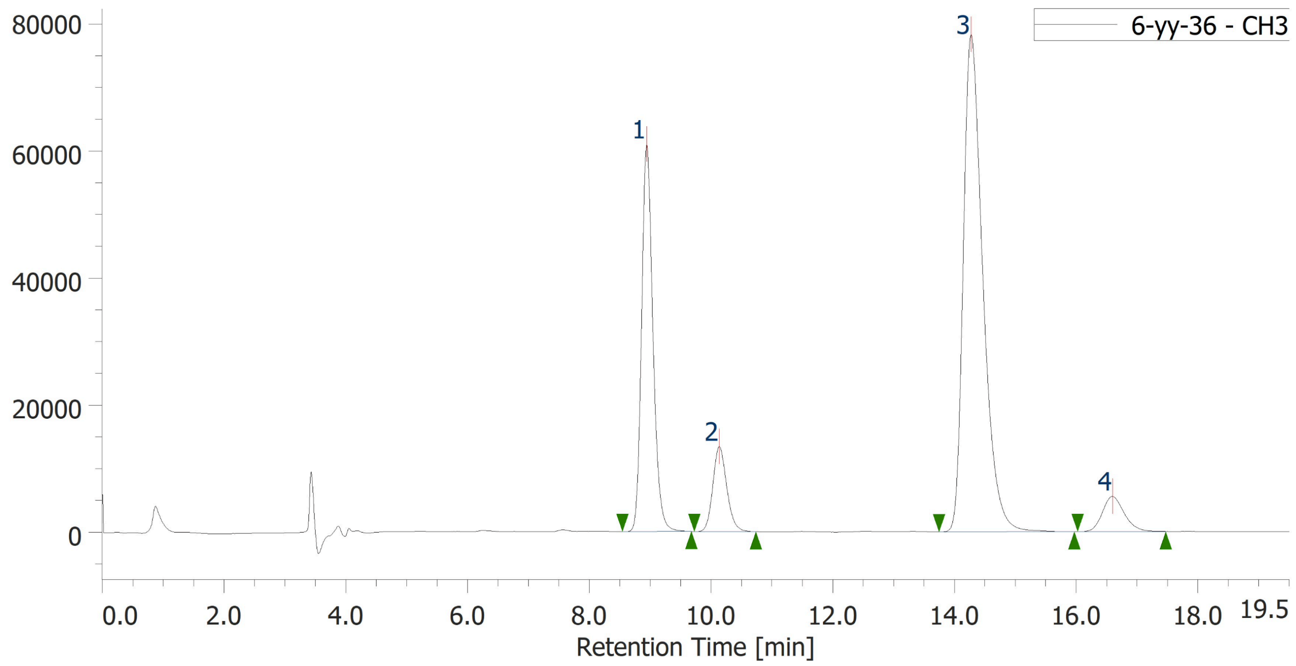

#	Peak Name	CH	tR [min]	Area [µV·sec]	Height [µV]	Area%	Height%
1	Unknown	3	8.94	788659	60939	27.32	38.6
2	Unknown	3	10.13	212754	13374	7.371	8.46
3	Unknown	3	14.27	1747957	78227	60.56	49.5
4	Unknown	3	16.59	136859	5526	4.742	3.50

Catalyzed by **9c** (72% yield, dr 42 : 58)

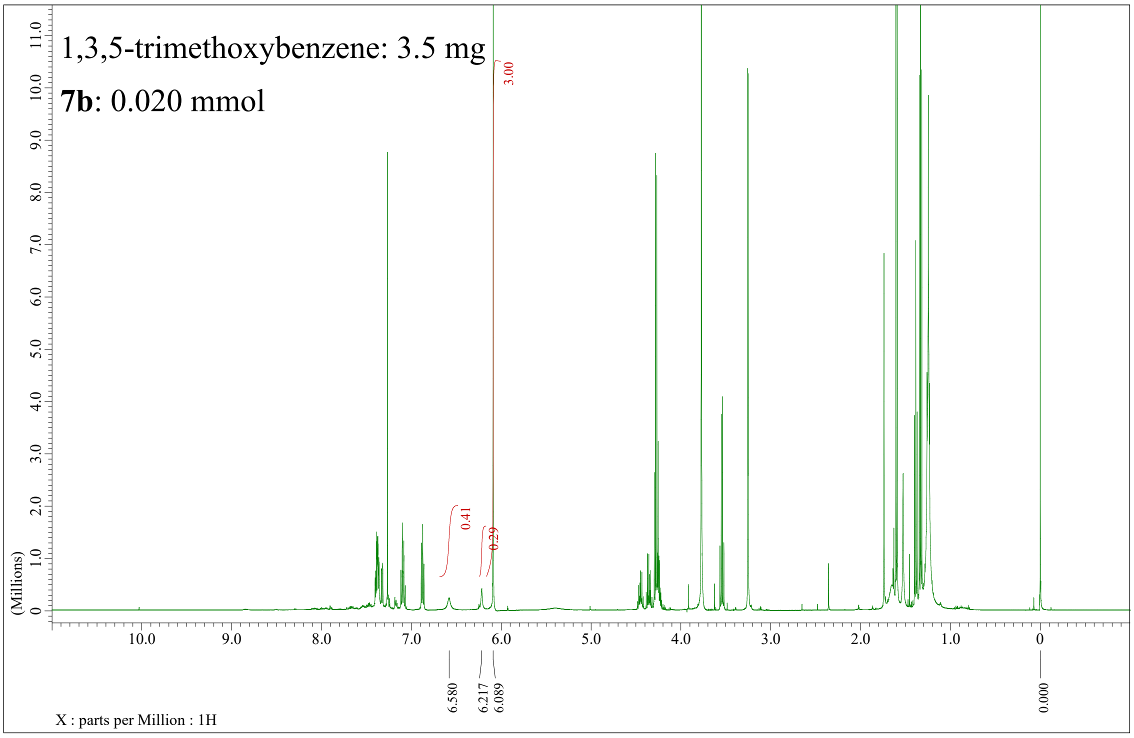

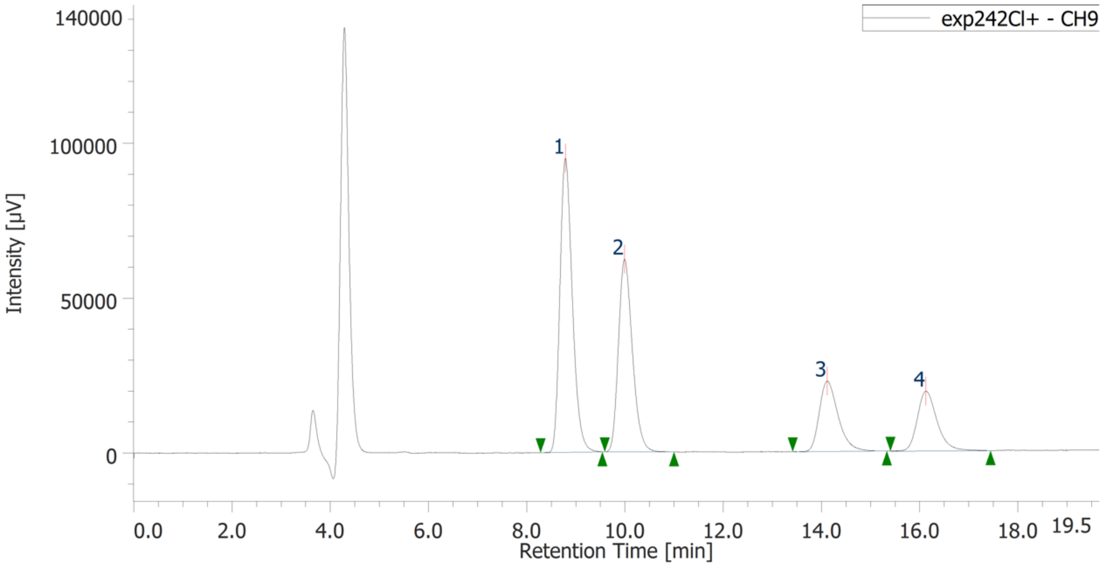

#	Peak Name	CH	tR [min]	Area [µV·sec]	Height [µV]	Area%	Height%
1	Unknown	9	8.783	1662920	94845	41.275	47.7
2	Unknown	9	9.990	1225330	62133	30.414	31.2
3	Unknown	9	14.113	603926	22690	14.990	11.4
4	Unknown	9	16.123	536678	19311	13.321	9.70

Catalyzed by **9d** (39% yield, dr 19 : 81)

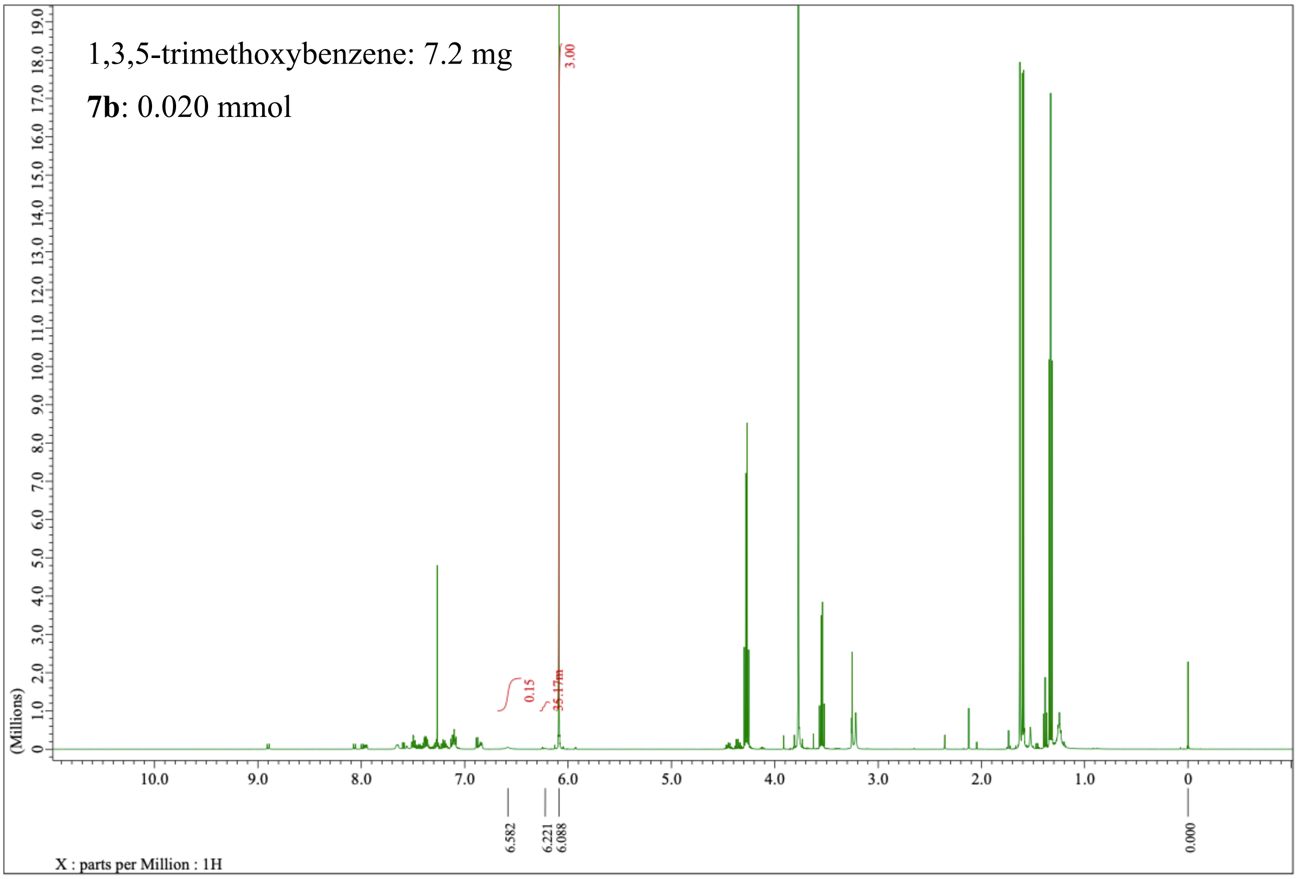

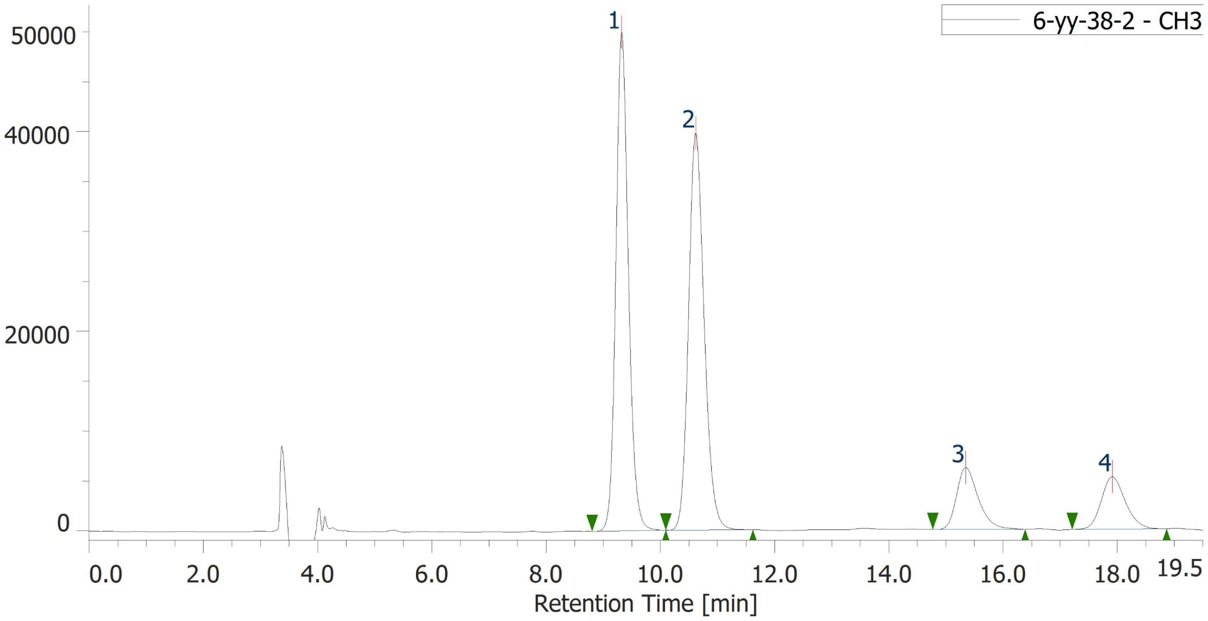

#	Peak Name	CH	tR [min]	Area [μV·sec]	Height [μV]	Area%	Height%
1	Unknown	3	9.32	759995	49939	41.51	49.4
2	Unknown	3	10.62	754664	39740	41.22	39.3
3	Unknown	3	15.34	163417	6192	8.926	6.12
4	Unknown	3	17.91	152671	5247	8.339	5.19

Catalyzed by **9e** (96% yield, dr 29 : 71)

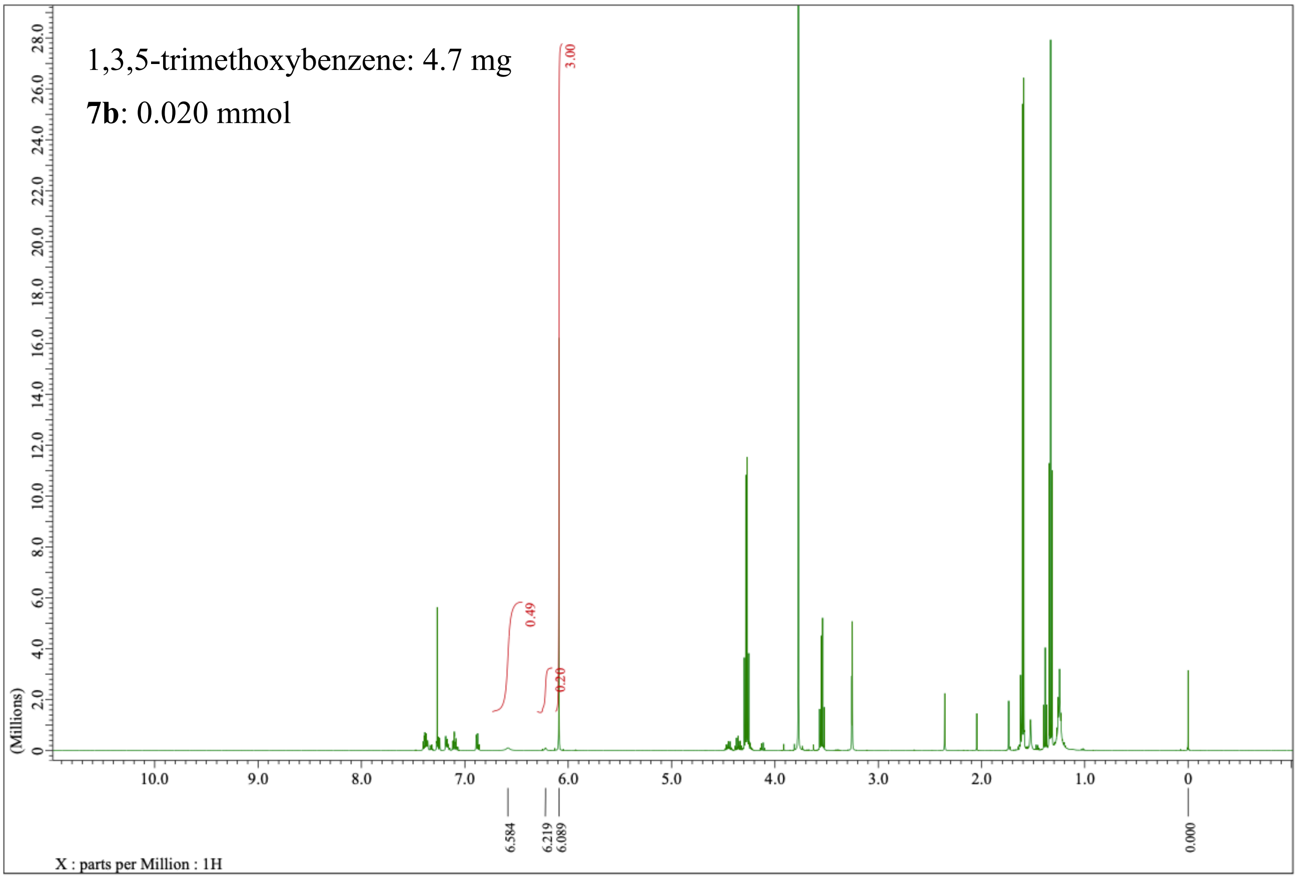

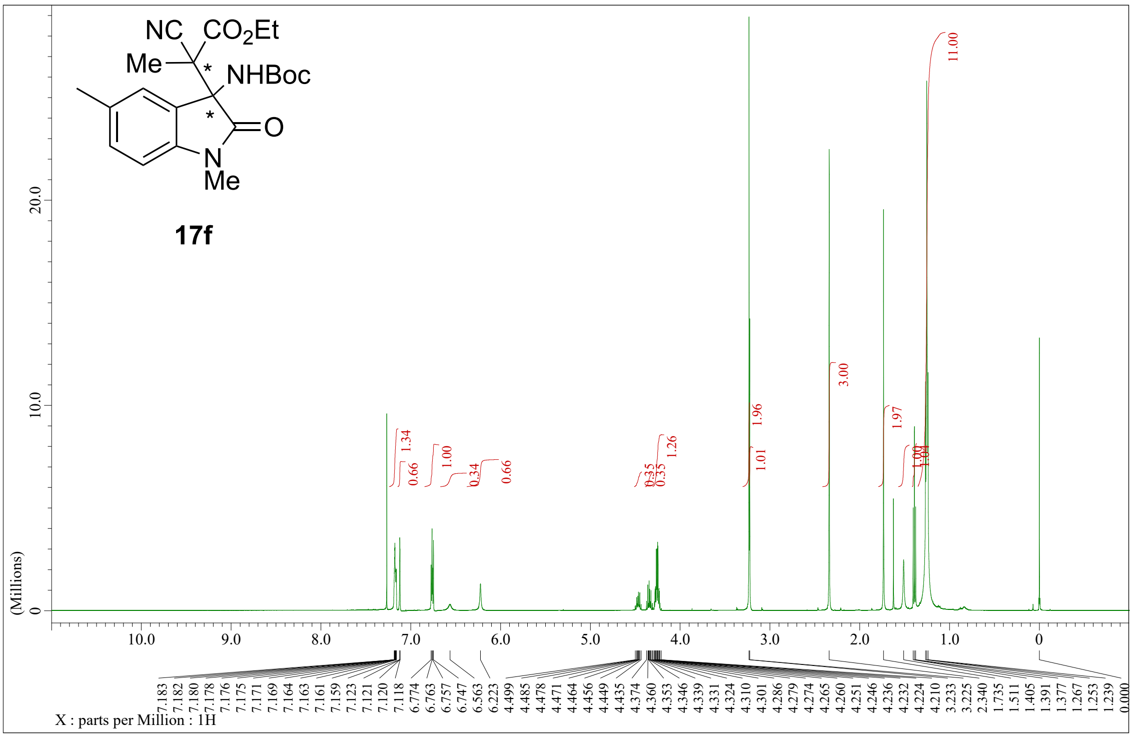

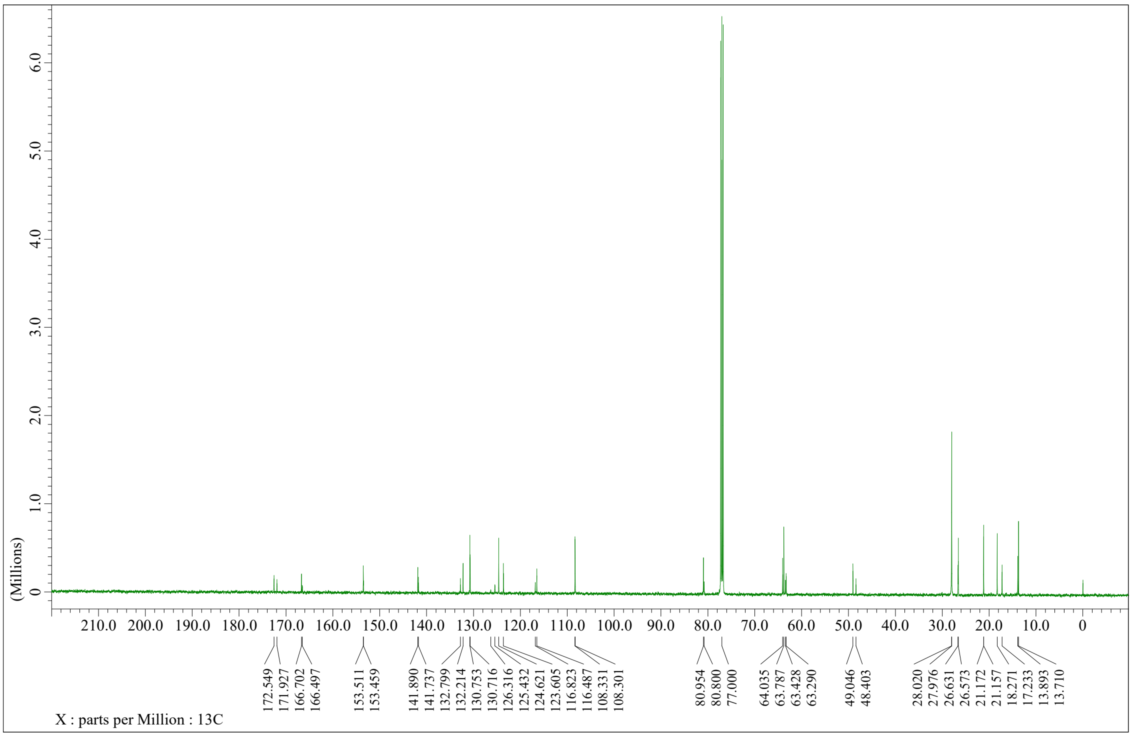

Racemic sample of **17f**

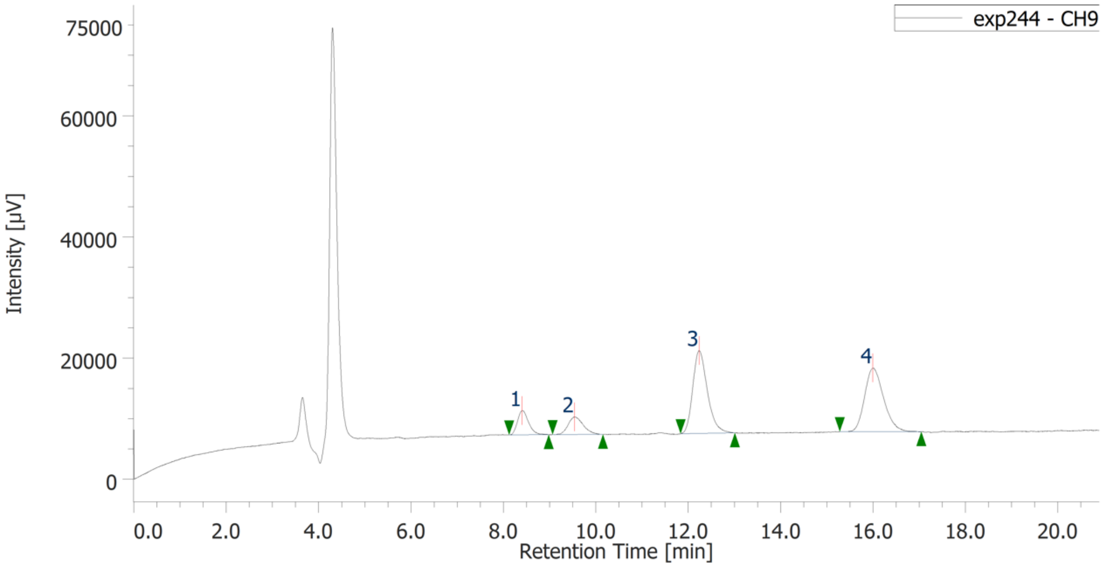

#	Peak Name	CH	tR [min]	Area [µV·sec]	Height [µV]	Area%	Height%
1	Unknown	9	8.403	65931	4000	9.010	12.9
2	Unknown	9	9.533	66312	2915	9.063	9.38
3	Unknown	9	12.233	300449	13657	41.061	43.9
4	Unknown	9	15.990	299018	10504	40.866	33.8

17f catalyzed by **9b**

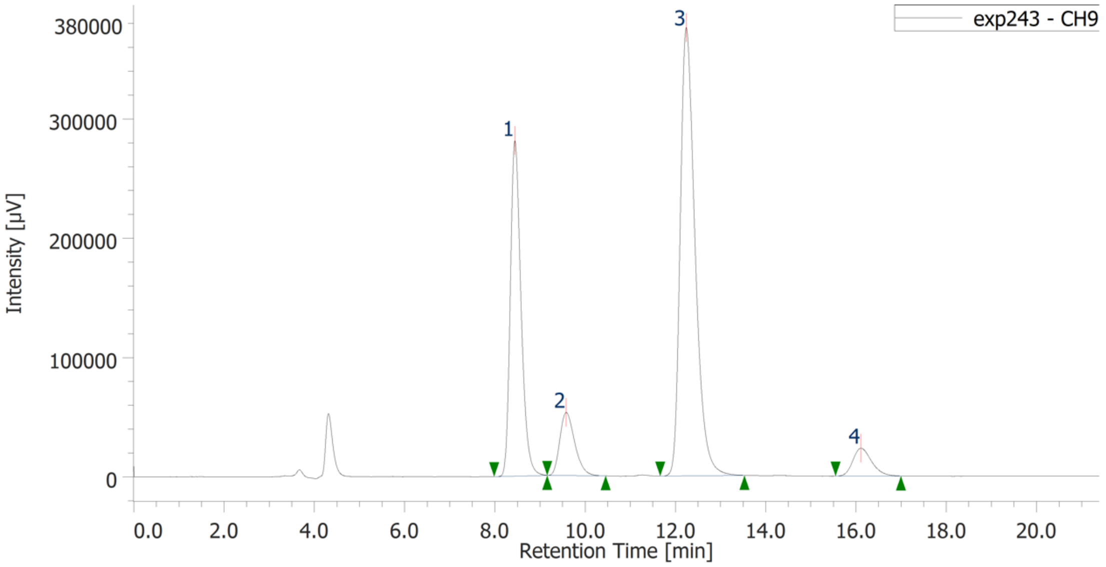

#	Peak Name	CH	tR [min]	Area [µV·sec]	Height [µV]	Area%	Height%
1	Unknown	9	8.440	4636523	281016	30.738	38.4
2	Unknown	9	9.577	1213980	52847	8.048	7.21
3	Unknown	9	12.240	8560817	375610	56.754	51.3
4	Unknown	9	16.103	672752	23291	4.460	3.18

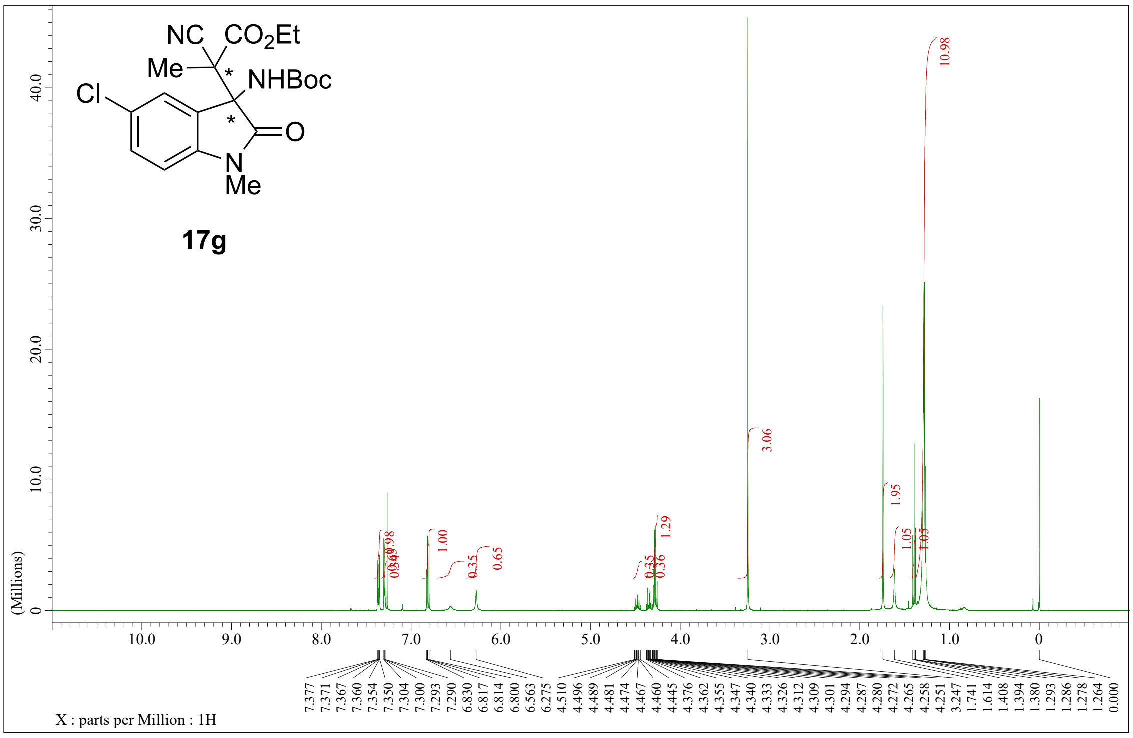

NC CO2Et
Me
NHBoc
Cl
O
N
Me
17g
(Millions)
X : parts per Million : 1H
10.98
3.06
1.95
1.05
1.03
1.29
0.35
0.36
1.00
0.35
0.65
7.377 7.371 7.367 7.360 7.354 7.350 7.304 7.300 7.293 7.290 6.830 6.817 6.814 6.800 6.563 6.275 4.510 4.496 4.489 4.481 4.474 4.467 4.460 4.445 4.376 4.362 4.355 4.347 4.340 4.333 4.326 4.312 4.309 4.301 4.294 4.287 4.280 4.272 4.265 4.258 4.251 3.247 1.741 1.614 1.408 1.394 1.380 1.293 1.286 1.278 1.264 0.000

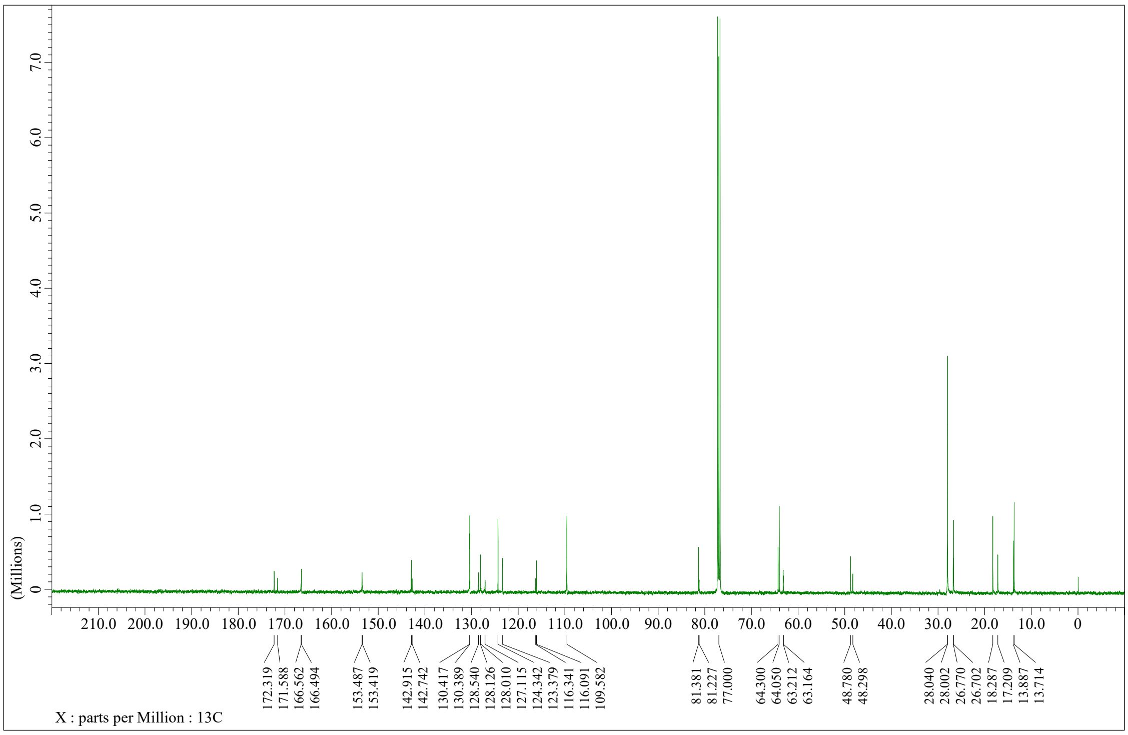

(Millions)
X : parts per Million : 13C
172.319 171.588 166.562 166.494 153.487 153.419 142.915 142.742 130.417 130.389 128.540 128.126 128.010 127.115 124.342 123.379 116.341 116.091 109.582 81.381 81.227 77.000 64.300 64.050 63.212 63.164 48.780 48.298 28.040 28.002 26.770 26.702 18.287 17.209 13.887 13.714

Racemic sample of **17g**

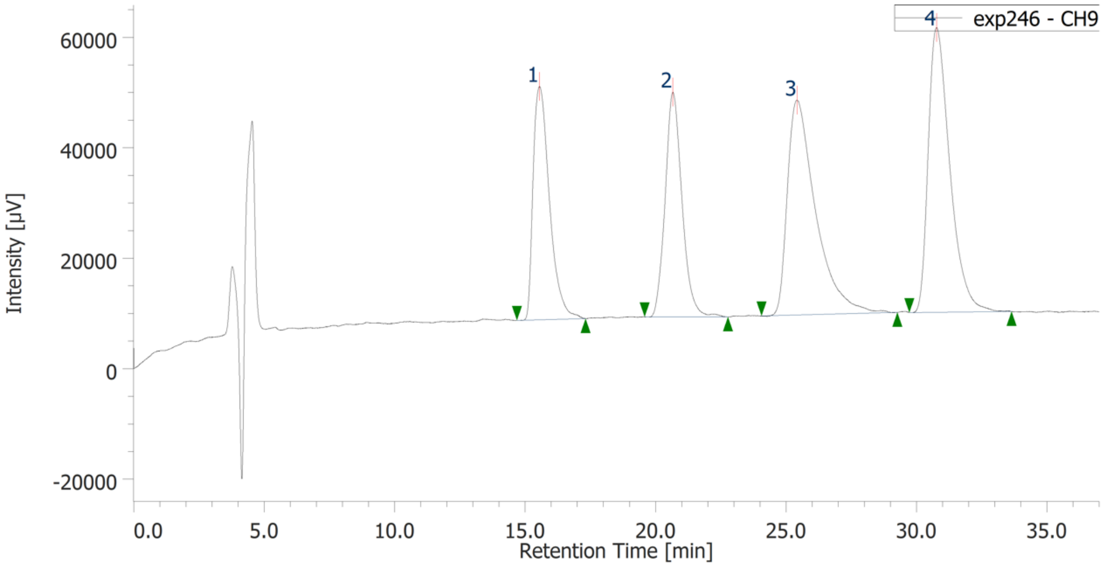

#	Peak Name	CH	tR [min]	Area [µV·sec]	Height [µV]	Area%	Height%
1	Unknown	9	15.543	1844183	42195	19.260	24.3
2	Unknown	9	20.653	1828552	40663	19.097	23.5
3	Unknown	9	25.417	2932999	38903	30.632	22.4
4	Unknown	9	30.763	2969321	51530	31.011	29.7

17g catalyzed by **9b**

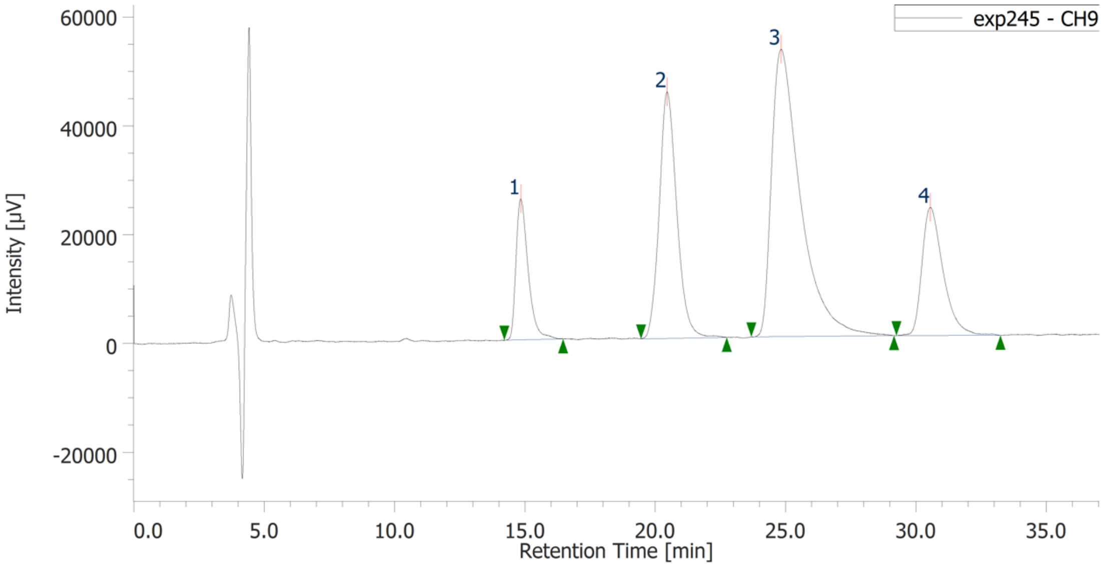

#	Peak Name	CH	tR [min]	Area [µV·sec]	Height [µV]	Area%	Height%
1	Unknown	9	14.837	840348	25912	10.050	17.6
2	Unknown	9	20.447	2124827	45312	25.410	30.7
3	Unknown	9	24.820	4066555	52792	48.631	35.8
4	Unknown	9	30.543	1330323	23529	15.909	15.9

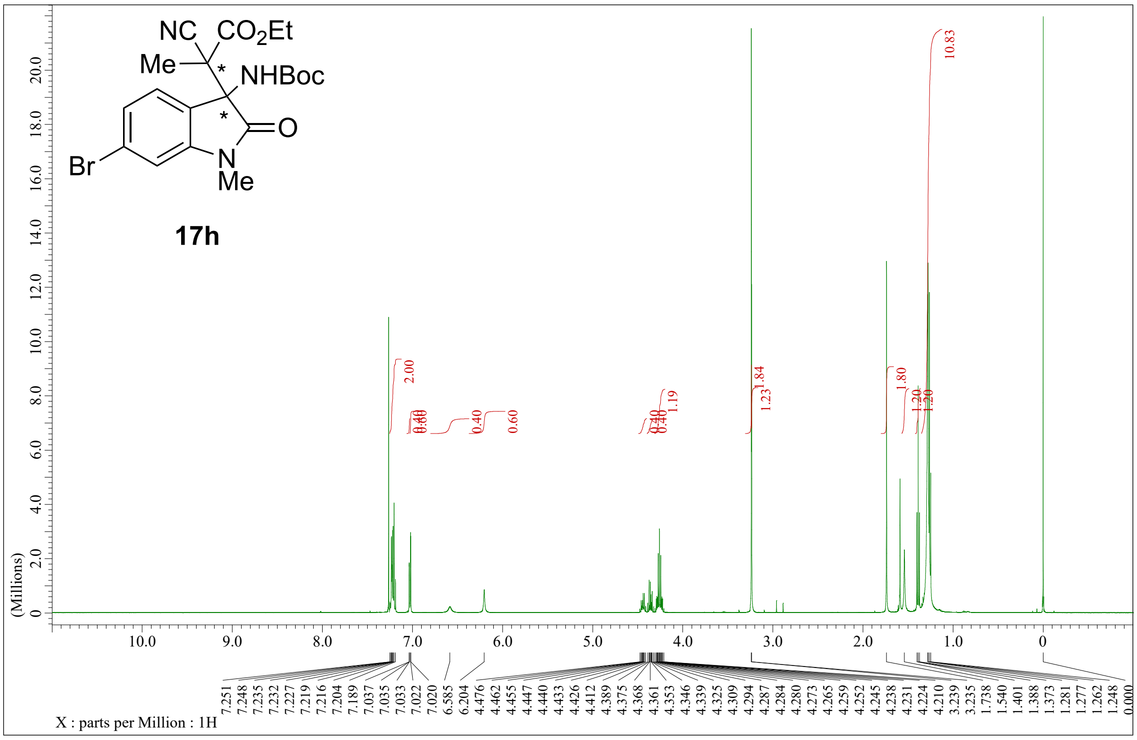
NC
CO2Et
Me
NHBoc
O
Br
N
Me
17h
(Millions)
X : parts per Million : 1H

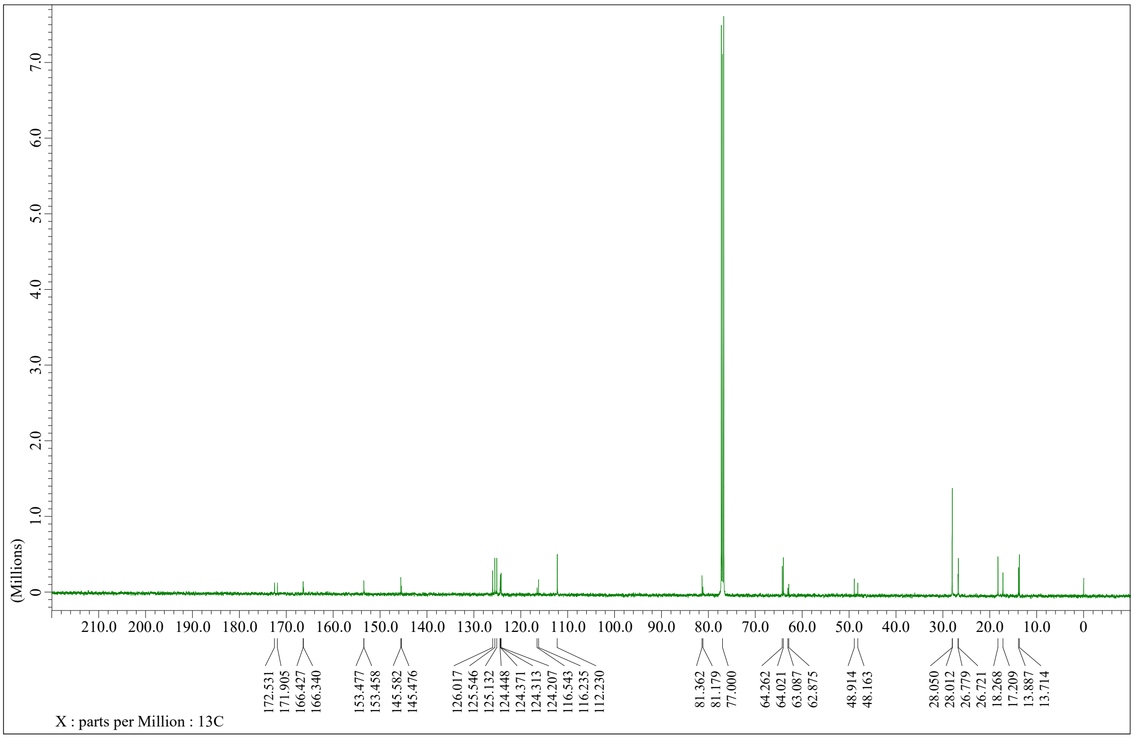
(Millions)
X : parts per Million : 13C

Racemic sample of **17h**

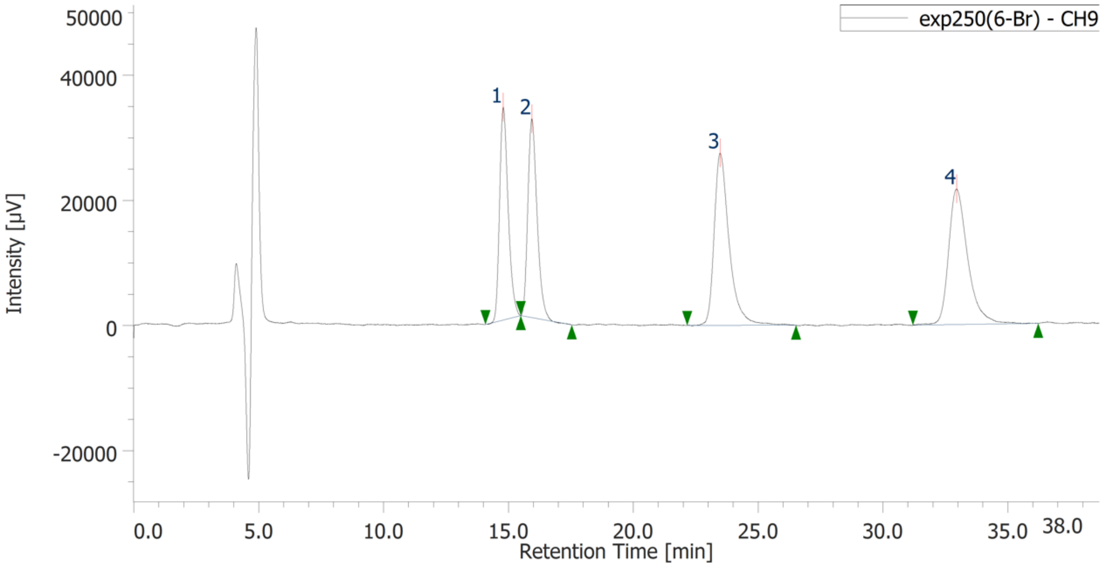

#	Peak Name	CH	tR [min]	Area [µV·sec]	Height [µV]	Area%	Height%
1	Unknown	9	14.790	811913	34004	20.606	29.6
2	Unknown	9	15.937	818118	31777	20.764	27.6
3	Unknown	9	23.470	1153717	27526	29.281	23.9
4	Unknown	9	32.937	1156400	21655	29.349	18.8

17h catalyzed by **9b**

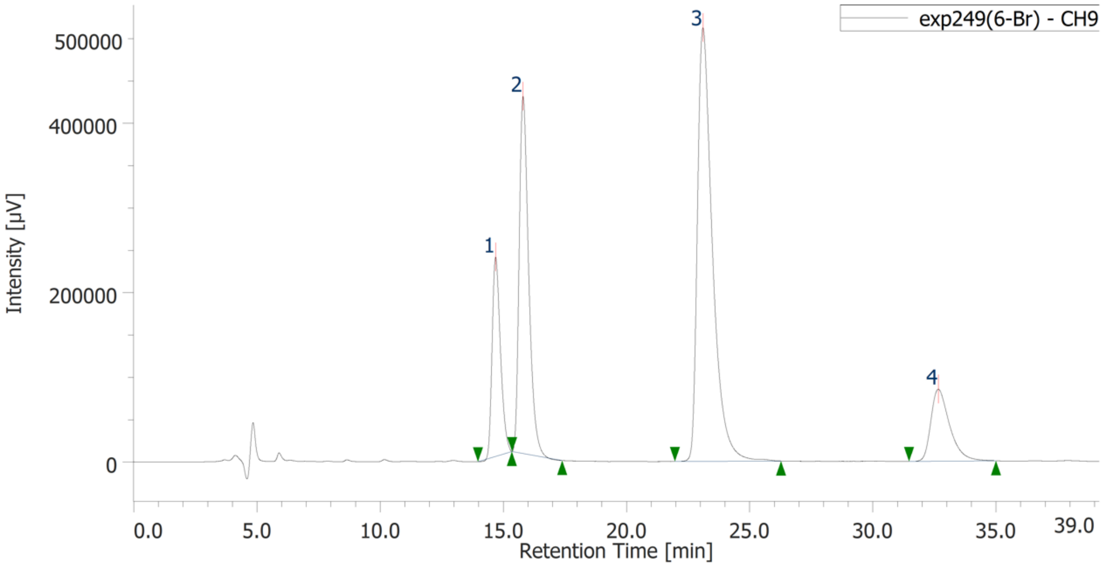

#	Peak Name	CH	tR [min]	Area [µV·sec]	Height [µV]	Area%	Height%
1	Unknown	9	14.683	5535747	235317	12.871	18.8
2	Unknown	9	15.793	11289715	421704	26.249	33.6
3	Unknown	9	23.097	21704694	512080	50.464	40.8
4	Unknown	9	32.650	4480386	85022	10.417	6.78

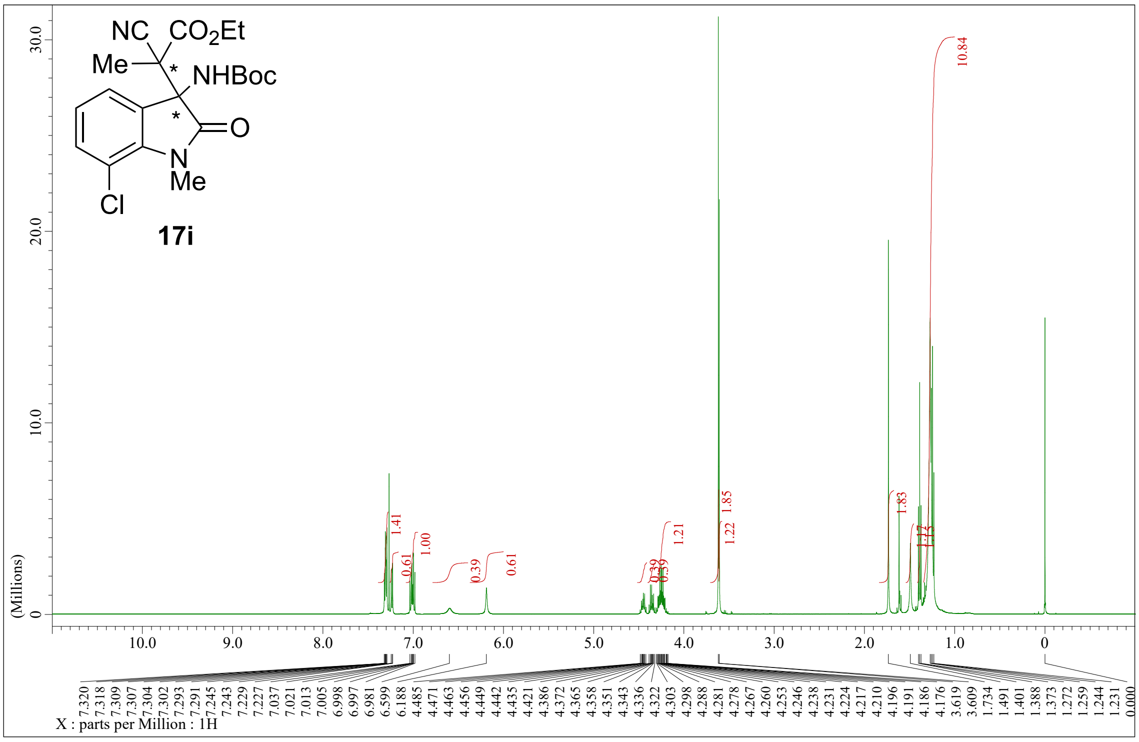
NC
CO2Et
Me
NHBoc
O
N
Me
Cl
17i
(Millions)
0
10.0
20.0
30.0
10.84
1.41
1.00
0.61
0.39
0.61
0.39
0.39
1.21
1.85
1.22
1.83
1.17
1.15
10.0 9.0 8.0 7.0 6.0 5.0 4.0 3.0 2.0 1.0 0
7.320 7.318 7.309 7.307 7.304 7.302 7.293 7.291 7.245 7.243 7.229 7.227 7.037 7.021 7.013 7.005 6.998 6.997 6.981 6.599 6.188 4.485 4.471 4.463 4.456 4.449 4.442 4.435 4.421 4.386 4.372 4.365 4.358 4.351 4.343 4.336 4.322 4.303 4.298 4.288 4.281 4.278 4.267 4.260 4.253 4.246 4.238 4.231 4.224 4.217 4.210 4.196 4.191 4.186 4.176 3.619 3.609 1.734 1.491 1.401 1.388 1.373 1.272 1.259 1.244 1.231 0.000
X : parts per Million : 1H

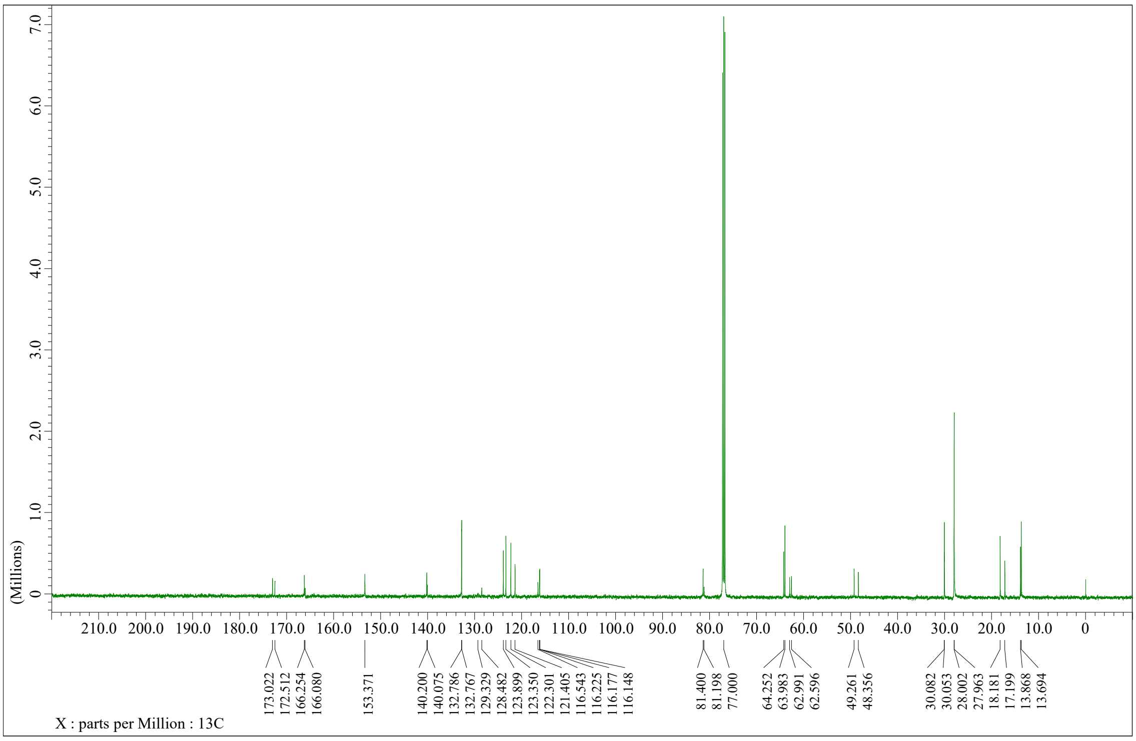
(Millions)
0
1.0
2.0
3.0
4.0
5.0
6.0
7.0
210.0 200.0 190.0 180.0 170.0 160.0 150.0 140.0 130.0 120.0 110.0 100.0 90.0 80.0 70.0 60.0 50.0 40.0 30.0 20.0 10.0 0
173.022 172.512 166.254 166.080 153.371 140.200 140.075 132.786 132.767 129.329 128.482 123.899 123.350 122.301 121.405 116.543 116.225 116.177 116.148 81.400 81.198 77.000 64.252 63.983 62.991 62.596 49.261 48.356 30.082 30.053 28.002 27.963 18.181 17.199 13.868 13.694
X : parts per Million : 13C

Racemic sample of **17i**

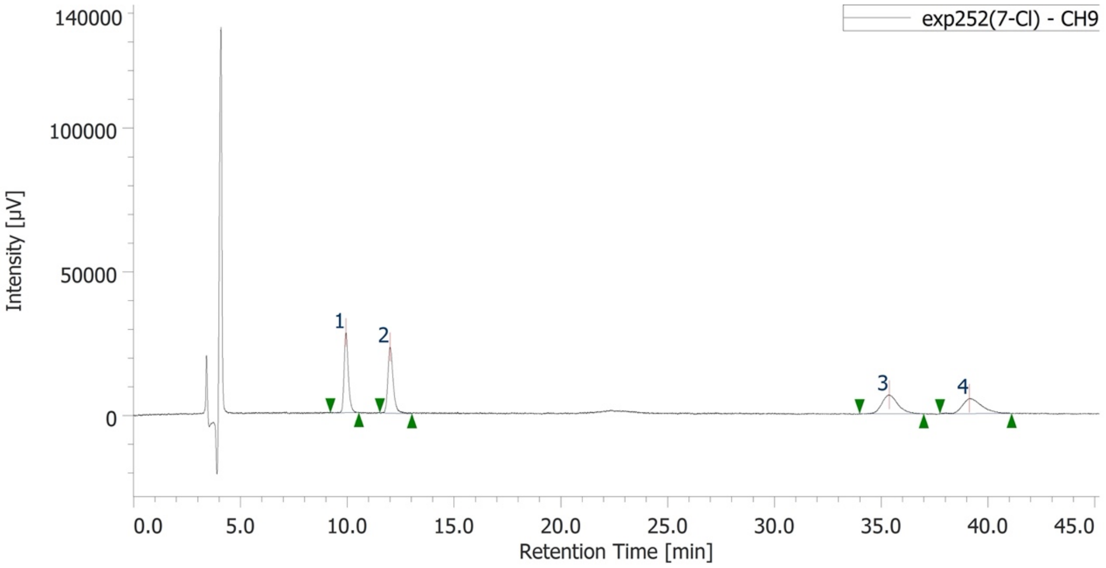

#	Peak Name	CH	tR [min]	Area [µV·sec]	Height [µV]	Area%	Height%
1	Unknown	9	9.94	381982	27830	26.469	44.446
2	Unknown	9	12.00	394278	23003	27.321	36.737
3	Unknown	9	35.37	333634	6521	23.119	10.415
4	Unknown	9	39.12	333247	5261	23.092	8.402

17i catalyzed by **9b**

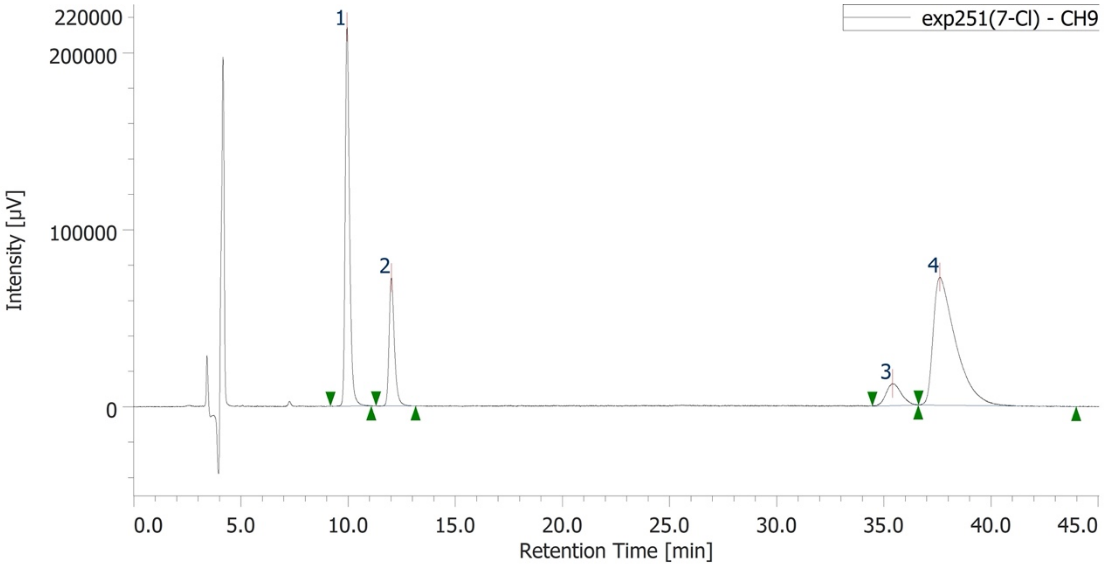

#	Peak Name	CH	tR [min]	Area [µV·sec]	Height [µV]	Area%	Height%
1	Unknown	9	9.95	3039667	214200	29.862	57.668
2	Unknown	9	12.01	1245688	72482	12.238	19.514
3	Unknown	9	35.39	619828	12446	6.089	3.351
4	Unknown	9	37.61	5273751	72311	51.810	19.468

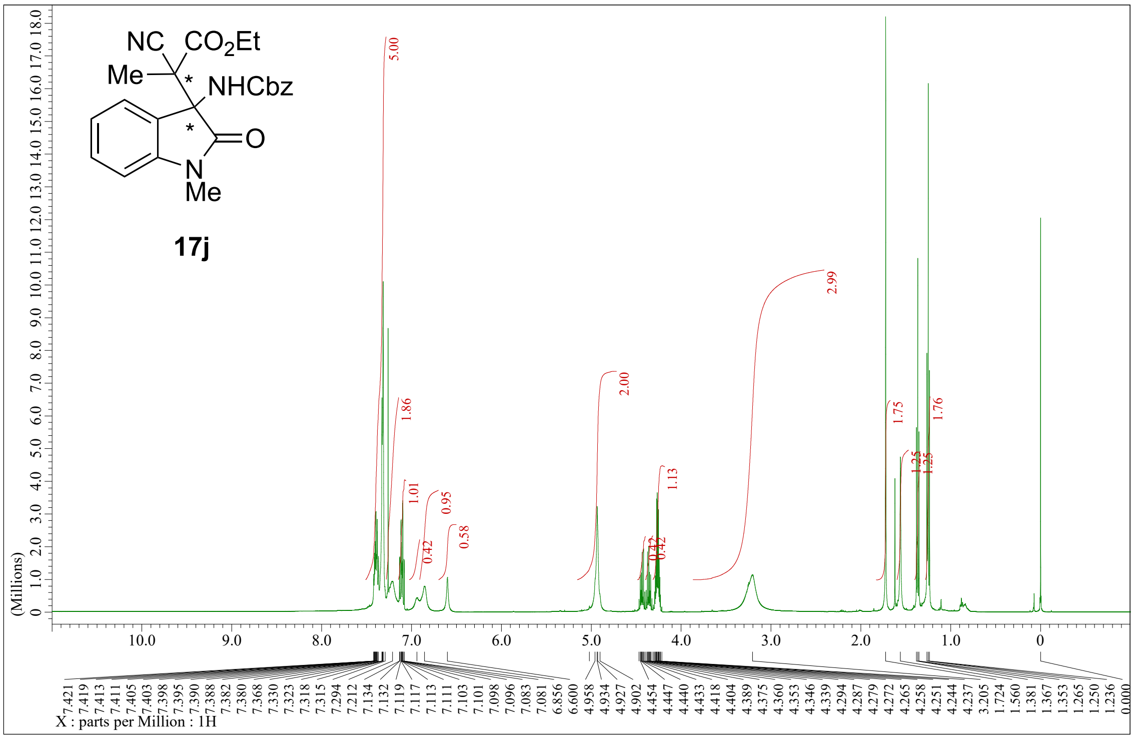
NC CO2Et
Me
NHCbz
O
N
Me
17j
(Millions)
5.00
1.86
1.01
0.42
0.95
0.58
2.00
0.42
0.42
1.13
2.99
1.75
1.25
1.25
1.76
7.421 7.419 7.413 7.411 7.405 7.403 7.398 7.395 7.390 7.388 7.382 7.380 7.368 7.330 7.323 7.318 7.315 7.294 7.212 7.134 7.132 7.119 7.117 7.113 7.111 7.103 7.101 7.098 7.096 7.083 7.081 6.856 6.600 4.958 4.934 4.927 4.902 4.454 4.447 4.440 4.433 4.418 4.404 4.389 4.375 4.360 4.353 4.346 4.339 4.294 4.287 4.279 4.272 4.265 4.258 4.251 4.244 4.237 3.205 1.724 1.560 1.381 1.367 1.353 1.265 1.250 1.236 0.000
X : parts per Million : 1H

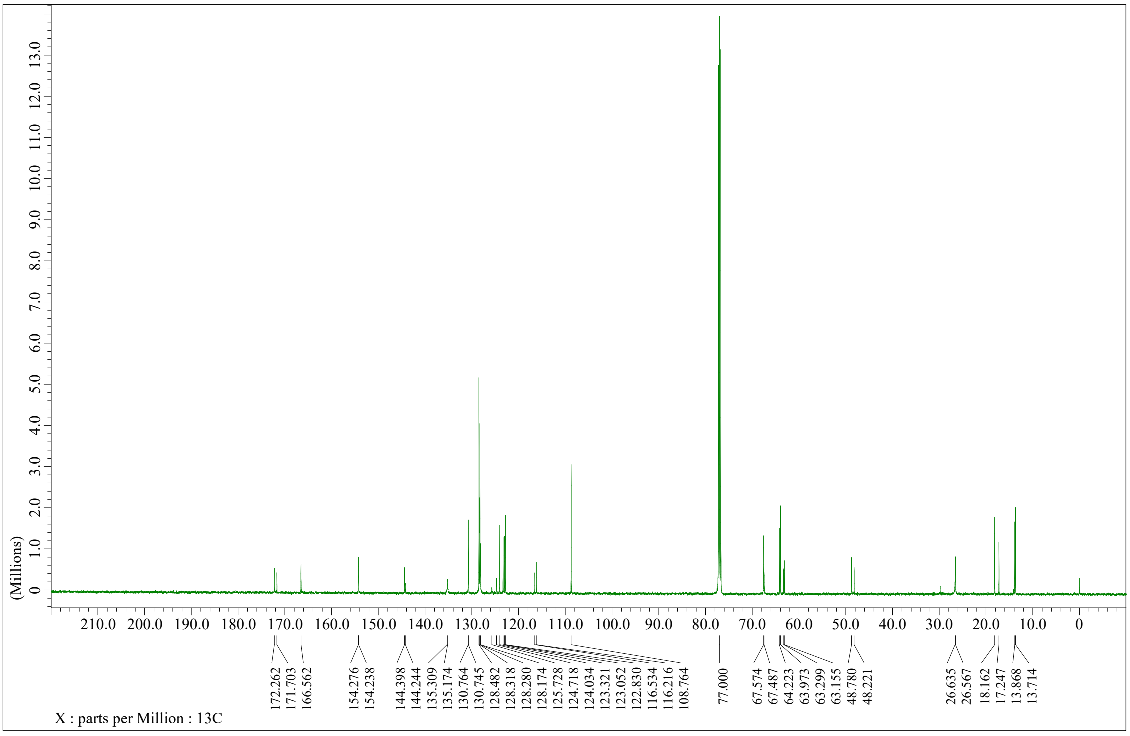
(Millions)
172.262 171.703 166.562 154.276 154.238 144.398 144.244 135.309 135.174 130.764 130.745 128.482 128.318 128.280 128.174 125.728 124.718 124.034 123.321 123.052 122.830 116.534 116.216 108.764 77.000 67.574 67.487 64.223 63.973 63.299 63.155 48.780 48.221 26.635 26.567 18.162 17.247 13.868 13.714
X : parts per Million : 13C

Racemic sample of **17j**

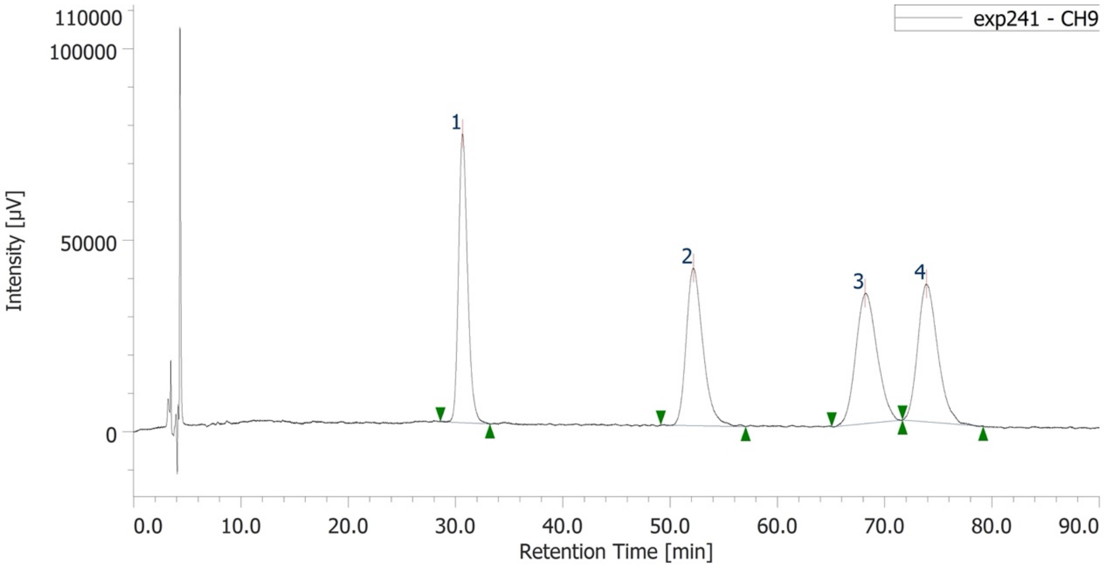

#	Peak Name	CH	tR [min]	Area [µV·sec]	Height [µV]	Area%	Height%
1	Unknown	9	30.64	4412578	75442	24.241	40.441
2	Unknown	9	52.15	4459153	41117	24.497	22.041
3	Unknown	9	68.16	4689049	34038	25.759	18.246
4	Unknown	9	73.88	4642429	35952	25.503	19.272

17j catalyzed by **9b**

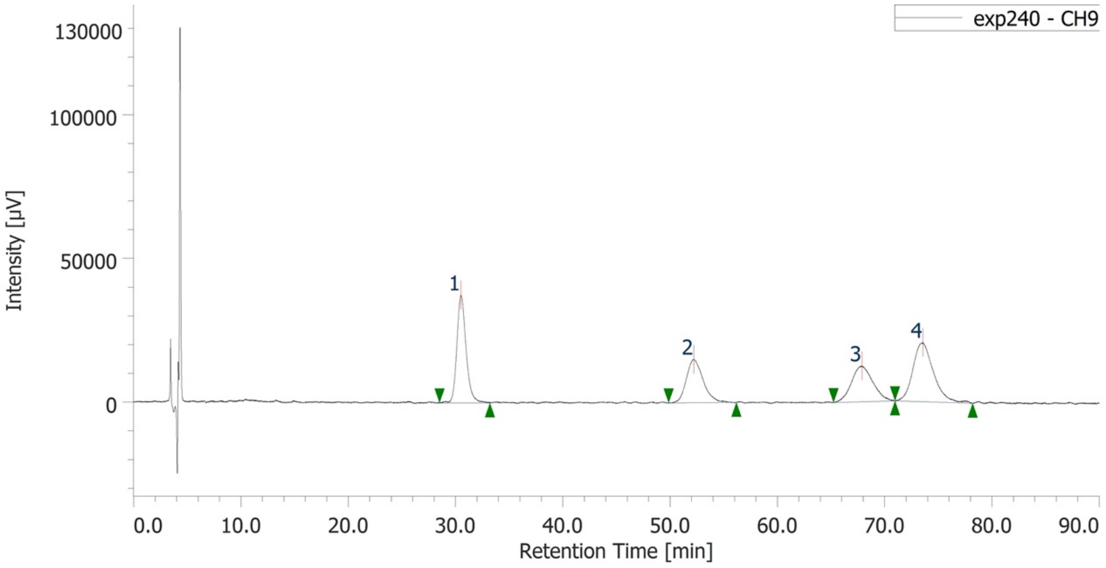

#	Peak Name	CH	tR [min]	Area [µV·sec]	Height [µV]	Area%	Height%
1	Unknown	9	30.49	2233170	37491	26.987	43.832
2	Unknown	9	52.20	1630774	15097	19.707	17.651
3	Unknown	9	67.88	1703628	12439	20.588	14.543
4	Unknown	9	73.53	2707426	20505	32.718	23.974

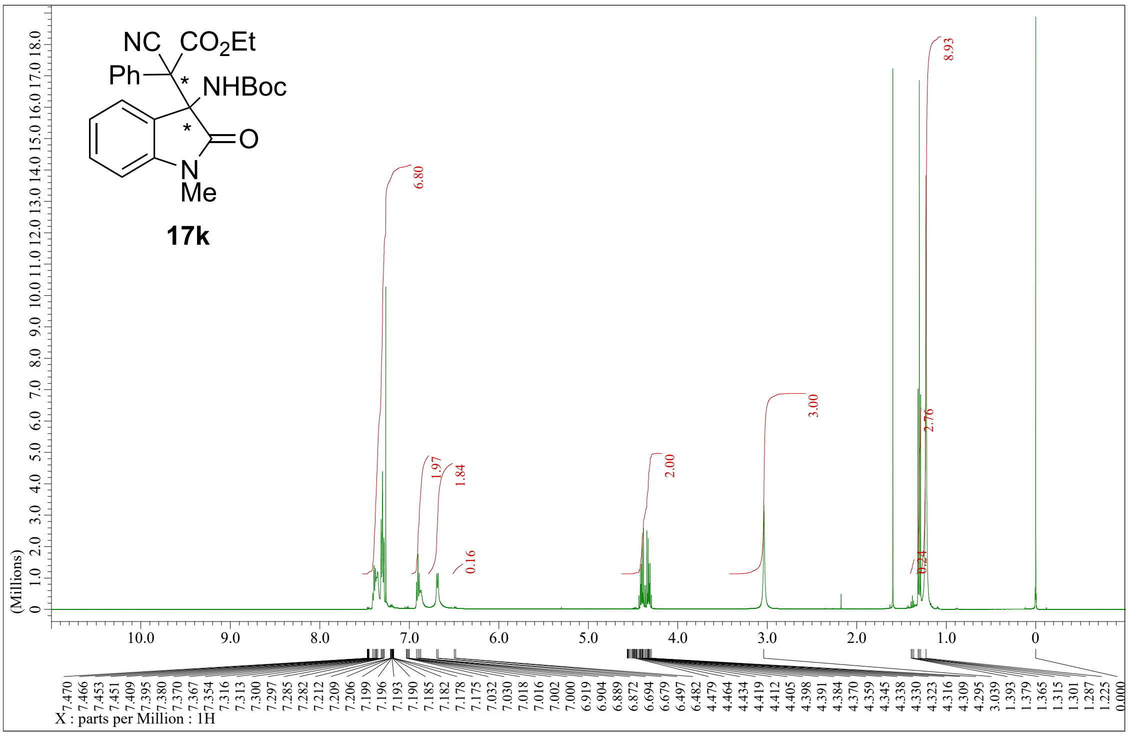

NC CO2Et
Ph
NHBoc
O
N
Me
17k
(Millions)
6.80
1.97
1.84
0.16
2.00
3.00
2.76
0.24
8.93
7.470 7.466 7.453 7.451 7.409 7.395 7.380 7.370 7.367 7.354 7.316 7.313 7.300 7.297 7.285 7.282 7.212 7.209 7.206 7.199 7.196 7.193 7.190 7.185 7.182 7.178 7.175 7.032 7.030 7.018 7.016 7.002 7.000 6.919 6.904 6.889 6.872 6.694 6.679 6.497 6.482 4.479 4.464 4.434 4.419 4.412 4.405 4.398 4.391 4.384 4.370 4.359 4.345 4.338 4.330 4.323 4.316 4.309 4.295 3.039 1.393 1.379 1.365 1.315 1.301 1.287 1.225 0.000
X : parts per Million : 1H

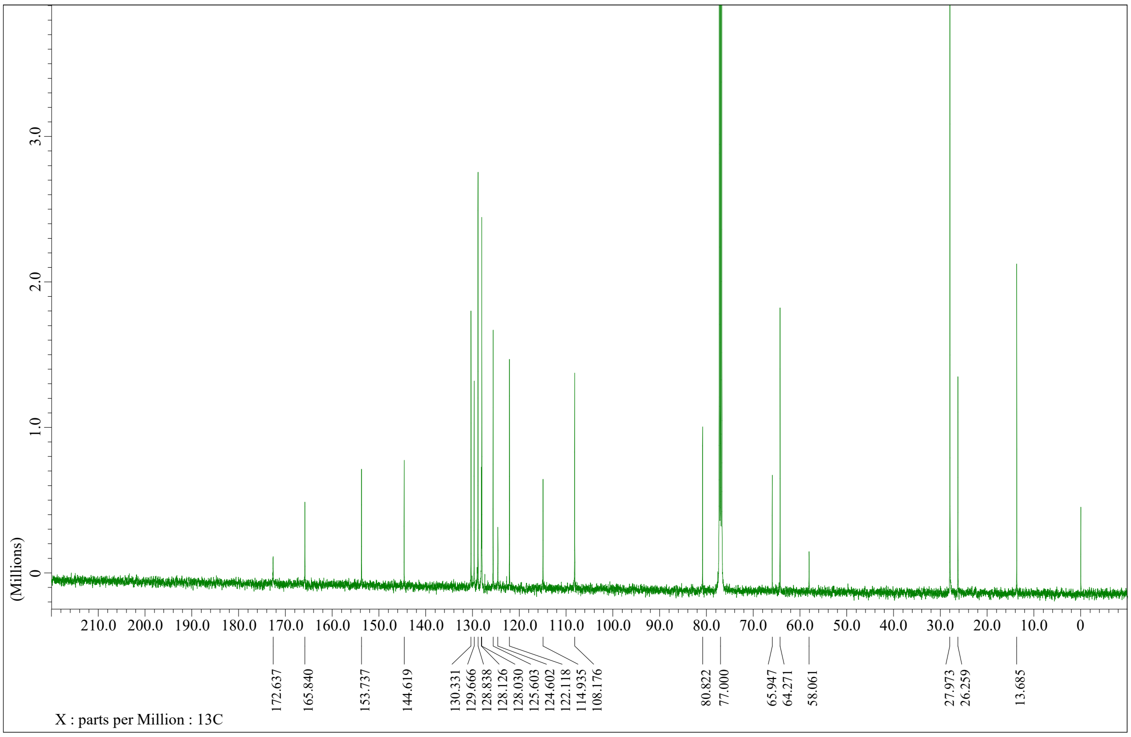

(Millions)
172.637
165.840
153.737
144.619
130.331 129.666 128.838 128.126 128.030 125.603 124.602 122.118 114.935 108.176
80.822
77.000
65.947 64.271
58.061
27.973 26.259
13.685
X : parts per Million : 13C

Racemic sample of **17k**

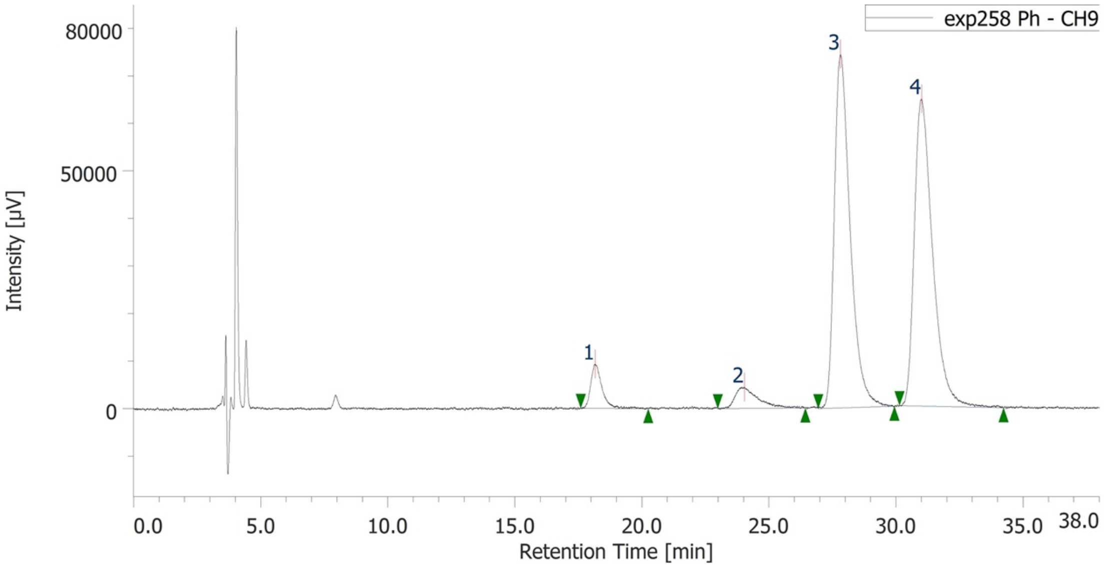

#	Peak Name	CH	tR [min]	Area [μV·sec]	Height [μV]	Area%	Height%
1	Unknown	9	18.15	288780	9334	4.101	6.109
2	Unknown	9	24.03	282570	4475	4.013	2.929
3	Unknown	9	27.81	3232507	74352	45.910	48.666
4	Unknown	9	31.01	3237174	64619	45.976	42.296

17k catalyzed by **9b**

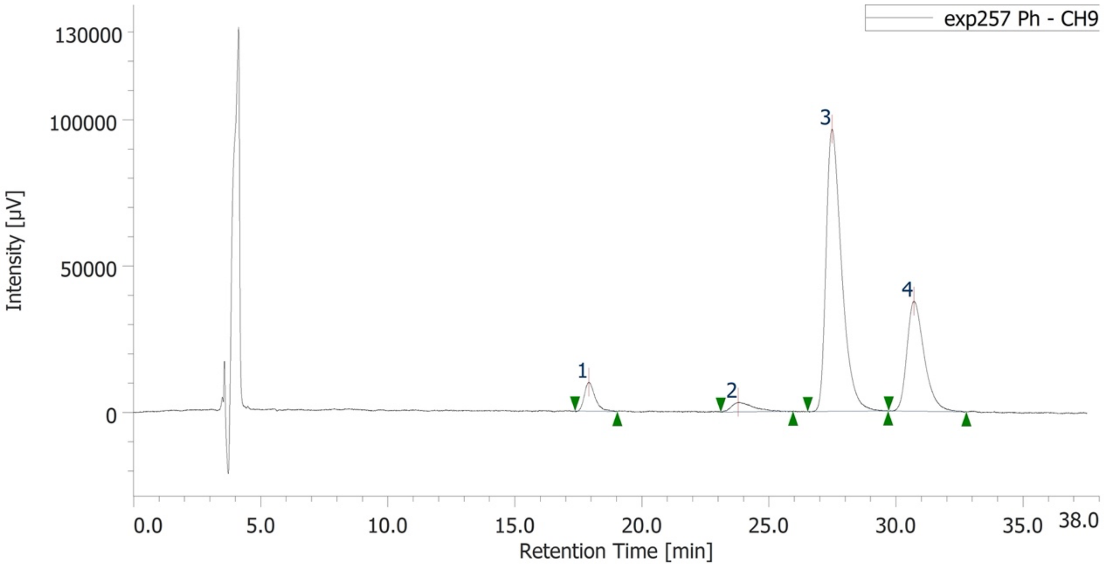

#	Peak Name	CH	tR [min]	Area [μV·sec]	Height [μV]	Area%	Height%
1	Unknown	9	17.91	290599	9937	4.560	6.750
2	Unknown	9	23.78	202682	3347	3.180	2.273
3	Unknown	9	27.48	4101854	96341	64.361	65.440
4	Unknown	9	30.70	1778075	37594	27.899	25.536

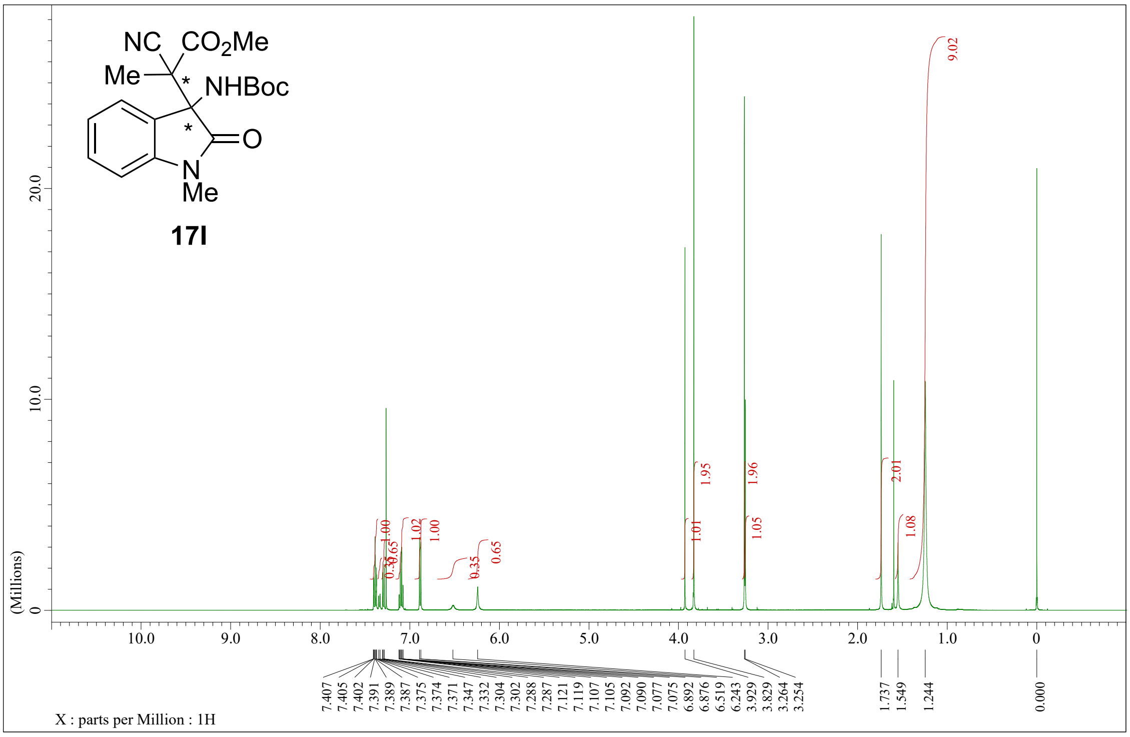
NC CO2Me
Me
NHBoc
O
N
Me
17l
X : parts per Million : 1H
(Millions)

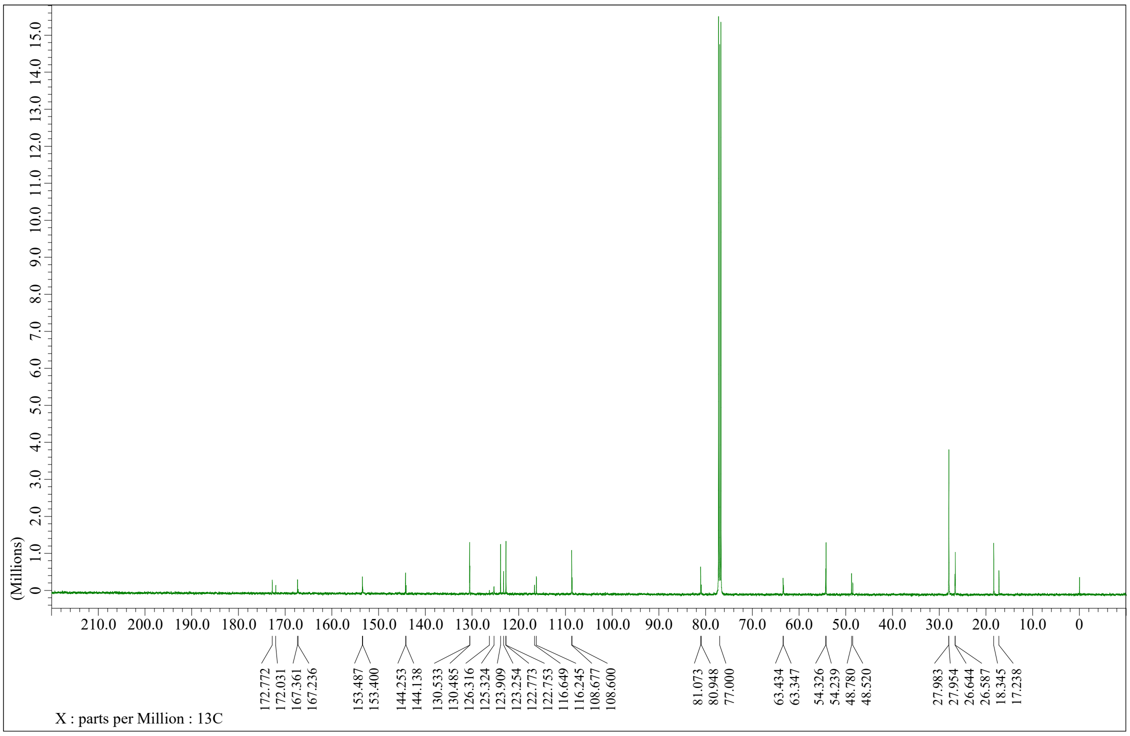
X : parts per Million : 13C
(Millions)

Racemic sample of **17l**

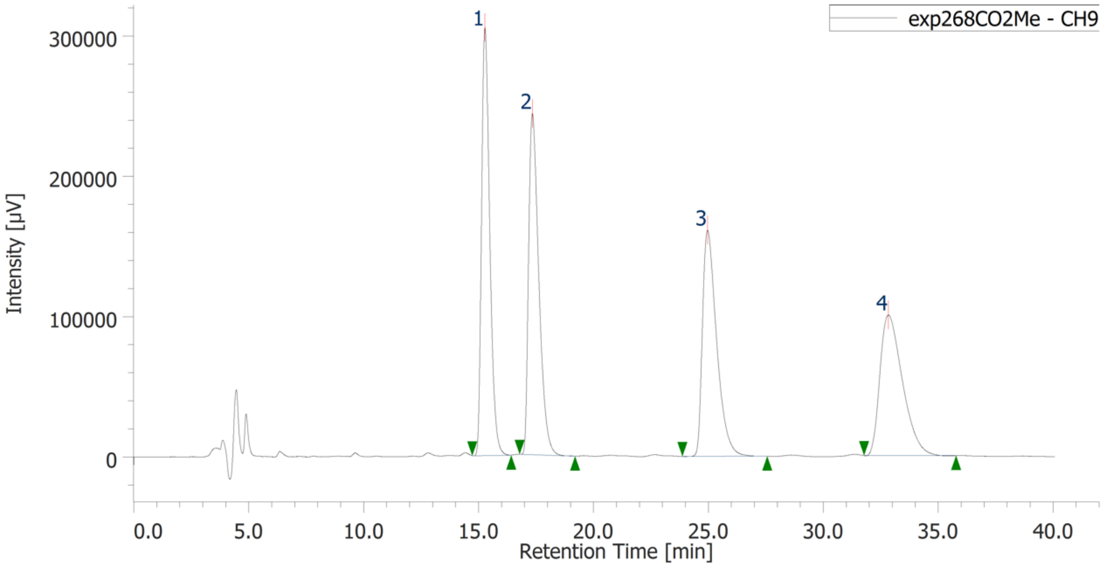

#	Peak Name	CH	tR [min]	Area [µV·sec]	Height [µV]	Area%	Height%
1	Unknown	9	15.273	7562205	304797	26.254	37.7
2	Unknown	9	17.337	7508870	242997	26.069	30.0
3	Unknown	9	24.953	6914486	161117	24.005	19.9
4	Unknown	9	32.827	6818418	100129	23.672	12.4

17l catalyzed by **9b**

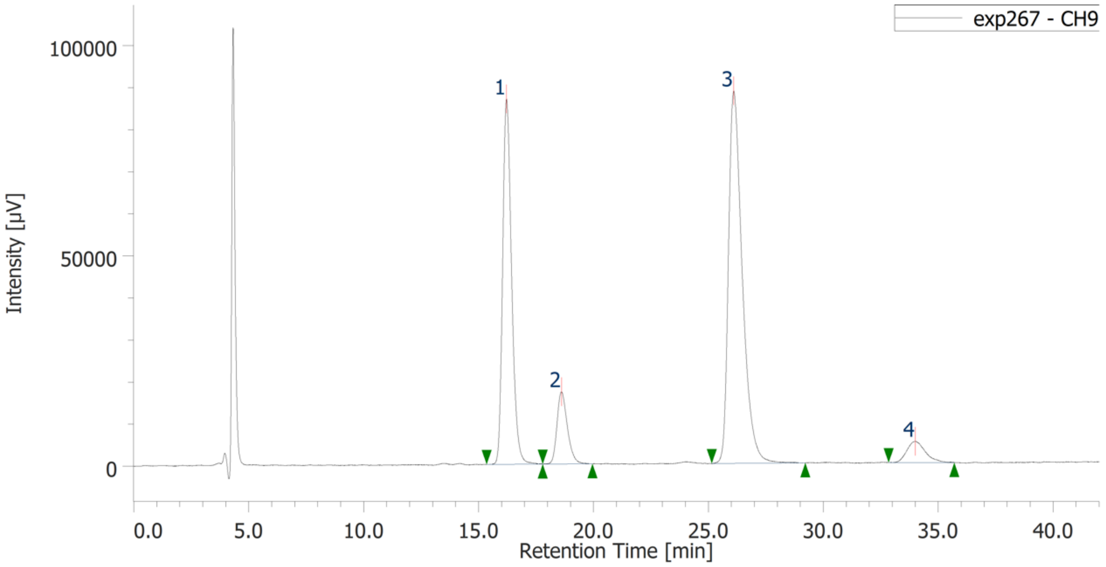

#	Peak Name	CH	tR [min]	Area [µV·sec]	Height [µV]	Area%	Height%
1	Unknown	9	16.213	2351554	86797	34.187	43.9
2	Unknown	9	18.603	540695	17163	7.861	8.69
3	Unknown	9	26.097	3702801	88505	53.832	44.8
4	Unknown	9	33.997	283451	5043	4.121	2.55

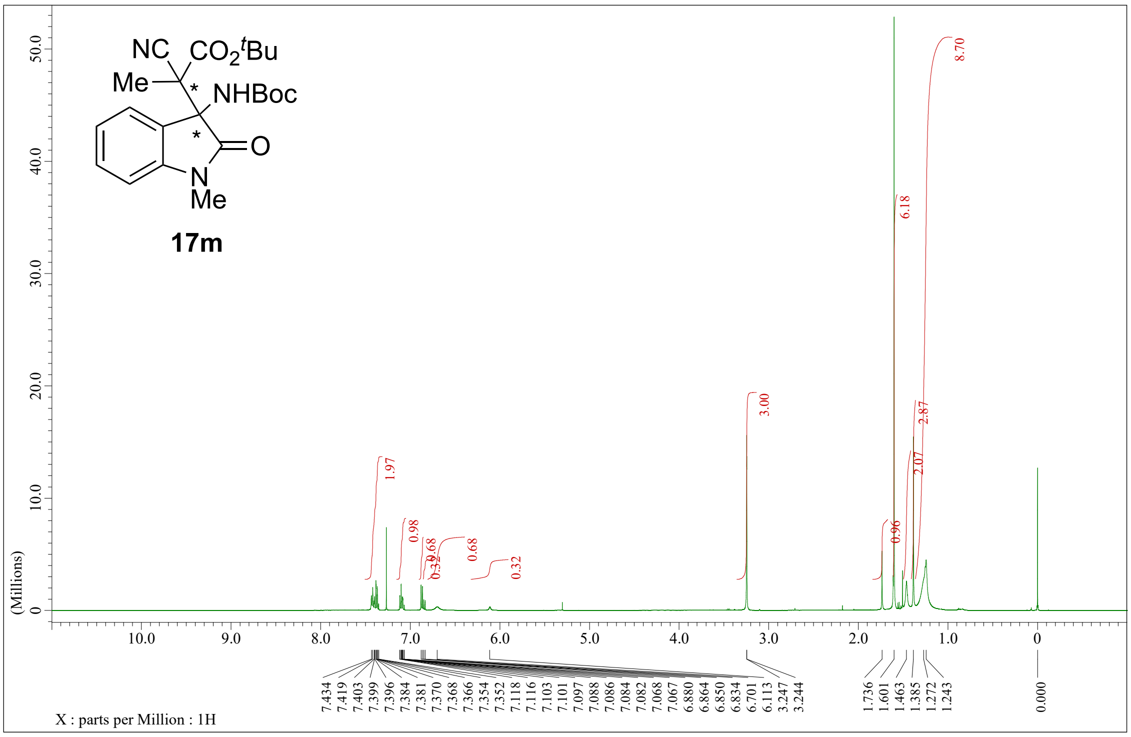
NC CO2tBu
Me
NHBoc
O
N
Me
17m
1.97
0.98
0.32
0.68
0.68
0.32
3.00
0.96
6.18
2.07
2.87
8.70
7.434 7.419 7.403 7.399 7.396 7.384 7.381 7.370 7.368 7.366 7.354 7.352 7.118 7.116 7.103 7.101 7.097 7.088 7.086 7.084 7.082 7.068 7.067 6.880 6.864 6.850 6.834 6.701 6.113 3.247 3.244
1.736 1.601 1.463 1.385 1.272 1.243
0.000
(Millions)
X : parts per Million : 1H

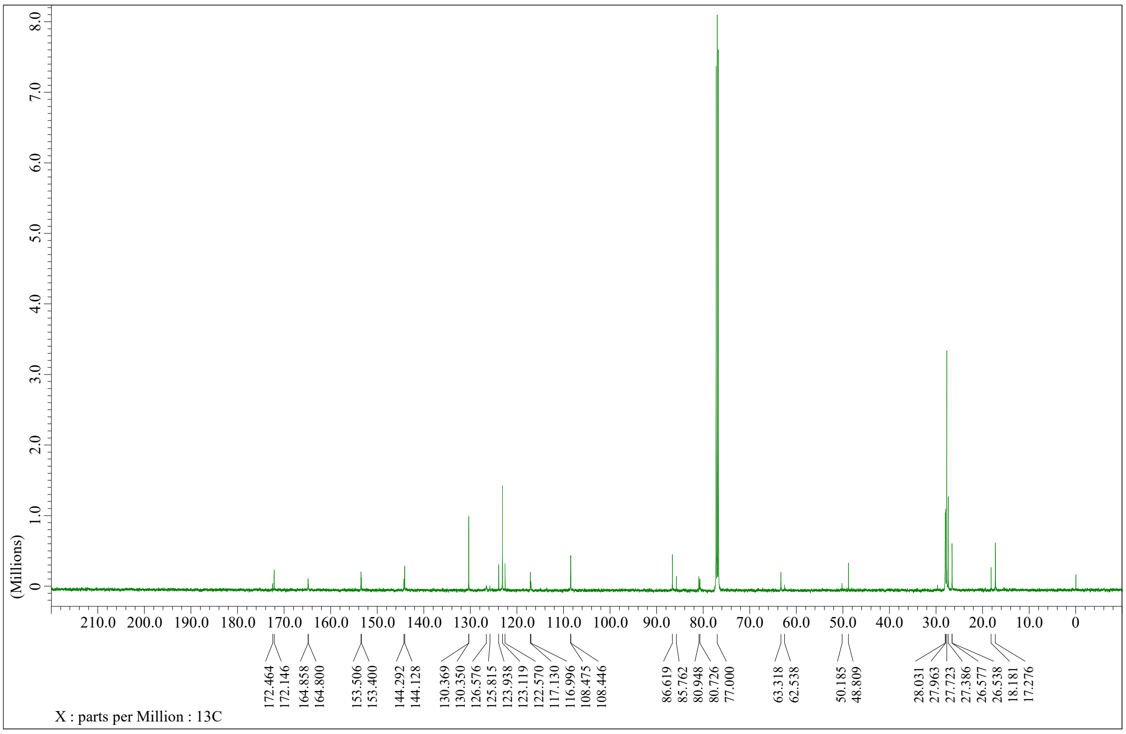
172.464 172.146 164.858 164.800 153.506 153.400 144.292 144.128 130.369 130.350 126.576 125.815 123.938 123.119 122.570 117.130 116.996 108.475 108.446
86.619 85.762 80.948 80.726 77.000 63.318 62.538 50.185 48.809
28.031 27.963 27.723 27.386 26.577 26.538 18.181 17.276
(Millions)
X : parts per Million : 13C

Racemic sample of **17m**

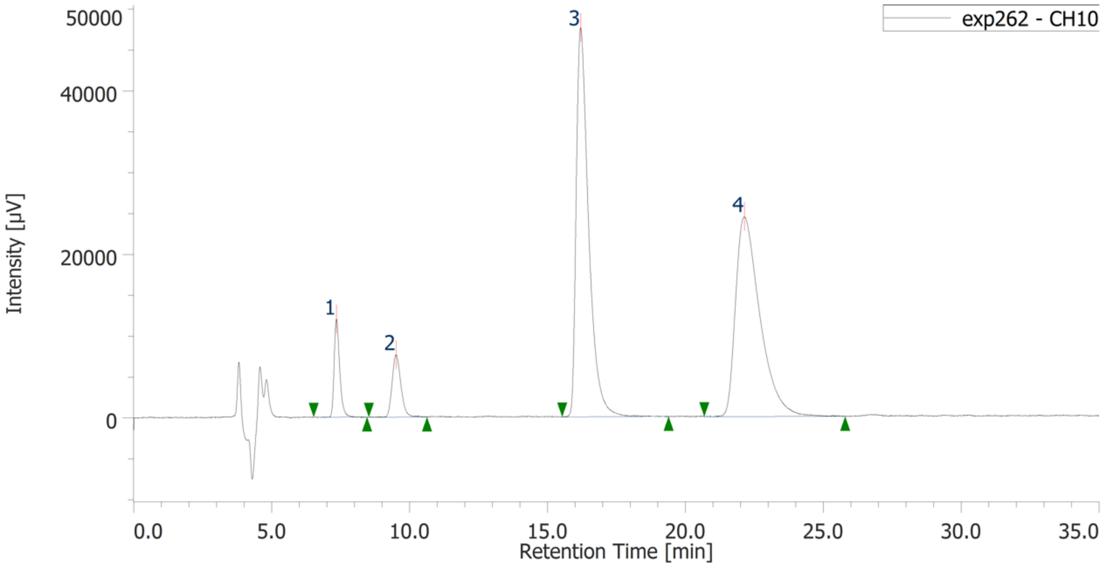

#	Peak Name	CH	tR [min]	Area [µV·sec]	Height [µV]	Area%	Height%
1	Unknown	10	7.347	168123	11995	5.061	13.1
2	Unknown	10	9.503	168691	7618	5.078	8.31
3	Unknown	10	16.197	1488128	47588	44.795	51.9
4	Unknown	10	22.133	1497156	24421	45.067	26.7

17m catalyzed by **9b**

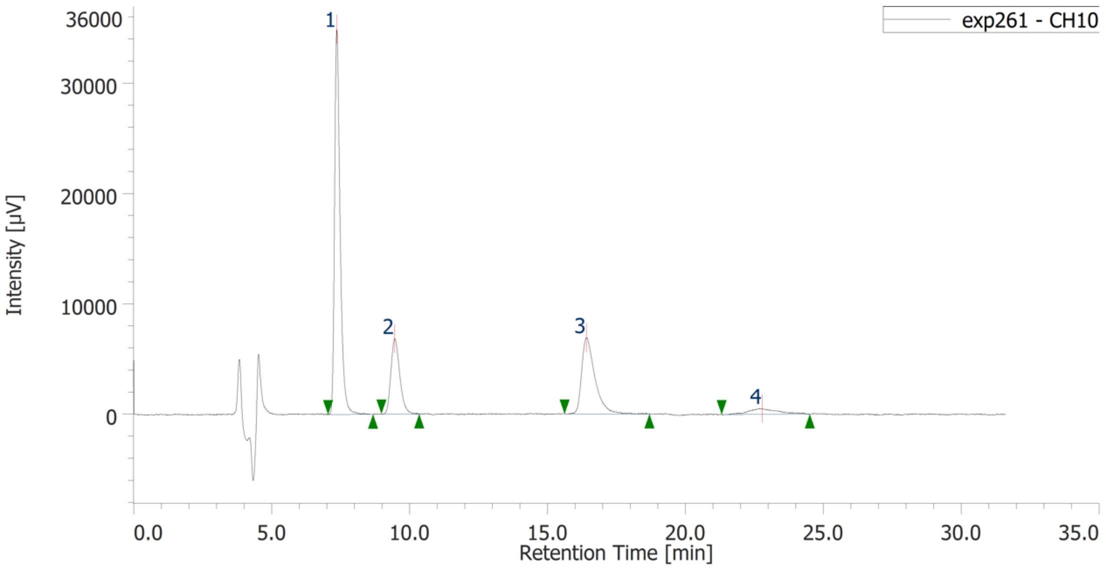

#	Peak Name	CH	tR [min]	Area [µV·sec]	Height [µV]	Area%	Height%
1	Unknown	10	7.360	487877	34916	53.622	71.0
2	Unknown	10	9.457	152332	6840	16.743	13.9
3	Unknown	10	16.403	230839	6909	25.371	14.0
4	Unknown	10	22.773	38789	518	4.263	1.05
